# Single-Determinant Ground State in Ce^4+^ Imidophosphorane Complexes

**DOI:** 10.1021/acs.inorgchem.5c03376

**Published:** 2025-12-11

**Authors:** Haruko Tateyama, Can Liao, Grant R. Wilkinson, Arun Ramanathan, Lucia Amidani, Elena Bazarkina, Florian Ressnik, Kaitlyn S. Engle, John Bacsa, Dimosthenis Sokaras, Kristina O. Kvashnina, Xiaosong Li, Henry S. La Pierre

**Affiliations:** † School of Chemistry and Biochemistry, 1372 Georgia Institute of Technology, Atlanta, Georgia 30332-0400, United States; ‡ Department ofchemistry, 7284 University of Washington, Seattle, Washington 98195, United States; § 55553 The Rossendorf Beamline at ESRF, The European Synchrotron, Grenoble, Cedex 9 38043, France; ∥ Institute of Resource Ecology, Helmholtz Zentrum Dresden Rossendorf (HZDR), Dresden 01314, Germany; ⊥ Stanford Synchrotron Radiation Lightsource, SLAC National Accelerator Laboratory, Menlo Park, California 94025, United States; # Nuclear and Radiological Engineering and Medical Physics Program, School of Mechanical Engineering, Georgia Institute of Technology, Atlanta, Georgia 30332-0400, United States

## Abstract

X-ray spectroscopy techniques are critical in the electronic structure analysis of high-valent lanthanides. The interpretation of multipeaked features at the lanthanide *L*_3_-edge has remained a challenging question, as it is observed across a range of material classes. A series of structurally related Ce^4+^complexes were prepared to probe the potential ligand field perturbation of the ground state within the series. The tuning of relative 4*f* and ligand orbital energies in homoleptic and heteroleptic tetravalent Ce imidophosphorane complexes is achieved through ligand derivatization and is clearly demonstrated by UV–vis spectroscopy and electrochemically measured redox potentials. However, Ce *L*_3_-edge high-energy resolution fluorescence-detected (HERFD) X-ray absorption near-edge structure (XANES) spectra present features at consistent numbers and energies across the range of complexes. Resonant inelastic X-ray scattering (RIXS) is employed to visualize the observed features in the HERFD-XANES spectra. Large complete active space configuration interaction singles and doubles (CASCISD) calculations demonstrate that the ground-state wave function of all complexes can be described employing a single determinant. As a result, the multielectron feature at the *L*_3_-edge observed in this study for the Ce^4+^ imidophosphorane complexes is described as excited-state multiconfigurational behavior that is independent of ligand variation, in systems where the ground state is described using the simple single determinant wave function.

## Graphical abstract

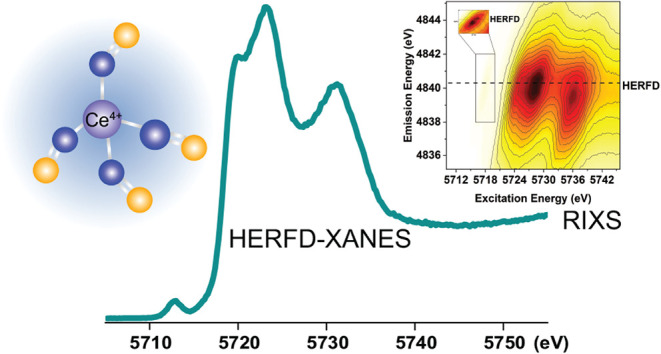

## Introduction

The characteristic double-peak feature observed in the Ce *L*_3_-edge X-ray absorption near-edge structure (XANES) across various material classes, including intermetallic phases,^1,2^ formally tetravalent insulating oxides,^3–5^ fluorides,^6,7^ and molecular complexes,^8–15^ has attracted interest in the role of multiconfigurational behavior in the ground and excited states. However, the interpretations of the XANES spectra have led to ambiguity in the formal oxidation state of these systems. *L*_3_-edge XANES, arising from 2*p*_3/2_ → 5*d*_5/2_ transitions, is sensitive to the lanthanide oxidation state, changing spectral features as the lanthanides oxidize from 3+ to 4+.^16,17^ A single white line feature is seen in Ln^3+^ complexes whereas Ln^4+^ complexes demonstrate a double-peak shifted to higher energies.^1^ Empirically, the “double-peak” is a diagnostic feature of the tetravalent oxidation state in lanthanide complexes and insulating materials.^7,18^

Historically, the double-peak feature at the *L*_3_-edge was rationalized within the framework of single-ion Anderson impurity model (SIAM) by Kotani and coworkers.^1,19^ Such theoretical treatment invokes a mixed-oxidation state ground state to account for the significant metal–ligand covalency in order to reproduce the double-peak feature arising from the electron correlation in a charge transfer insulator.^1,20^ This theoretical framework is also used to reproduce the double-peak features at the *L*_3_-edge arising from heavy-Fermion behavior in cerium-based conducting intermetallic phases.^1,19,21,22^ The latter analysis has captured the imagination of molecular lanthanide chemists and such heavy-Fermion behavior in a molecular system may have been physically realized in cerocene, [Ce­(COT)_2_]­(COT = C_8_H_8_^2–^), and related complexes, where the ground state is simply described as an admixture of a stronger 4f^1^(Ce^3+^) and 4f^0^(Ce^4+^).^8,23–31^ In this understanding of cerocene, a chemical probe of the physicality of a single ion mixed-valence ground state could be established through the systematic variance of ligand properties and donor capabilities. The basis of the single ion mixed-valence ground state depends on the balance of the relative energies of the metal valence f- and d-orbitals and the ligand donors. Therefore, within this theoretical framework, the admixture of valence states should be highly sensitive to ligand modification.^32^

Alternatively, the significant ligand–metal covalency arising in tetravalent lanthanides can be described by computational techniques including TDDFT^10,22,33,34^ and *ab initio* ^35–37^ methods. These approaches have established a bonding model that describes tetravalent Ce in molecular complexes and extended solids distinct from the heavy-Fermion behavior in lanthanide and actinide intermetallics. These calculations describe systems that are molecular analogs of charge transfer insulators well, wherein strong static and dynamic correlation are paired with conventional covalent donation bonding.^36,38^ To this end, there is a growing body of spectroscopic and magnetic data on formally tetravalent lanthanide molecular complexes which are described employing a single-configurational ground state model, most intuitive to chemists.^8–11,39,40^

It is crucial to recognize that the two models presented above, the SIAM model with single ion mixed-valence states and the single determinant model, are both valid descriptions of the electronic structure of tetravalent cerium with the ability to reproduce the characteristic double peak feature. Both theoretical approaches^35,36,38^ as well as experimental work have shown that these approaches are a matter of perspective.^35,36^ However, it is suggested that depending on the nature of the system (i.e., charge transfer insulator^41^ vs intermetallics^22^) the physical mechanisms (or the relative contribution of competing mechanisms) may vary and suggests that chemical perturbation is a means to parse these contributions to the observed features. Therefore, consolidation of collective understanding and physical description of the bonding and electronic structure of tetravalent cerium-based complexes is necessary to facilitate clear communication among the many fields interested in this behavior.

In order to understand the Ce *L*_3_-edge XANES spectra of Ce^4+^ imidophosphorane complexes within a conserved geometry and ligand donor type, a series of Ce^4+^ complexes with a uniform coordination geometry and systematic ligand variation were studied. We have previously developed^12,42^ and applied^43,44^ a library of imidophosphorane ligands that have enabled the isolation of 4+ Ce,^12,13,45^ Pr,^46,47^ and Tb complexes^42^ as well as a 5+ Pr complex.^48^ Herein, we apply HERFD-XANES to a series of tetrahomoleptic imidophosphorane Ce^4+^ complexes (**1** to **4**), and a series of Ce^4+^ complexes supported by three [NP­(^*t*^Bu)_3_]^−^ ligands and a single I^–^, benzyl (Bn^–^), or neopentyl (Npt^–^) ligand. These complexes represent the widest range of chemically accessible Ce^4+^ complexes in a consistent coordination sphere. This study presents a well-controlled set of Ce^4+^ complexes to examine the effects of ligand modification on physical properties (as investigated through UV–vis, electrochemistry) and the *L*_3_-edge XANES and RIXS spectra.

## Experimental Details

Synthetic details are described as below. All syntheses and manipulations were conducted using air-free techniques such as Ar Schlenk line or glovebox filled with N_2_ (<0.1 ppm of O_2_/H_2_O) atmosphere. Complexes 1,^12^ 2,^13^ 4,^47^ 4-I, 4-Bn and 4-Npt^45^ were prepared following previously reported procedures. Further detailed general considerations, NMR, UV–vis, electrochemistry, crystallographic details, computational details, fits, and details on Ce *L*_3_-edge XANES, HERFD-XANES, RIXS are included in the Supporting Information.

### [(CH_2_N^t^Bu)_2_(C_6_H_5_)­P], 5a

Using a Schlenk line, C_6_H_5_PCl_2_ (7.0 mL, 1.0 equiv., 53.0 mmol) was transferred to a 500 mL Schlenk round-bottom flask with 300 mL diethyl ether (Et_2_O) and a PTFE stir bar. The reaction mixture was cooled to –20 °C with a salt ice bath. Triethylamine (37.0 mL, 5.0 equiv., 265 mmol) was added to the reaction mixture, followed by N,N’-di-*tert*-butylethylenediamine (11.78 mL, 1.05 equiv., 55.7 mmol), which was added dropwise over 15 min. A cloudy, white precipitate formed immediately. The reaction mixture was stirred at 25 °C for 16 h. Subsequently, the reaction mixture was filtered inside of the glovebox using a 60 mL fine porosity frit washed with 30 mL of Et_2_O to yield a colorless solution. Volatiles were removed *in vacuo* to yield a colorless, opaque liquid. The liquid was put in 10 mL Et_2_O, filtered through pipet filled with glass fiber filter and Celite, then further concentrated *in vacuo* to ∼ 3 mL and cooled for 16 h at –35 °C to yield colorless crystals. The supernatant was removed through decantation, and colorless crystals were dried *in vacuo*, at which point the crystals collapsed to an opaque liquid. The title compound was afforded as a colorless liquid (3.286 g, 22%). ^1^H NMR (400 MHz, THF) δ = 7.55 (t, 2H), 7.28 – 7.19 (m, 2H), 7.19–7.13 (m, 1H), 3.10 (t, 2H), 3.01 (t, 2H), 1.24 (s, 18H). ^13^C­{^1^H} NMR (101 MHz, *d*_8_-THF) δ = 148.69 (d, *J*_13*C*‑31*P*_ = 31.9 Hz), 130.88, 128.29, 128.06, 53.98, 47.22, 30.25. ^31^P­{^1^H} NMR (162 MHz, C_6_D_6_) δ = 80.75. IR: ν (cm^–1^) = 1359.95 (m), 1261.78 (w), 1238.04 (m), 1210.01 (s), 1120.40 (m), 1083.64 (m), 1024.76 (m), 994.03 (m), 974.84 (m), 940.24 (w), 866.99 (w), 837.46 (w), 783.70 (m), 744.25 (m), 697.50 (s), 686.01 (m), 652.79 (m), 640.39 (m), 580.93 (w), 507.15 (m), 449.18 (m), 421.68 (m). Elemental analysis of the air-sensitive liquid was not performed.

### K­[(CH_2_N^t^Bu)_2_(C_6_H_5_)­P=N], 5b

The compound was prepared in a one-flask, three reaction sequence. Inside of a glovebox, **5a** (3.268 g, 1.0 equiv., 11.7 mmol) was dissolved in 40 mL toluene in a 100 mL Schlenk pear flask with a PTFE stir bar. To this solution, trimethylsilyl azide (3.08 mL, 2.0 equiv, 23.5 mmol) was added with a syringe inside of the glovebox. The flask was moved to a Schlenk line, and the reaction mixture was refluxed at 125 °C for 5 days. Volatiles were removed *in vacuo*, affording a yellow-white opaque liquid. This liquid was triturated with 10 mL pentane and used in the subsequent reaction without further purification. Dry, degassed MeOH in large excess (16.5 mL, 40.0 equiv., 470 mmol) was added to the liquid product in a 100 mL Schlenk pear flask equipped with a PTFE stir bar. Using a syringe, 1 drop of H_2_SO_4_ was added to the solution. The reaction mixture was stirred at 25 °C for 2 days under static Ar to yield a yellow, clear solution. Volatiles were removed *in vacuo*, which afforded a yellow, waxy solid. This solid was triturated with pentane to give a white solid. Inside of the glovebox, the crude white solid (1.99 g) was dissolved in 5 mL of toluene with a PTFE stir bar. To this colorless solution, potassium benzyl (0.894 g, 0.58 equiv., 6.9 mmol) [calculated as 1.1 equiv. to the crude material, assuming conversion to (CH_2_N^t^Bu)_2_(C_6_H_5_)­P=NH] was added slowly upon stirring, yielding a red, clear solution. The reaction mixture was stirred at 25 °C for 16 h, affording an ivory-orange slurry. Using a 60 mL fine porosity frit, an orange-white solid was collected as a solid, which was then washed with 5 mL of pentane, and dried *in vacuo* to yield the title compound (820 mg, 2.79 mmol, 23.8%). ^1^H NMR (500 MHz, *d*_8_-tol) δ = 7.99 (t, 2H), 7.32–7.17 (m, 2H), 7.16–7.11 (m, 1H), 3.19 (t, 2H), 3.14 (t, 2H), 1.25 (s, 18H).^13^C­{^1^H} NMR (126 MHz, *d*_8_-tol) δ = 150.45 (d, *J*_13*C*‑31*P*_ = 108.5 Hz), 132.72, 127.45, 127.02 (d, *J*_13*C*‑31*P*_ = 10.9 Hz), 52.16, 42.09, 29.64. ^31^P­{^1^H} NMR (202 MHz, *d*_8_-tol) δ = 21.30. IR: ν (cm^–1^) = 3058 (w), 3017 (m), 2961 (w), 2899 (w), 2034, (w), 1977 (w), 1473 (w), 1432 (w), 1349 (w), 1196 (s), 1134 (m), 1088 (m), 1042 (m), 863 (m), 743 (m), 695 (s), 609 (s), 514 (s), 500 (w). Elemental Analysis, C_16_H_27_N_3_PK, % found (calculated): C 57.80 (57.97), H 8.15 (8.21), N 12.49 (12.68).

### Ce^4+^[(CH_2_N^t^Bu)_2_(C_6_H_5_)­P=N]_4_, 3

Inside of the glovebox, CeI_3_(THF)_4_ (88 mg, 1.0 equiv., 0.11 mmol) was dissolved in 4 mL Et_2_O in a 20 mL scintillation vial equipped with a glass stir bar. **5b** (150 mg, 4.1 equiv, 0.45 mmol) was dissolved in 4 mL of diethyl ether in another vial. The colorless solution of **5b** was added to a solution of CeI_3_(THF)_4_, and stirred for 16 h at room temperature in the dark. Upon stirring, a yellow slurry was formed. Under red light, AgI (26 mg, 1.0 equiv., 0.11 mmol) was added as a solid, and the residual AgI was added as a slurry in 1 mL THF. While the solution was stirred for 30 min, the solution turned into a green brown color. Subsequently, the solution was filtered through Celite and glass fiber, yielding a red solution. The solution was dried*in vacuo* and triturated three times with 1 mL of pentane. Red-orange SCXRD quality crystals (96 mg, 60%) were obtained from Et_2_O at –35 °C over 16 h. ^1^H NMR (500 MHz, C_6_D_6_) δ 8.46–8.27 (m, 8H), 7.39–7.28 (m, 8H), 7.25–7.18 (m, 4H), 3.20 (s, 8H), 3.04 (s, 8H), 1.45 (s, 72H).^13^C­{^1^H} NMR (126 MHz, C_6_D_6_) δ 145.75, 144.69, 133.52, 52.72, 41.26, 29.48. ^31^P­{^1^H} NMR (202 MHz, C_6_D_6_) δ = 31.18. IR: ν (cm^–1^) = 3067 (w), 3052 (w), 2965 (m), 2835 (w), 1476 (w), 1435 (w), 1389 (w), 1375 (w), 1358 (m), 1266 (w), 1244 (w), 1207 (m), 1173 (w), 1142 (w), 1110 (s), 1092 (m), 1050 (m), 1025 (w), 998 (w), 975 (m), 870 (m), 798 (m), 746 (m), 711 (m), 695 (m), 658 (w), 638 (m), 590 (w), 524 (w), 511 (s), 454 (m). Elemental analysis, C_64_H_108_CeN_12_P_4_, % found (calculated): C 58.64 (58.69), H 8.29 (8.31), N 12.69 (12.83).

## Results

A series of four-coordinate Ce^4+^ homoleptic and heteroleptic complexes in an imidophosphorane ligand field were prepared following previously reported syntheses [**1**;^12^ **2**;^13^ **4**;^47^ **4-I**; **4-Bn**; **4-Npt** .^45^ In addition to these previously reported complexes, the neutral tetrahomoleptic Ce^4+^ complex Ce­(NP­(N,N’-di-*tert*-butylethylenediamide)­Ph)_4_ (**3**) was synthesized. The following spectroscopic study encompasses seven different Ce^4+^ imidophosphorane complexes, as shown in Figure 1.

**Figure 1:**
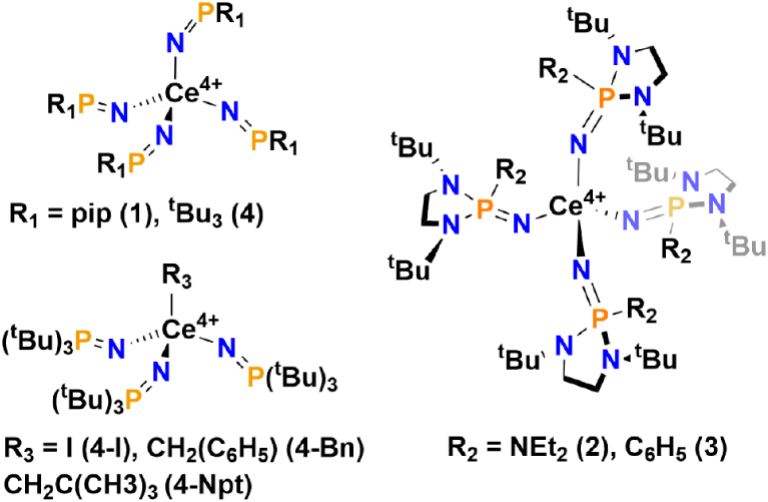
Molecular structures of **1**, **2**, **3**, **4**, **4-I**, **4-Bn**, and **4-Npt**.

Complex **3** is supported by the (NP­(N,N’-di-*tert*-butylethylenediamide)­Ph)^−^ (NP^*,*Phen*^) ligand, which is similar to the NP* ligand of **2**, but has a phenyl group rather than a diethylamide substituent. Nuclear magnetic resonance (NMR) spectroscopy and single-crystal X-ray diffraction (SC-XRD) are in agreement with the structural representation of **3** in Figure 1. Comparison of the structural features of **3** with the previously reported structures is included in the Supporting Information (Table S4).

### Electronic Absorption Spectroscopy

UV–vis absorption spectra of the Ce^4+^ imidophosphorane complexes reveal ligand-to-metal charge transfer (LMCT) features between 326 to 391 nm (Figure 2).^12,13,45,47^ These features span a range of 65 nm (0.56 eV) and have molar extinction coefficients between 5500 M^–1^cm^–1^ and 16100 M^–1^cm^–1^. Current (*vide infra*) and previous theoretical studies have shown that these LMCT features arise mostly from charge transfer out of the coordinating N atom on the imidophosphorane ligands into Ce 4*f*-orbitals.^12,13,45^

**Figure 2:**
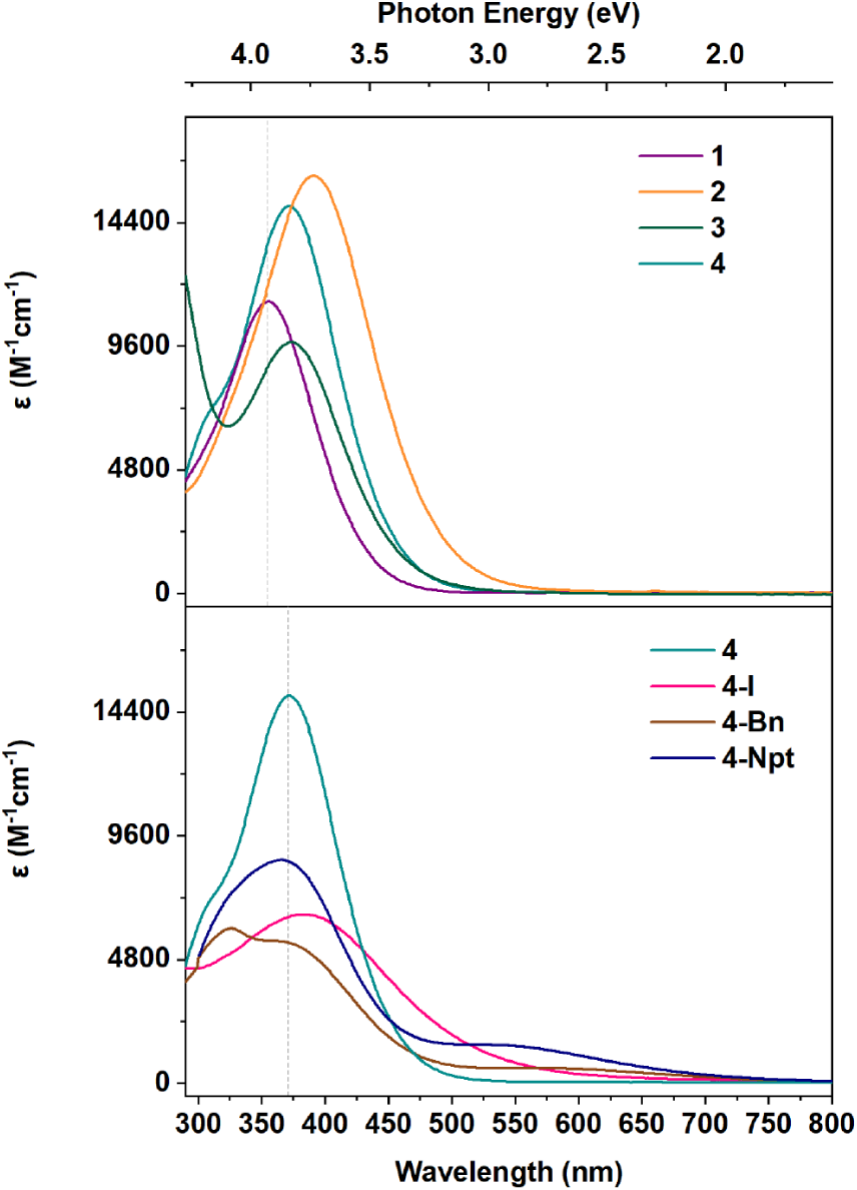
Experimental UV–vis spectra of Ce^4+^ complexes, measured in THF (**1**, **2**, **3**,**4**, and **4-I**), C_6_H_6_ (**4-Bn**) and Et_2_O (**4-Npt**). Dashed line is centered at λ_*max*_= 355 nm (**1**) and 371 nm (**4**) to show the shift across complexes.

The tetrahomoleptic complexes **1** - **4** all show a single LMCT feature varying between 355 nm (3.49 eV) (**1**) to 391 nm (3.17 eV)^13^ (**2**). **2** and **3** are both supported with N,N’-di-*tert*-butylethylenediamide ligands only differing by one substituent, NEt_2_ for **2** (NP*) and a phenyl group for **3** (NP^*,*Phen*^). This perturbation shifts the LMCT feature from 391 nm (**2**) to 373 nm (**3**), a change of 18 nm (0.15 eV). The **4**, **4-I**, **4-Bn**, and **4-Npt** series demonstrate the effect on the LMCT feature from substituting a single ligand while bearing a conserved geometry. All of these molecules are supported by the [NP­(^*t*^Bu)_3_]^−^ ligand.^47^ **4** is tetrahomoleptic whereas the other complexes feature three [NP­(^*t*^Bu)_3_]^−^ ligands with the fourth ligand as I^1–^, Npt^1–^, or Bn^1–^.^45^ We note that the shoulder feature of the UV–vis spectrum of **4** could be fitted (Figure S24, Table S5), which is in agreement with CASCISD results. Substituting [NP­(^*t*^Bu)_3_]^−^ with I^–^ red-shifts the highest energy LMCT feature by 11 nm (0.1 eV) from 371 (**4**) to 382 nm (**4-I**). In contrast, substitution with a Ce–C σ-bond blue-shifts the N to Ce LMCT charge transfer features to 326 nm (**4-Bn**) and 357 nm (**4-Npt**). The shift from **4** to **4-Bn** is 45 nm (0.46 eV) whereas the shift from **4** to **4-Npt** is 14 nm (0.13 eV). The heteroleptic series is noticeable in its variance of LMCT energies, as the largest shift is observed between **4-Bn** (lowest, 324 nm) and **4-I** (highest, 382 nm). Additionally, the introduction of a carbon donor introduces a second, lower energy LMCT (previously assigned computationally) at 602 nm (**4-Bn**) and 545 nm (**4-Npt**).^45^

### Electrochemistry

Cyclic voltammograms of **2**,^12^ **3**, **4**,^48^ **4-I**, **4-Bn**, and **4-Npt** ^45^ at 200 mV/s shown in Figure 3 demonstrate the ligand effect on the redox chemistry of the tetravalent Ce complexes. These complexes demonstrate quasi-reversible voltammograms wherein the rate of electron transfer is limited by the rate of mass transfer.^49^ Comparison of the E_*pc*_ values give insights into the thermodynamics of the Ce^4+/3+^ couple in the complexes studied.

**Figure 3:**
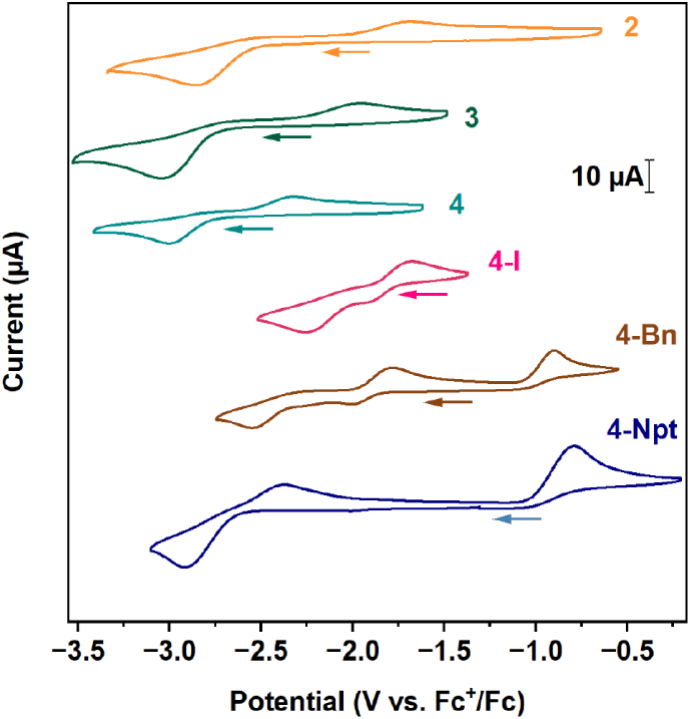
Cyclic voltammograms of Ce^4+^ imidophosphorane complexes at 200 mVs^–1^. Cyclic voltammogram of **3** was collected at 2.5 mM analyte and 0.1 M [(^*n*^Bu)_4_N]­[PF_6_]­in THF. Previously reported acquisition conditions are 2.5 mM analyte and 0.1 M [(^*n*^Bu)_4_N]­[PF_6_]­in THF (**2**), 1 mM analyte and 0.1 M [(^*n*^Bu)_4_N]­[BPh_4_] in THF (**4**), 3 mM analyte in 0.1 M [(^*n*^Bu)_4_N]­[BPh_4_] in THF (**4-I**), 3 mM in 0.2 M [(^*n*^Bu)_4_N]­[PF_6_] in PhF (**4-Bn**, **4-Npt**).

The comparison between **2** and **3** shows that the substitution of NEt_2_ to phenyl groups shifts the reduction event from *E*_*pc*_ = −2.86 V (**2**) to *E*_*pc*_ = −3.05 V (**3**), demonstrating a negative shift of 0.19 V. Replacement of one imidophosphorane ligand with a halide (I^–^) or an organic ligand (Bn^–^, Npt^–^) thermodynamically destabilizes the tetravalent state, as seen from the positive shift of the reduction event, E_*pc*_ among the [NP­(^*t*^Bu)_3_]^−^ supported complexes **4**, **4-I**, **4-Bn** and **4-Npt**.^45,48^ E_*pc*_ values of the complexes change over a range of 0.76 eV, with the most negative complex **4** being the most stabilized. Therefore, the trend follows **4** > **4-Npt** > **4-Bn** > **4-I**, with *E*_*pc*_ values of –3.01 V, –2.92 V, –2.55 V, and –2.25 V, respectively.^45,48^ The small secondary reductive features observed near −2 V in the voltammogram of **4-I** and **4-Bn** have previously been attributed to speciation or solution behavior.^45^ Cyclic voltammetry was not conducted for **1** as compatible electrochemical conditions could not be identified. However, chemical bracketing of the potential reveals that the *E*_*pc*_ of **1** is between –2.30 V and –2.47 V (in THF).^12^

### X-ray Scattering Studies

Given the ligand tunable electronic absorption features and electrochemical behavior, X-ray absorption spectroscopy at the Ce *L*_3_-edge was conducted. Transmission XANES was previously employed to assign oxidation states in previously published work for **1**,^12^ **2**,^13^ and **4**,^45^ and newly collected for **3**, **4-I**, **4-Bn**, and **4-Npt**. The *L*_3_-edge XANES transmission spectra across all of the complexes present in this work exhibit a double-peak feature with a broad pre-edge feature, corresponding to a tetravalent Ce oxidation state. In addition, the transmission Ce *L*_3_-edge XANES of the overlay of transmission XANES (Figure S25) shows only a small variance in the spectral shape and intensity among the complexes. This observation motivated further investigation of Ce *L*_3_-edge features, prompting the need for higher resolution measurements provided by high energy fluorescence detected (HERFD)-XANES.

HERFD-XANES of **1**, **2**, and **3** were collected at BL 15–2 ^50^ at Stanford Synchrotron Radiation Lightsource (SSRL), while the spectra of **4**, **4-I**, **4-Bn** and **4-Npt** were collected at BM20 ^51,52^ at European Synchrotron Radiation Facility (ESRF). HERFD-XANES were obtained by selecting the maximum of the emission line with a bandwidth of 1 eV. This choice ensures that the resulting spectra between the experiments are comparable. Spectra of all complexes in this study were collected at the maximum of the emission line, near the CeL_α1_ line. Details of the energy selection as well as individual fits of the spectra are shown in the Supporting Information. Figure 4 shows the HERFD-XANES Ce *L*_3_-edge spectra of the cerium complexes of interest. In comparison to the transmission data, the pre-edge feature at 5718 eV, is well-resolved with HERFD-XANES. Since the pre-edge feature arises from 2*p*_3/2_ → 4*f* transitions, peaks in this region can be used to identify the oxidation state.^16^ For reference, the tetravalent cerium in CeO_2_ exhibits a pre-edge feature at 5718.03(9) eV, similar to all complexes presented in this work except for **4-I**, though still fairly close at 5717.50(6) eV. There is another lower energy pre-edge peak emerging for **4-I** around 5715 eV (Figure S34). The presence of two peaks in the pre-edge region and the relatively red-shifted rising edge are attributed to the partial reduction of **4-I** from Ce^4+^ to Ce^3+^ in the X-ray beam.^16^ Further discussion of the **4-I** pre-edge is in the Supporting Information (Figure S34). Nevertheless, the position of the pre-edge peak supports the claim that the Ce in these complexes (other than **4-I**) is Ce^4+^, consistent with evidence provided from NMR, UV–vis, electrochemistry, and SC-XRD.^12,13,45,47^ Shared among all complexes in this work, the first main edge peak is split into two features at 5724 and 5728 eV, labeled *A* and *A*′, respectively (Table 1). All of these complexes also exhibit a higher energy single peak at 5735 eV, labeled *B*, contrary to the double-peak, labeled B/B’, observed in this region of the Ce *L*_3_-edge of CeO_2_ reported in previous work.^16^ The peak energies were notably unchanged between all of the complexes beyond the uncertainty of the fits, aside from **4-I**.

**Figure 4:**
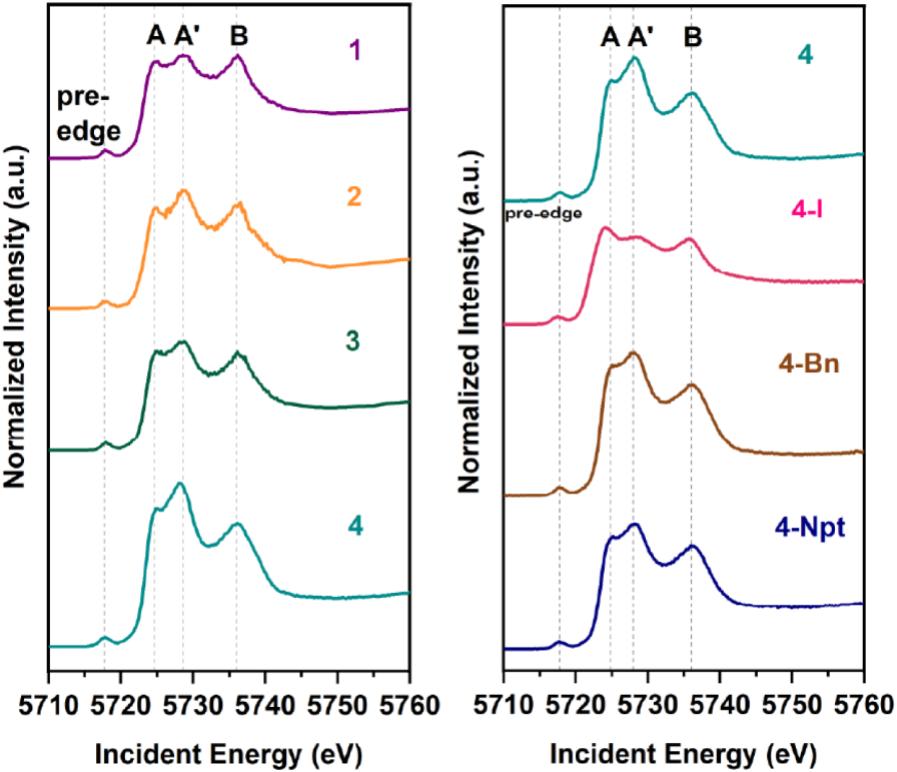
Experimental HERFD-XANES spectra of Ce^4+^ imidophosphorane complexes at the Ce L_3_-edge.

**Table 1:** Experimental Ce *L*_3_-Edge HERFD-XANES Peak Values Are from Figure 4

	pre-edge (eV)	A (eV)	A’ (eV)	B (eV)
**1**	5717.93(9)	5724.30(3)	5728.1(4)	5735.8(1)
**2**	5717.8(2)	5724.42(4)	5728.50(7)	5735.6(1)
**3**	5718.0(1)	5724.52(2)	5728.1(1)	5735.88(7)
**4**	5717.79(7)	5724.37(1)	5727.76(3)	5735.82(3)
**4-I**	5717.50(6)	5723.61(2)	5727.9(2)	5735.62(6)
**4-Bn**	5717.81(7)	5724.42(1)	5727.64(7)	5735.6(1)
**4-Npt**	5717.80(8)	5724.42(1)	5727.71(8)	5735.7(1)

### Resonant Inelastic X-ray Scattering (RIXS)

To visualize the HERFD-XANES spectra, core-to-core resonant inelastic X-ray scattering (RIXS) was employed on **4** and its previously reported^47^ Ce^3+^ analogue, [CsCe­(NP­(^*t*^Bu)_3_)_4_] (**4** ^**Cs**^) at ESRF BM20. In RIXS, the incident energy is scanned in the XANES region and the energy transfer between the ground state and the final state is measured as a function of energy. In this experiment, HERFD spectra of **4** was collected at 4840.3 eV energy at which A/A’ peak of **4** reaches its maximum. Figure 5 shows distinct RIXS profiles between tetravalent complex **4** and trivalent complex **4** ^**Cs**^. **4** has two fully resolvable features shown in red (Figure 5A) as well as a pre-edge peak shown as the inset in Figure 5A, while **4** ^**Cs**^ only displays a resonance corresponding to a single white line feature and a weak pre-edge resonance as expected for a trivalent complex (Figures 5B, S42).^53^

**Figure 5:**
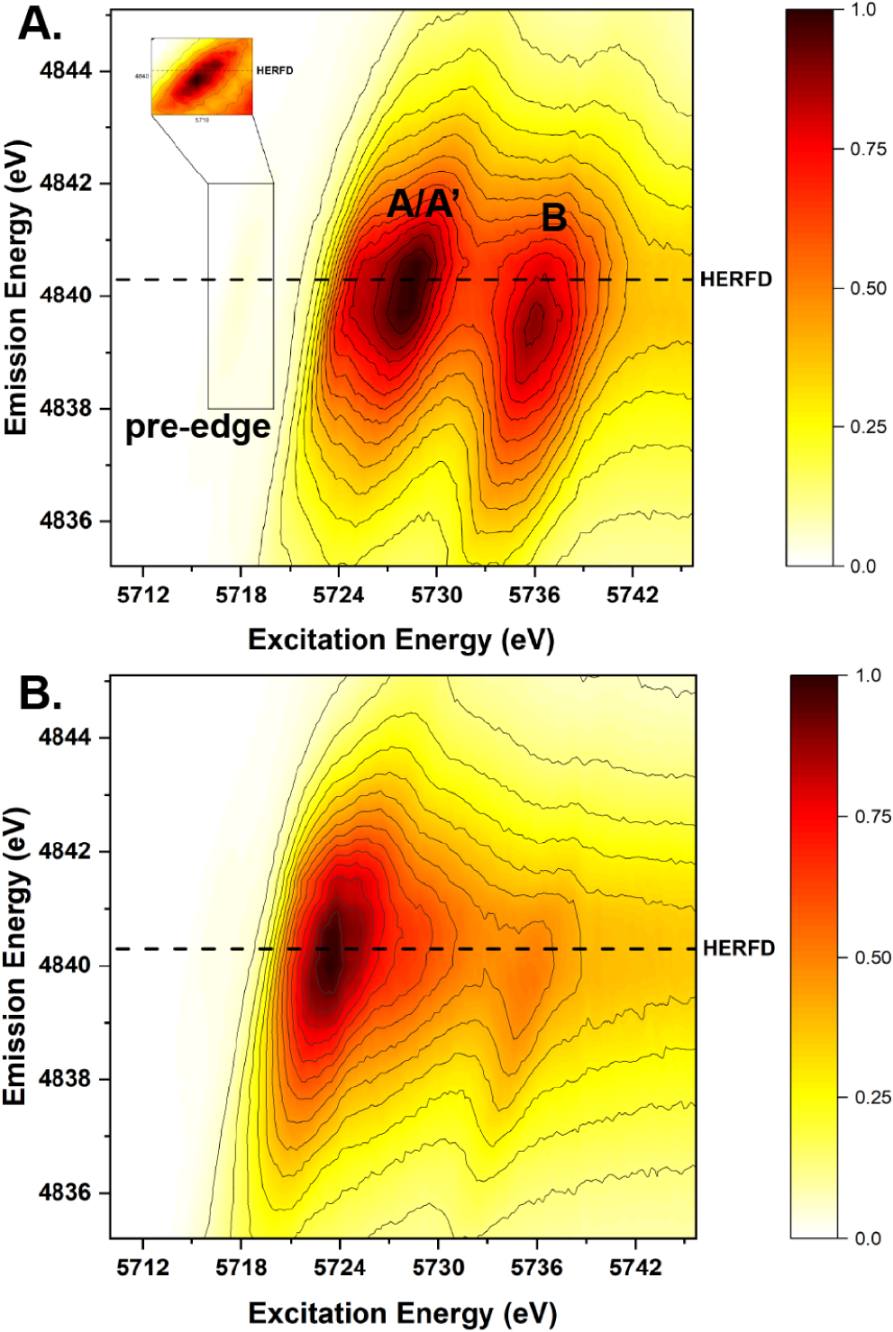
Experimental core-to-core RIXS map with emission energy (eV) plotted as a function of incident energy (eV) for **4**(A) and **4** ^**Cs**^(B). The dashed line indicates the energy of the maximum resonance at which the HERFD spectra were collected (4840.3 eV).

The observed HERFD spectrum is a “cut” of the RIXS map^54^ at the emission energy where the HERFD spectrum was collected. Intriguingly, in Figure 5A, the RIXS map of **4**, the maximum of A/A’ peak does not line up with the maximum of B peak. This observation is crucial to understanding the relative intensities of the peaks *A*, *A*′, and *B* of the HERFD spectra of **4** (Figure 4). Therefore, relative intensities of these peaks are highly dependent on the energy in which the HERFD spectrum was collected,^55^ and does not necessarily correlate with the maxima observed in the corresponding RIXS spectrum. A single feature with maximum intensity observed in the trivalent complex, **4** ^**Cs**^ is consistent with reported RIXS spectra of Ce_2_(CO_3_)_3_.^16,54^

### Computational Studies

Computational studies were undertaken to identify the physical underpinnings behind the differences in the UV–vis spectra and electrochemical behavior of this series of complexes. In the past, computational studies on Ce^4+^ complexes employed the complete active space self-consistent field (CASSCF) method to account for left–right correlation and multiconfigurational character, as previously demonstrated in these complexes.^37,38^ The accuracy of active space methods depends on whether the orbitals included in the active space sufficiently span the Hilbert subspace that the ground state wave function resides. Because these complexes exhibited heavily mixed SCF canonical orbitals, with Ce 4*f* and ligand orbital character dispersed across several tens of orbitals, to have an active space that thoroughly encompasses Ce 4*f* and coordinating ligand orbitals, the active space needed to be quite large. We believe that truncating the full CI expansion by excitation level would be less detrimental to the wave function quality than limiting the active space to a manageable size for CASSCF. For this reason, we employed complete active space configuration interaction with only single and double excitations (CASCISD) with the spin-free exact-two-component (X2C) Hamiltonian to probe the ground state of these complexes.^37,38^ An initial attempt used a cam-B3LYP Kohn–Sham orbital reference and an active space of all occupied orbitals with at least a Mulliken population of 0.07 on valence Ce *s*-atomic orbitals (AOs), valence Ce *s*-AOs, *p*-AOs from coordinating atoms, or *s*-AOs from coordinating atoms along with low-energy virtual orbitals with at least a Mulliken population of 0.07 on Ce *f*-AOs or Ce *s*-AOs. Computational and active space details for each system can be found in the Supporting Information. This calculation yielded ground states with slight multiconfigurational character for all seven complexes, with the ground state determinant contributing about 93% to the ground state CASCISD wave function.

The Kohn–Sham orbitals were transformed to a set of orbitals where the CASCISD ground state one-particle density matrix (1PDM) is diagonal, resulting in the natural orbital reference. In this reference, a set of 14 4*f*-orbitals were resolved, making the natural orbital reference better suited for studying the oxidation state of Ce. The eigenvalues of the 1PDM correspond to the occupation numbers of the natural orbitals. This analysis shows that these 4*f*-orbitals collectively have an occupation number of about 0.01 for all seven molecules.

By performing another CASCISD calculation with the natural orbital reference, the ground state wave function was almost entirely a single reference, with 99% contribution from a single determinant for all seven molecules. Moreover, the ground state energy was lower when using the natural orbital reference, indicating that this wave function more accurately represented the ground state. This shows that the choice of orbital reference is vital in determining whether Ce­(IV) complexes possess multiconfigurational ground states, as there may exist an orbital reference where the ground state wave function can be expressed as a single determinant.

Given that the ground state can be described by a single determinant and the absence of 4*f*-occupancy across all seven complexes, the oxidation state of the Ce center is determined to be +4 and not mixed-valent. This result is consistent with the HERFD-XANES pre-edge observations.

However, Ce orbitals are involved in bonding. The natural orbitals also consisted of a set of orbitals dominated by 2*p*-character from coordinating atoms, some of which exhibited hybridization with Ce orbitals and displayed bonding character. The number of Ce-hybridized orbitals, the extent of hybridization, and the character of Ce orbitals involved in hybridization all depend on the ligands. The degree of Ce 4*f*-hybridization remained low across all seven complexes, with Ce 4*f* Mulliken populations remaining less than 0.04 for each orbital. All complexes exhibited four orbitals with significant Ce 5*d*-hybridization, though the degree of hybridization varied substantially with the ligand (average Mulliken populations of 0.16 for **4-I** and 0.45 for **3**). These orbitals transform into the *e* irreducible representation when the molecular geometry is approximated as *T*_*d*_. As the complexes deviate from *T*_*d*_ symmetry, mixing between *t*_1_ ligand 2*p*-orbitals and Ce 5*d*-orbitals becomes allowed, further modulating the 5*d*-character of the other ligand 2*p*-dominant orbitals. Although the unoccupied 5*d* orbitals are not clearly resolved in either the Kohn–Sham or CASCISD natural orbitals, their hybridization with the ligand orbitals still reflects ligand field splitting. Mulliken populations for the ligand 2*p*-dominant occupied orbitals and Ce 4*f*-orbitals can be found in the Supporting Information.

Since the ground state wave functions of these Ce^4+^ complexes are mostly single reference, even with the cam-B3LYP Kohn–Sham orbital reference, linear response time-dependent DFT suffices in capturing excited state information and simulating UV–vis spectra. Figure 6 shows the simulated UV–vis spectra of the homoleptic Ce^4+^ complexes at the crystal structure geometry. Solvent effects were included by using a polarizable continuum model. The simulated spectra for all four homoleptic complexes reproduced the low-energy peak around 350 to 400 nm. TDDFT shows that Ce–N π → 4*f* LMCT excited states were mostly responsible for the low-energy peak. Ligand-to-ligand charge transfer excitations also contributed significantly to this feature in the spectrum of **4**. TDDFT shows this feature as a double peak instead of a single broad peak. We believe the double peak feature was due to symmetry breaking in the ligand orbitals from geometric deviations between ligands. Line broadening from the thermal accessibility of different geometric conformations in solution may have caused this feature to appear as a less resolved broad peak in experiment. A high-energy shoulder appears around 300 nm as the molecule becomes more symmetric (**1** < **2** < **4**). This may be the result of the double peak feature becoming resolved due to the reduced number of unique conformations in higher symmetry. **3** exhibits an additional peak around 300 nm, arising from charge transfer into the phenyl π-system from other ligand functional groups.

**Figure 6:**
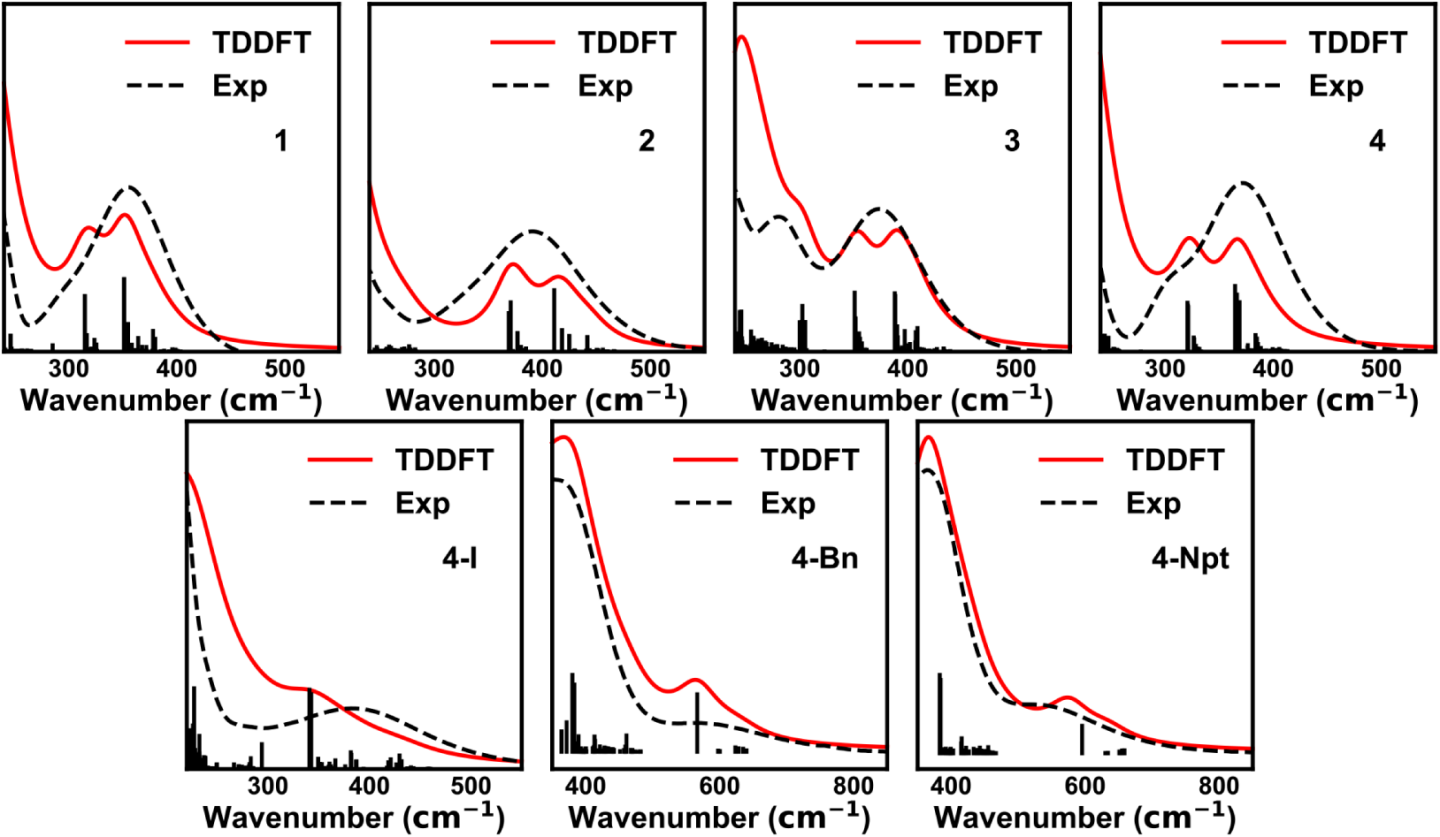
Simulated (red) and experimental (black dotted) UV-vis spectra for **1**, **2**, **3**, **4**, **4-I**, **4-Bn**, and **4-Npt**. The spectra were red-shifted 15, 28, 55, 28, 55, 75, and 25 nm, respectively. Excited states responsible for the spectra are plotted as black lines. Spectra for **1**, **2**, **3**, and **4** were generated by using Lorentzian broadening with a broadening parameter of 35 nm. Spectra for **4-I**, **4-Bn**, and **4-Npt** were generated by using Lorentzian broadening with a broadening parameter of 70 nm.

Figure 6 also presents the simulated UV–vis spectra of **4-Npt**, **4-Bn**, and **4-I** at crystal structure geometry. TDDFT was also able to reproduce the low-energy peak in these complexes. Previous work have shown that the low-energy peak of **4-Npt** and **4-Bn** mainly originate from Ce-Npt/Bn σ → 4*f* excitations.^45^ Following these states is a dense manifold of Ce–N π → 4*f* excited states, giving rise to the large peak at 400 nm. In contrast, the low-energy peak of **4-I** arises from Ce–N π → 4*f* excitations. As a result, the spectrum of **4-I** more closely resembles the spectra of the homoleptic complexes, which exhibit a low-energy peak around 400 nm, whereas **4-Npt** and **4-Bn** show corresponding features around 550–600 nm. Excited states arising from excitations out of orbitals involving I do not appear until <300 nm. The first 14 excited states of each complex are depicted as pairs of natural transition orbitals (NTOs) in the Supporting Information.

In all seven complexes, TDDFT shows that the lowest excited state is predominantly LMCT from Ce–N bonding orbitals, or Ce–C bonding orbitals for **4-Bn** and **4-Npt**, into Ce 4*f*. This places the bonding orbitals at the HOMO and the Ce 4*f*-orbitals at the LUMO. The lowest excitation energies fall around ∼ 2–3 eV. A HOMO–LUMO gap of this magnitude argues against a multiconfigurational ground state, consistent with the CASCISD results.

## Discussion

The spectroscopic studies of these complexes present potentially confounding evidence. On one hand, UV–vis spectra demonstrate that perturbations to the electronic and steric properties of the ligands are effective in tuning the 4*f* orbital energies relative to the ligand orbital energies. Substitution of imidophosphorane ligands shifts the LMCT energies from 326 to 391 nm (*n.b.* this is less than 1 eV). These ligand variations change the LMCT by two means. First, varying the ligands changes the ligand orbital energies, affecting the LMCT. Second, the change in ligand field perturbs the 4*f*-orbitals leading to a change in 4*f* orbital energies among the series of complexes, which also affects the LMCT energy. The latter is particularly evident in a comparison among **4**, **4-I**, **4-Bn**, and **4-Npt**. The imidophosphorane LMCT peak from the [NP­(^*t*^Bu)_3_]^−^ ligand to Ce^4+^ varies from 326 nm (**4-Bn**) to 382 nm (**4-I**) although the imidophosphorane ligand [NP­(^*t*^Bu)_3_]^−^ remains the same. This feature shows the effect of 4*f* energy tuning reflected in the LMCT by substituting one ligand with I^–^ and Bn^–^. Furthermore, these ligand variations modulate the redox chemistry of these complexes with *E*_*pc*_ varying over 0.5 V, demonstrating the tuning of relative 4*f* orbital energies.^56,57^

The further resolved *L*_3_-edge spectra obtained by HERFD-XANES reveal that the tuning of relative 4*f* orbital energies observed in the UV–vis spectra and electrochemical studies do not directly correspond to a change in the *L*_3_-edge HERFD-XANES. There are no systematic changes in the energy of the pre-edge, *A*, *A*′, and B peaks. This observation, taken together with the similar *n*_*f*_ values obtained by fitting of the transmission *L*_3_-edge XANES demonstrates that the *L*_3_-edge is not sensitive to these electronic features as would be expected if the complexes had multiconfigurational ground states. This insensitivity to ligand changes is therefore commensurate with the CASCISD results, which unequivocally reveal single-determinant ground state character for all of the Ce^4+^ imidophosohorane complexes in this study.

In the case of Ce^4+^ imidophosphorane complexes, the evidence from *L*_3_-edge transmission XANES, HERFD-XANES, and RIXS along with CASCISD supports the interpretation of double-peak feature as excited state in origin, arising from the multiconfigurational nature of the excited state, when the ground state is represented as single determinant, in line with the proposed interpretation of the double peak observed in CeO_2_ by Krill.^58^ This conclusion is also in agreement with multireference calculations on formally tetravalent cerium systems such as CeO_2_ ^36^ as well as [Ce­(BIPMTMS)­(ODipp)_2_]­(BIPM^*TMS*^ = C­(PPh_2_NSiMe_3_)_2_; Dipp = C_6_H_3_-2,6-^*i*^Pr_2_),^15^ which also demonstrate the double-peak feature originates from multiconfigurational excited state with increased donation bonding taken into account — a key feature in tetravalent lanthanides as the oxidation state increases.^17^

The perturbation in charge transfer properties coupled with the single-reference nature of the ground state paints a picture of these Ce^4+^ imidophosphorane complexes as a charge transfer insulator.^59^ The invariance across *L*_3_-edge XANES spectra overall resembles other classes of formally tetravalent Ce molecules, such as Ce­(acac)_4_, Ce­(trop)_4_, and Ce­(tmataa)_2_,^10^ Ce-imido and Ce-oxo complexes,^11^ and homoleptic complexes, [Ce­(L_*tBu*_)_2_], [Ce­(L_*H*_)_2_],[Ce­(L_*NO*2_)_2_] [where H_2_L_*tBu*_ = bis­(2-hydroxy-3,5-di-*tert*-butylbenzyl)­(2-pyridylmethyl)­amine, H_2_L_*H*_ = bis­(2-hydroxybenzyl)­(2-pyridylmethyl)­amine and H_2_L_*NO*2_ = bis­(2-hydroxy-5-nitrobenzyl)­(2-pyridylmethyl)­amine],^14^ and CeX_6_^2–^ (X = F, Cl, Br) complexes.^39,40^

Although the ligand identities do not change the HERFD-XANES spectra within the imidophosphorane series, the higher energy feature *B* was different from that of CeO_2_. The higher energy peak of CeO_2_ resolved into two peaks B/B′ at 5735.9(1) eV and 5739.55(5) eV. A potential explanation is that the A/A′ and B/B′ splitting is due to crystal field splitting. The pseudotetrahedral complexes experience significantly weaker crystal field splitting than the 8-coordinate cubic CeO_2_, with a decrease in splitting of more than 3.65 eV. Thus, B/B’ peaks in **4** cannot be fully resolved with confidence using HERFD-XANES and the within the limitations of the fitting models employed (fitting parameters including fit width can be found in SI, Table S24). This explanation requires further support, potentially from valence-to-core *L*_3_-edge RIXS studies.^21,60,61^ The relative intensities of the A and B features observed in RIXS studies confirm the previously reported observation that HERFD-XANES relative intensities are highly sensitive to the emission energy at which HERFD-XANES spectra are collected.^55^ Lastly, the RIXS map of **4** shows that the A peak and B peak do not line up at the maximum resonance of the A peak at 4840.3 eV, and the origin of this phenomenon is a subject of future study.

## Conclusions

We have prepared a series of Ce^4+^ molecular complexes in a conserved coordination motif in order to examine the ligand effects on the modulation of the ground state electronic configuration. While electronic absorption spectra in the UV–vis region and electrochemical analyses clearly demonstrate a perturbation of the relative 4*f* orbital energies, CASCISD and HERFD-XANES show that the ground state of these complexes remain single-reference with a Ce^4+^ center. The agreement between HERFD-XANES and CASCISD results demonstrates that the ground state of the Ce^4+^ imidophosphorane complexes can be simply described as single-reference and implicates the origin of the double peak feature to the excited state multiconfigurational character in these tetravalent cerium complexes. However, the library of high valent lanthanide complexes are expanding to include complexes such as Pr^5+^ in [Pr­(NP^*t*^Bu_3_)_4_]^+^, where complete active space self-consistent field (CASSCF) calculations indicate a highly multiconfigurational electronic structure with significant LMCT to the Pr metal center,^48^ and therefore suggest that multiconfigurational ground state behavior may be found beyond cerocene and its related organometallic derivatives. Therefore, delineation of the physical origin of multipeak features at the lanthanide *L*_3_-edge is critical to facilitate the understanding of electronic structures of the expanding class of molecular high-valent lanthanide complexes.

## Supplementary Material



## Notes

Additional experimental considerations, NMR, IR, UV–vis-NIR, electrochemical analyses, XANES and HERFD-XANES fitting, computational and crystallographic information, crystallographic data of **3** is deposited in Cambridge Crystallographic Data Centre (CCDC) as deposition number 2453410; Normalized HERFD-XANES spectra of all complexes and normalized *L*_3_-edge transmission XANES data (PDF)
