# Supplementary material for: Single-Determinant Ground State in Ce4+ Imidophosphorane Complexes
Source: Inorg Chem. 2025 Dec 11;64(50):24401–10. doi: 10.1021/acs.inorgchem.5c03376 (PMC12728922; doi:10.1021/acs.inorgchem.5c03376)
Supplement: Supplementary file 1 [file ic5c03376_si_001.pdf]

Supporting Information:

Single-Determinant Ground State in Ce^{4+} Imidophosphorane Complexes

Haruko Tateyama[†], Can Liao[‡], Grant R. Wilkinson[†], Arun Ramanathan[†], Lucia Amidani[¶,§], Elena Bazarkina[¶,§], Florian Ressnik[†], Kaitlyn S. Engle[†], John Bacsa[†], Dimosthenis Sokaras[||], Kristina O. Kvashnina[¶,§], Xiaosong Li[‡] and Henry S. La Pierre*[,†,⊥]

[†]School of Chemistry and Biochemistry, Georgia Institute of Technology, Atlanta, Georgia 30332-0400, United States

[‡]Department of Chemistry, University of Washington, Seattle, Washington 98195, United States

[¶]The Rossendorf Beamline at ESRF, The European Synchrotron, 38043 Grenoble Cedex 9, France

[§]Institute of Resource Ecology, Helmholtz Zentrum Dresden Rossendorf (HZDR), 01314 Dresden, Germany

[||]Stanford Synchrotron Radiation Lightsource, SLAC National Accelerator Laboratory, Menlo Park, California 94025, United States.

[⊥]Nuclear and Radiological Engineering and Medical Physics Program, School of Mechanical Engineering, Georgia Institute of Technology, Atlanta, Georgia 30332-0400, United States

E-mail: hsl@gatech.edu

Table of Contents

General Considerations 2
NMR Spectroscopy 3
Crystallographic analyses 9
Comparison of Bond Metrics of Ce^{4+} Imidophosphorane Complexes 12
Computational Details 13
Electrochemistry 31
UV-vis NIR Electronic Absorption Spectra 32
Ce L_3-edge X-ray Absorption Transmission Near Edge Spectroscopy (XANES) 34
Ce L_3-edge High Energy Resolution Fluorescence Detection (HERFD)-XANES and Resonant Inelastic X-ray Scattering(RIXS) 43
Discussion on the partial reduction observed in the HERFD-XANES spectrum of 4-I: 45
References 52

General Considerations

Synthesis: Synthesis and manipulation of air-sensitive ligands and metal complexes were conducted employing Schlenk techniques in an Ar Schlenk line or in an inert N_2 glovebox (Vigor) under <0.1 ppm O_2/H_2O. All glassware and cannulas were dried at 160°C for at least 12 hours prior to use. Celite and molecular sieves (3, 4 Å) were heated at >250 °C under vacuum for >48h. Diethyl ether, toluene, hexanes, pentane were degassed with inert Ar gas (Ultra High Purity, Airgas) and passed though Q-5 and molecular sieve column in a solvent purification system (JC Meyer Solvent Systems). All solvents used inside of the glovebox were stored over dried 4Å molecular sieves. Methanol (VWR) was dried over elemental Mg using catalytic I_2 over 12h, then distilled and stored over 3 Å molecular sieves. Dichlorophenylphosphine (Thermo Scientific Chemicals) and triethylamine (Fisher Scientific) were distilled prior to use. N,N-Di-tert-butylethylenediamine (TCI Chemicals) was used as received. NMR solvents were purchased from Cambridge Isotope Laboratory. d_6-benzene and d_8-THF were pre-dried over 3 Å molecular sieves (C_6D_6), degassed through freeze-pump-thaw, then dried over Na°/benzophenone and vac-transferred, then stored over 3 Å molecular sieves until use.d_8-toluene was degassed through freeze-pump-thaw, then stored over 3 Å molecular sieves until use. $CeI_3(THF)_4$[1] and benzyl potassium (KBn)[2] were prepared by previously reported procedures.

Analytical: NMR spectra were obtained on a Bruker Avance III 400 MHZ or 500 MHz spectrometer at 298K. ^1H NMR spectra and $^{13}C\{^1H\}$ NMR spectra are referenced to residual ^1H resonances of the deuterated solvent. $^{31}P\{^1H\}$ NMR was referenced using an absolute reference to H_3PO_4. Peak position is reported followed by peak multiplicity, integration value, and assignment where applicable. Abbreviations for the peak multiplicity are as follows: s (singlet); d (doublet); t (triplet); m (multiplet). Infrared (IR) spectroscopy was conducted on a Bruker ALPHA FTIR Spectrometer from 400 to 4000cm^{-1}, using an ATR attachment inside of a N_2 glovebox. The intensities of the peaks are reported using the following abbreviations: vw (very weak); medium (medium); s (strong); vs (very strong); br (broad). UV-vis NIR spectroscopy was conducted on a Hitachi UH4150 UV-vis-NIR scanning spectrophotometer from 1000 nm to 250 nm. Characterization was performed in small-volume screw cap quartz cuvettes (Starna Scientific) with a 1cm path length. Elemental analyses on C, H, N were performed at the University of Berkeley Microanalytical Facility (Berkeley, CA). Electrochemical measurements were conducted with a Pine WaveDriver20 Bipotentiostat/Galvanostat. Measurement was performed in a glovebox in N_2 under ambient temperature (~ 25 °C, 298 K). The electrochemical cell was set up with a glassy carbon working electrode (3 mm diameter), a bare Ag/AgCl wire reference electrode, and a platinum wire counter electrode in Pine Research 20 mL glass electrochemical cell. The electrolyte, $[^nBu_4N][PF_6]$ (Oakwood) was recrystallized three times from absolute ethanol them dried under vacuum at 100°C prior to use, and the electrolyte solution was prepared in THF as 0.1M $[^nBu_4N][PF_6]$. Ferrocene was sublimed prior to use. Measurements were made in positive feedback iR compensation mode (~450 Ω). At the end of the measurement, a small amount of ferrocene was added to the electrochemical cell as reference. All potentials are reported vs Fc^+/Fc. SC-XRD was performed at Georgia Institute of Technology X-ray Crystallography Facility on a Bruker D8 Venture diffractometer.

NMR Spectroscopy

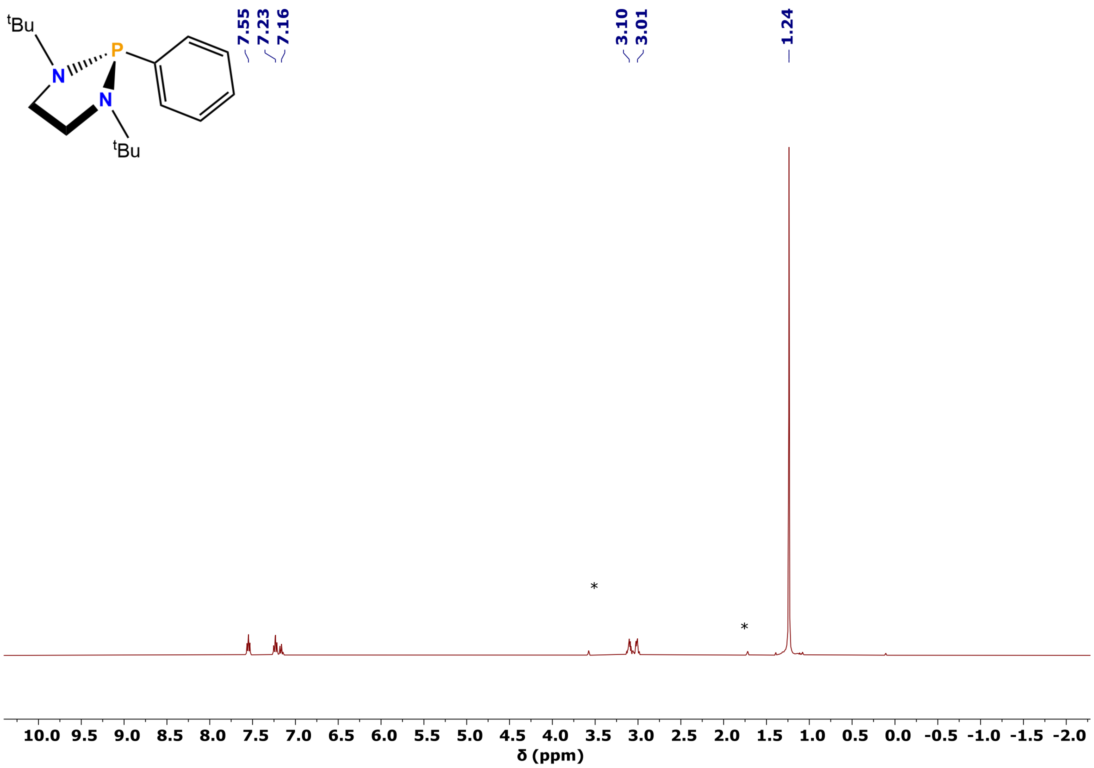

Figure S1. ^{1}H NMR of **5a** in d_8-THF. Residual solvent(d_8-THF) is denoted as *.

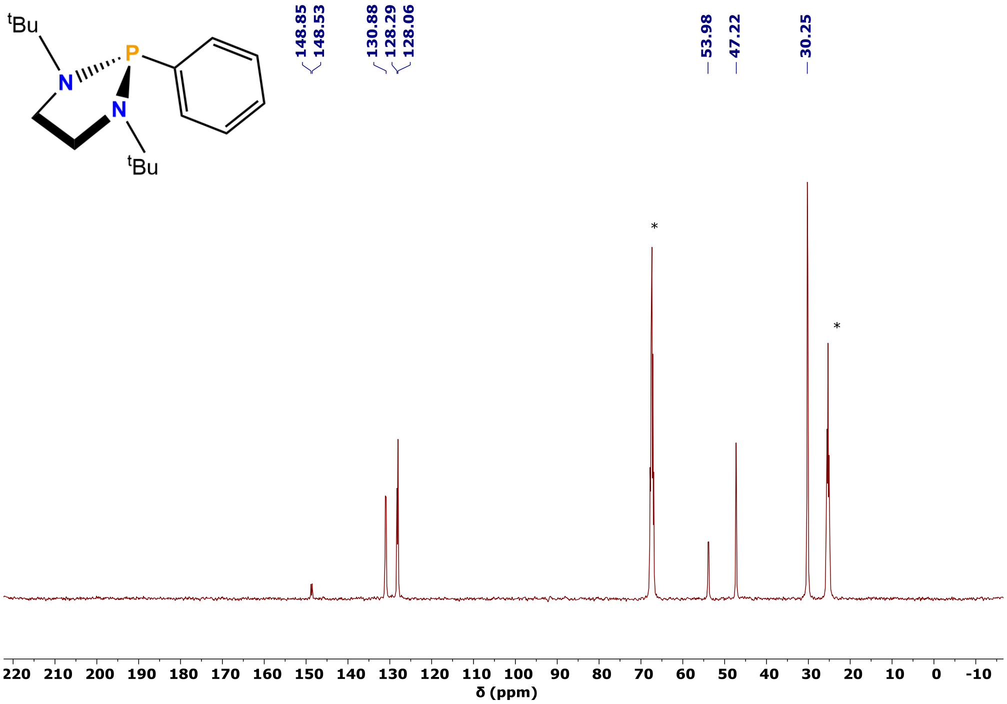

Figure S2. $^{13}C\{^{1}H\}$ NMR of **5a** in d_8-THF**.** Residual solvent(d_8-THF) is denoted as *.

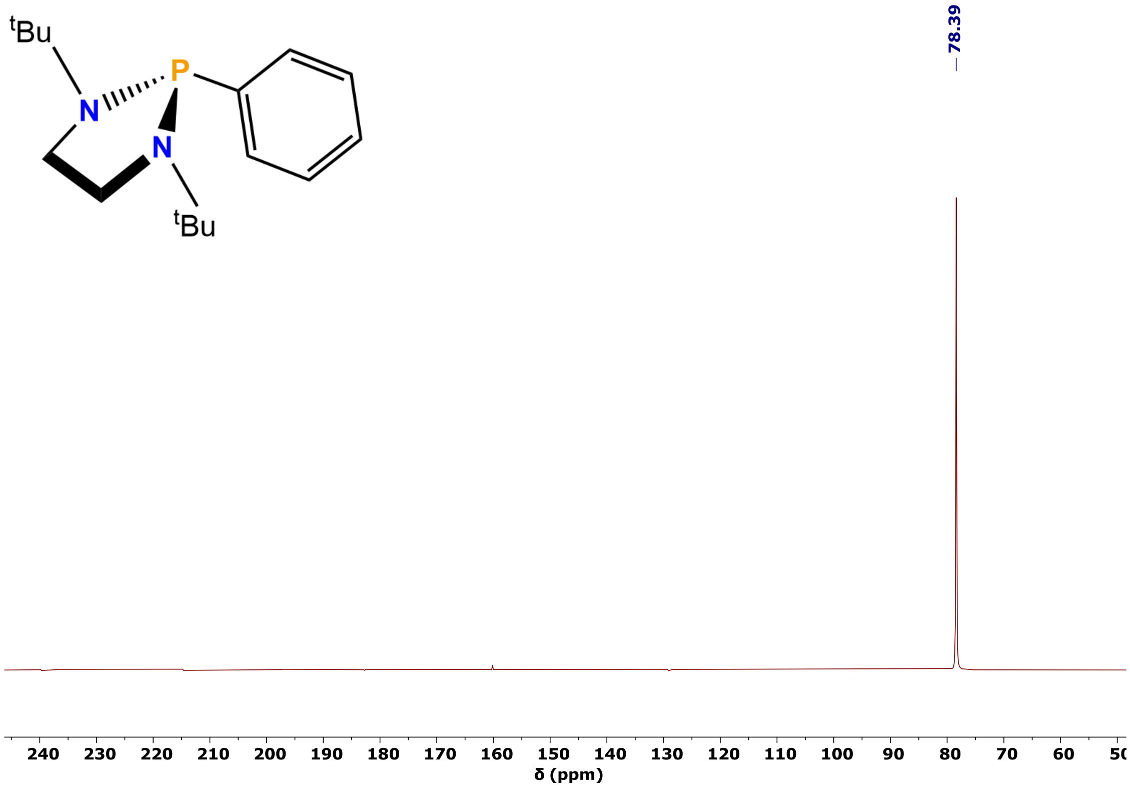

Figure S3. $^{31}P\{^{1}H\}$ NMR(162Hz) of **5a** in d_8-THF.

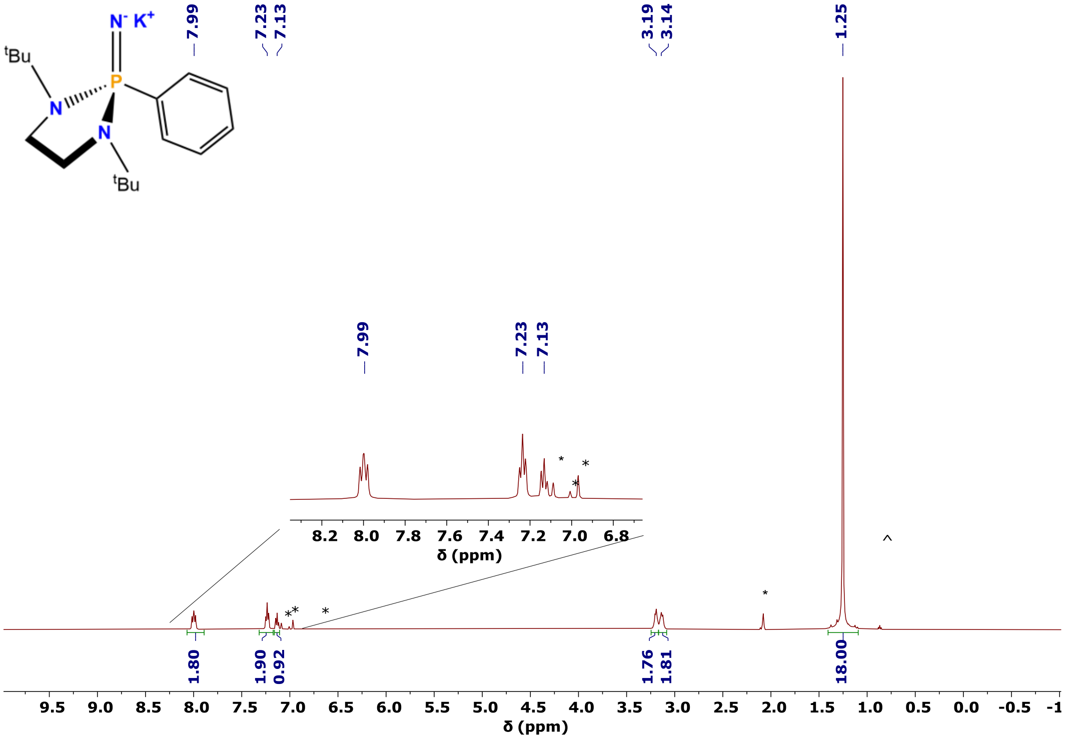

Figure S4. ^{1}H NMR of **5b** in d_8-tol. Residual solvent (d_8-tol) is denoted as *, and residual pentane is shown as ^.

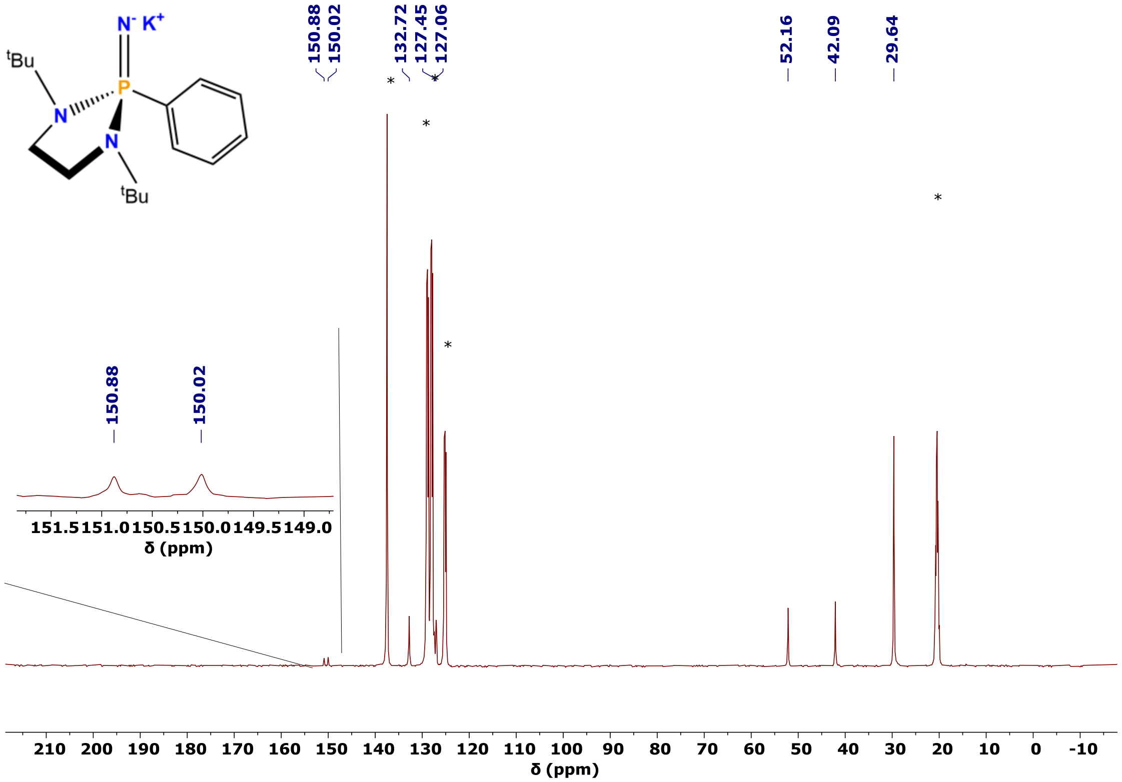

Figure S5. $^{13}C\{^{1}H\}$ NMR of **5b** in d_8-tol. Residual solvent is denoted as *.

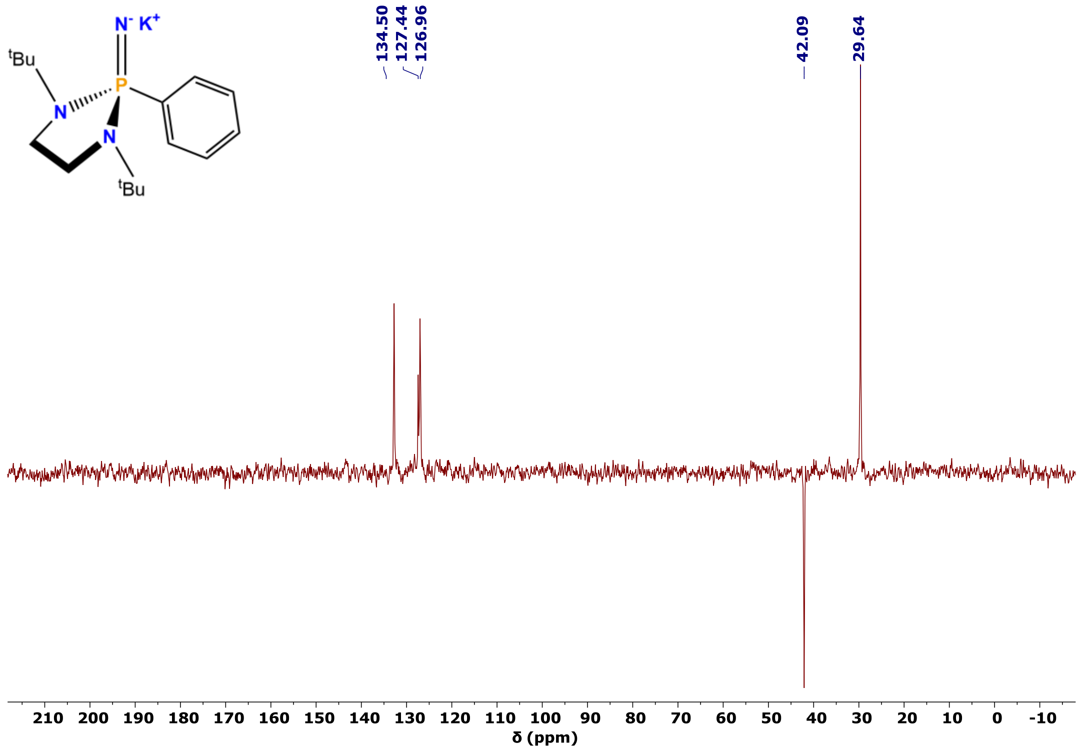

Figure S6. $^{13}C\{^{1}H\}$ DEPT 135-NMR of **5b** in d_8-tol. The spectrum is referenced to the $-CH_3$ peak at 29.64 ppm.

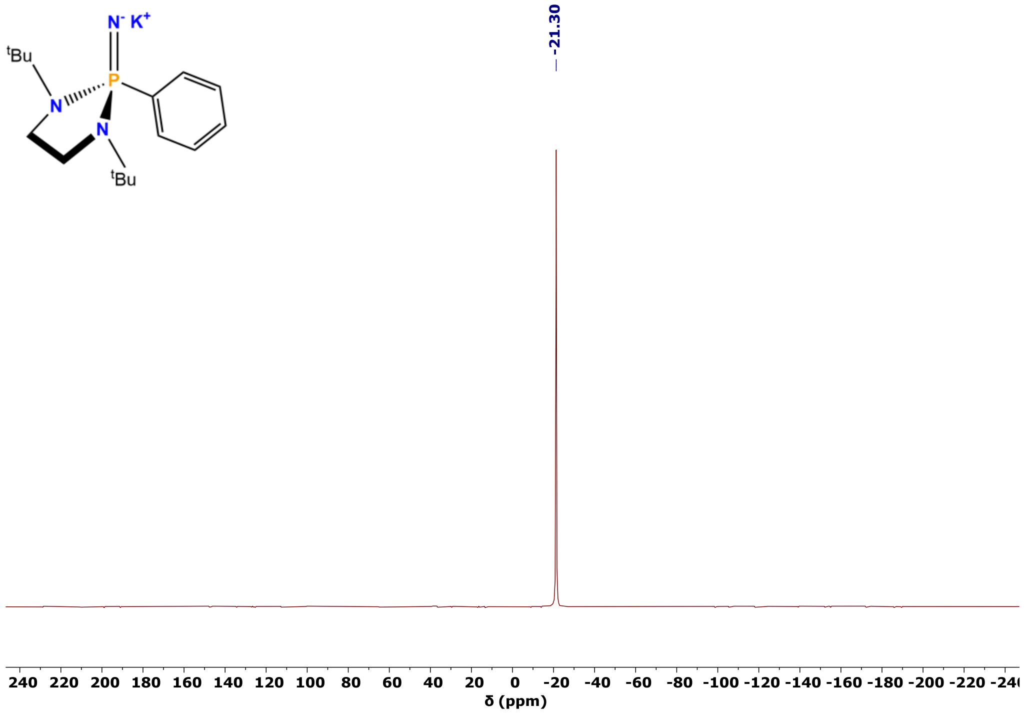

Figure S7. $^{31}P\{^{1}H\}$ NMR (202Hz) of **5b** in d_8-tol.

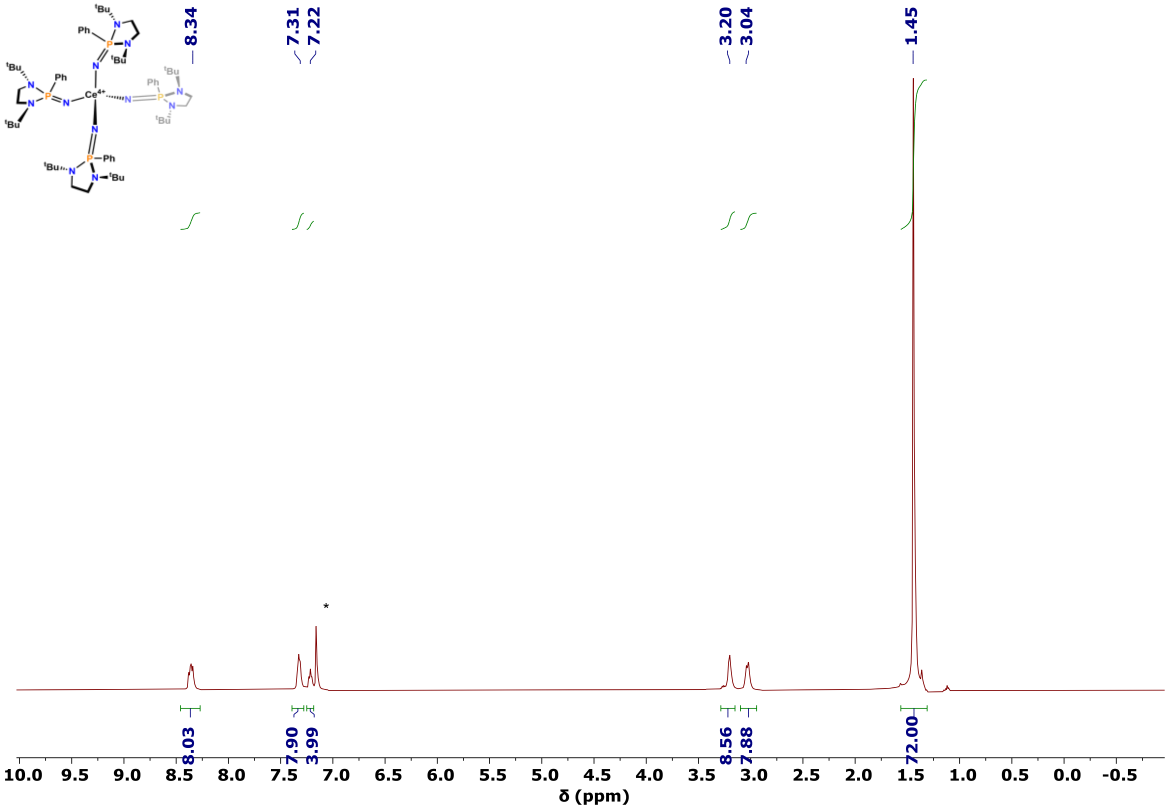

Figure S8. ^{1}H NMR of **3** in d_6-benzene. Residual solvent is denoted as *, residual Et_2O is shown as ^.

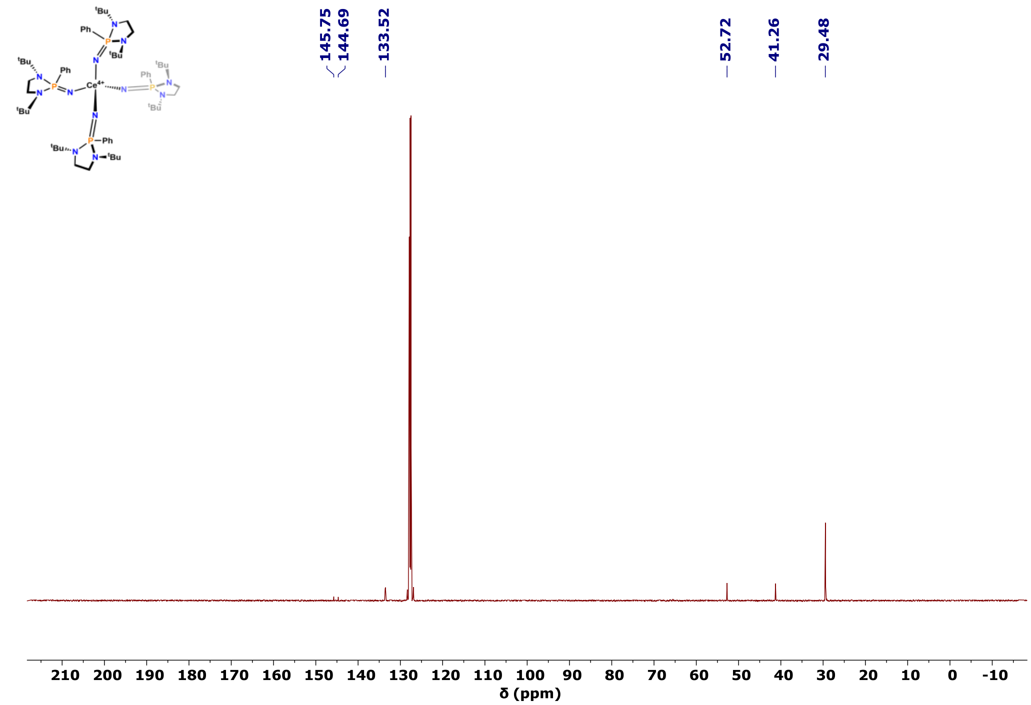

Figure S9. $^{13}C\{^{1}H\}$ NMR of 3 in d_6-benzene. Residual solvent (C_6D_5H) is denoted as *.

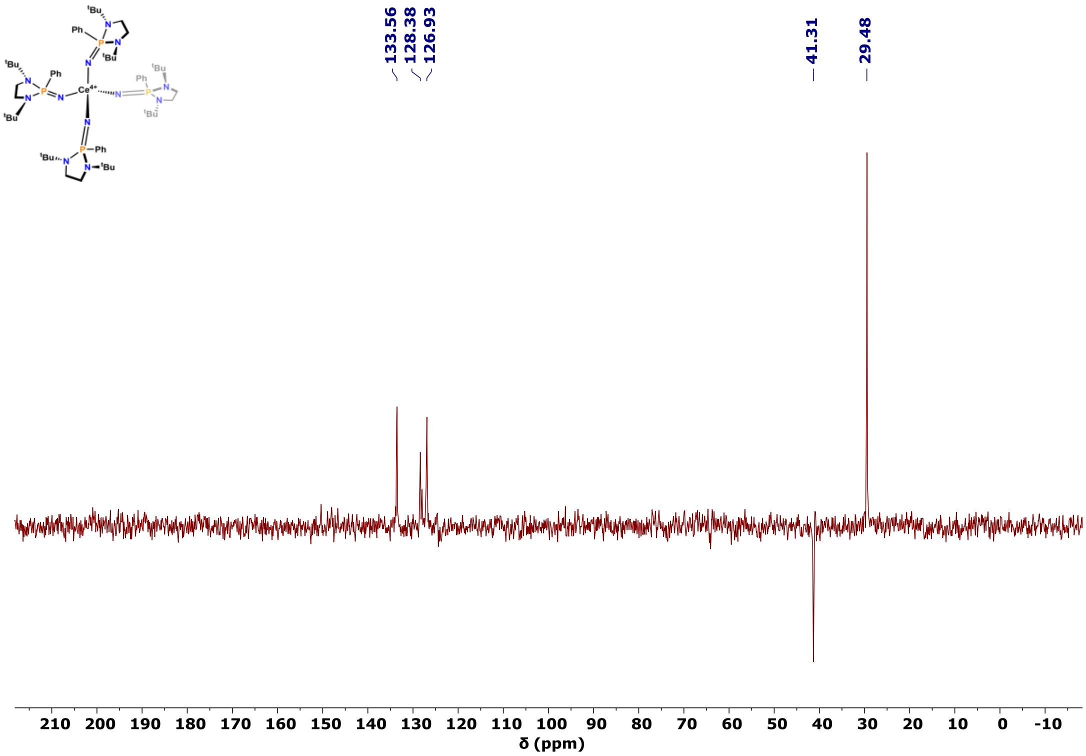

Figure S10. $^{13}C\{^{1}H\}$ DEPT 135-NMR of **3** in d_6-bwnzene. The spectrum is referenced to the $-CH_3$ peak at 29.48 ppm.

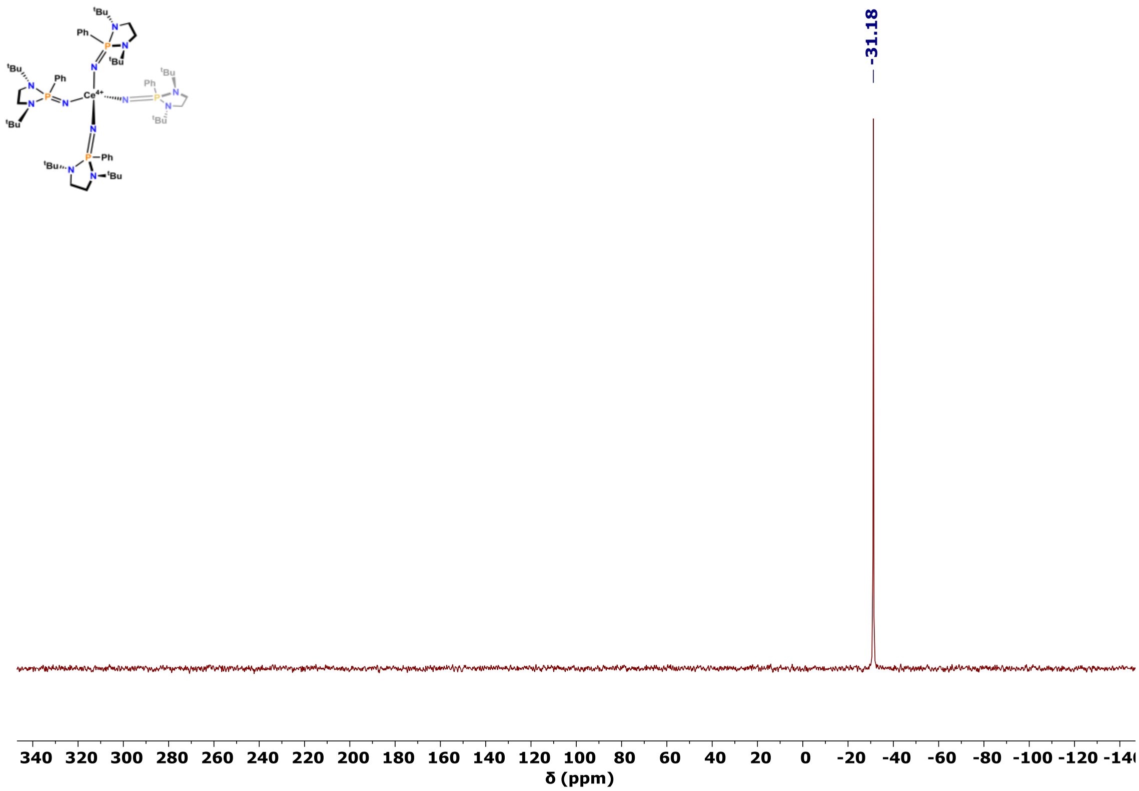

Figure S11. $^{31}P\{^1H\}$ NMR (162Hz) of **3** in d_6-benzene**.**

Crystallographic analyses

Experimental: Single yellow prism-shaped crystals of HT-B072 were chosen from the sample as supplied. A suitable crystal with dimensions 0.41 × 0.41 × 0.31 mm^3 was selected, coated with NVH(Cargille) oil, and mounted on a loop on a Bruker D8 VENTURE diffractometer. The crystal was kept at a steady *T* = 100(2) K during data collection. The structure was solved with ShelXT[3] and by using Olex2 1.5-alpha[4]. The model was refined with ShelXL-2014[5] using full matrix least squares minimisation on $\boldsymbol{F^2}$. Data were measured using f and w scans with MoKa radiation. The diffraction pattern was indexed and the total number of runs and images was based on the strategy calculation from the program APEX2 v2016.5-1.[6] The maximum resolution that was achieved was *Q* = 29.575° (0.72 Å). The unit cell was refined using SAINT V8.40B[7] on 9353 reflections.

Solution and Refinement Details. Data reduction, scaling and absorption corrections were performed using SAINT V8.40B[7]. The final completeness is 99.50 % out to 29.575° in *Q*. SADABS-2016/2[8] was used for absorption correction. wR_2(int) was 0.0927 before and 0.0747 after correction. The Ratio of minimum to maximum transmission is 0.7524. The *l*/2 correction factor is Not present. The absorption coefficient *m* of this material is 0.694 mm^{-1} at this wavelength (*l* = 0.71073Å) and the minimum and maximum transmissions are 0.562 and 0.747.

The structure was solved, and the space group $P2_1/c$ (# 14) determined by ShelXT[3] and refined by full matrix least squares minimisation on $\boldsymbol{F^2}$ using version 2018/3 of ShelXL-2014.[5] All non-hydrogen atoms were refined anisotropically. Hydrogen atom positions were calculated geometrically and refined using the riding model.

There is a single formula unit in the asymmetric unit, which is represented by the reported sum formula. In other words: Z is 4 and Z' is 1. The compound crystallized with two diethyl ether molecules. The moiety formula is $C_{64}H_{108}CeN_{12}P_4$, $2(C_4H_{10}O)$.

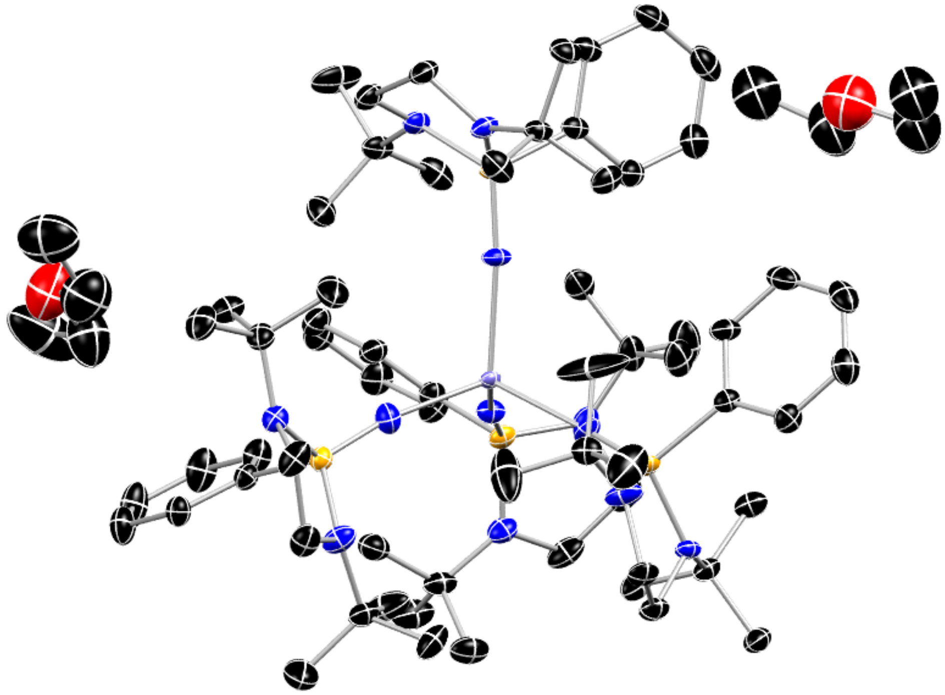

Figure S12. Molecular Structure of **3** with thermal ellipsoids shown at 50% probability. H atoms are omitted for clarity. Ce shown in purple, C shown in black, N shown in blue, P shown in orange and O shown in red. Disorder at one of the ^{t}Bu group and one of the Et_2O molecule as well as H atoms are omitted for clarity.

Table S1. Single-crystal X-ray Crystallography Data

	**3**
Empirical Formula	$C_{72}H_{128}CeN_{12}O_2P_4$ (Sum)<br>Moiety formula: $C_{64}H_{108}Ce$<br>$N_{12}P_4$,2($C_4H_{10}O$)
$D_{calc.}$/ g cm^{-3}	1.205
m/mm^{-1}	0.694
Formula Weight	1457.86
Colour	yellow
Shape	prism-shaped
Size/mm^3	0.41×0.41×0.31
T/K	100(2)
Crystal System	monoclinic
Space Group	$P2_1/c$
a/Å	22.8193(14)
b/Å	13.4451(8)
c/Å	27.0111(17)
a/°	90
b/°	104.085(2)
g/°	90
V/Å^3	8038.1(9)
Z	4
Z'	1
Wavelength/Å	0.71073
Radiation type	MoK_a
Q_{min}/°	2.023
Q_{max}/°	29.575
Measured Refl's.	116668
Indep't Refl's	22487
Refl's I≥2 *s*(I)	20183
R_{int}	0.0625
Parameters	861
Restraints	327
Largest Peak	1.665
Deepest Hole	-1.934
GooF	1.074
wR_2 (all data)	0.1313
wR_2	0.1265
R_1 (all data)	0.0574
R_1	0.0512
CCDC Number	2453410

Table S2. Selected bond length for **3.**

Atom	Atom	Length/Å
Ce1	N1	2.179(2)
Ce1	N2	2.178(2)
Ce1	N3	2.163(3)
Ce1	N4	2.166(2)
P1	N1	1.540(2)
P2	N2	1.538(2)
P3	N3	1.540(3)
P4	N4	1.540(2)

Table S3. Selected bond angles for **3.**

Atom	Atom	Atom	Angle/°
N2	Ce1	N1	109.95(9)
N3	Ce1	N1	110.91(10)
N3	Ce1	N2	108.43(10)
N3	Ce1	N4	109.31(10)
N4	Ce1	N1	109.05(10)
N4	Ce1	N2	109.16(9)
P1	N1	Ce1	172.22(16)
P2	N2	Ce1	173.56(15)
P3	N3	Ce1	170.33(18)
P4	N4	Ce1	170.46(18)

Comparison of Bond Metrics of Ce^{4+} Imidophosphorane Complexes

Table S4. Tabulated average Ce-N distance among Ce^{4+} imidophosphorane complexes from SC-XRD.

Complex	$Ce\text{-}N_{avg}$ (Å)	Reference
**1**	2.20(2)	Ref [9]
**2**	2.237(2)	Ref [10]
**3**	2.172(3)	New report
**4**	2.176(4)	Ref [11]
**4-I**	2.11(2)	Ref [12]
**4-Bn**	2.142(7)	
**4-Npt**	2.147(6)	

Computational Details

All calculations were performed using a developmental version (revision J26p) of the Gaussian electronic structure package. The x2c-TZVPall-2c basis set was used for Ce and I atoms.[13] The Pople 6-311+G basis set was used for C, P, and N atoms.[14–17] The STO-3G basis set was used for H atoms.[18]

Table S5. Active spaces for CASCISD calculations.

	Electrons	Orbitals
**1**	88	106
**2**	112	126
**3**	74	88
**4**	72	100
**4-I**	62	78
**4-Bn**	72	92
**4-Npt**	70	90

Table S6. **T**he largest determinant contribution (LDC) to the ground state wave function and ground state energies from CASCISD calculations using Kohn-Sham orbitals and natural orbitals.

	Kohn-Sham Orbitals		Natural Orbitals	
	Energy (Hartree)	LDC	Energy (Hartree)	LDC
**1**	-13431.5228889131	93%	-13431.5245085671	99%
**2**	-13285.0031323979	92%	-13285.0053075438	99%
**3**	-13358.7288149872	94%	-13358.7303343301	99%
**4**	-12317.0810140488	94%	-12317.0834309009	99%
**4-I**	-18563.2068846332	93%	-18563.2098048784	99%
**4-Bn**	-11720.5053078538	93%	-11720.5080590339	99%
**4-Npt**	-11647.0403583112	93%	-11647.0425763854	99%

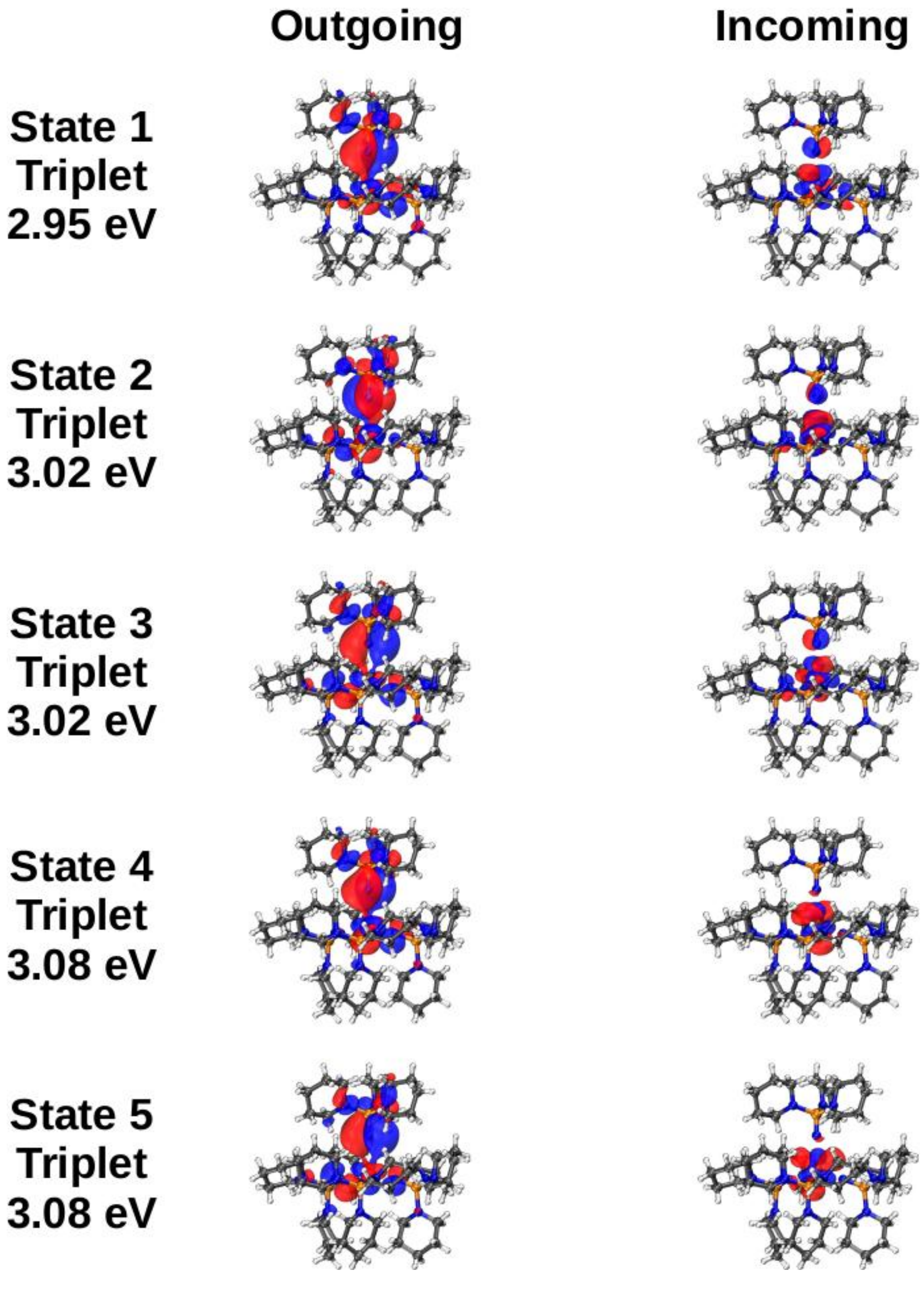

Figure S13. Natural transition orbitals of the first five excited states for **1**.

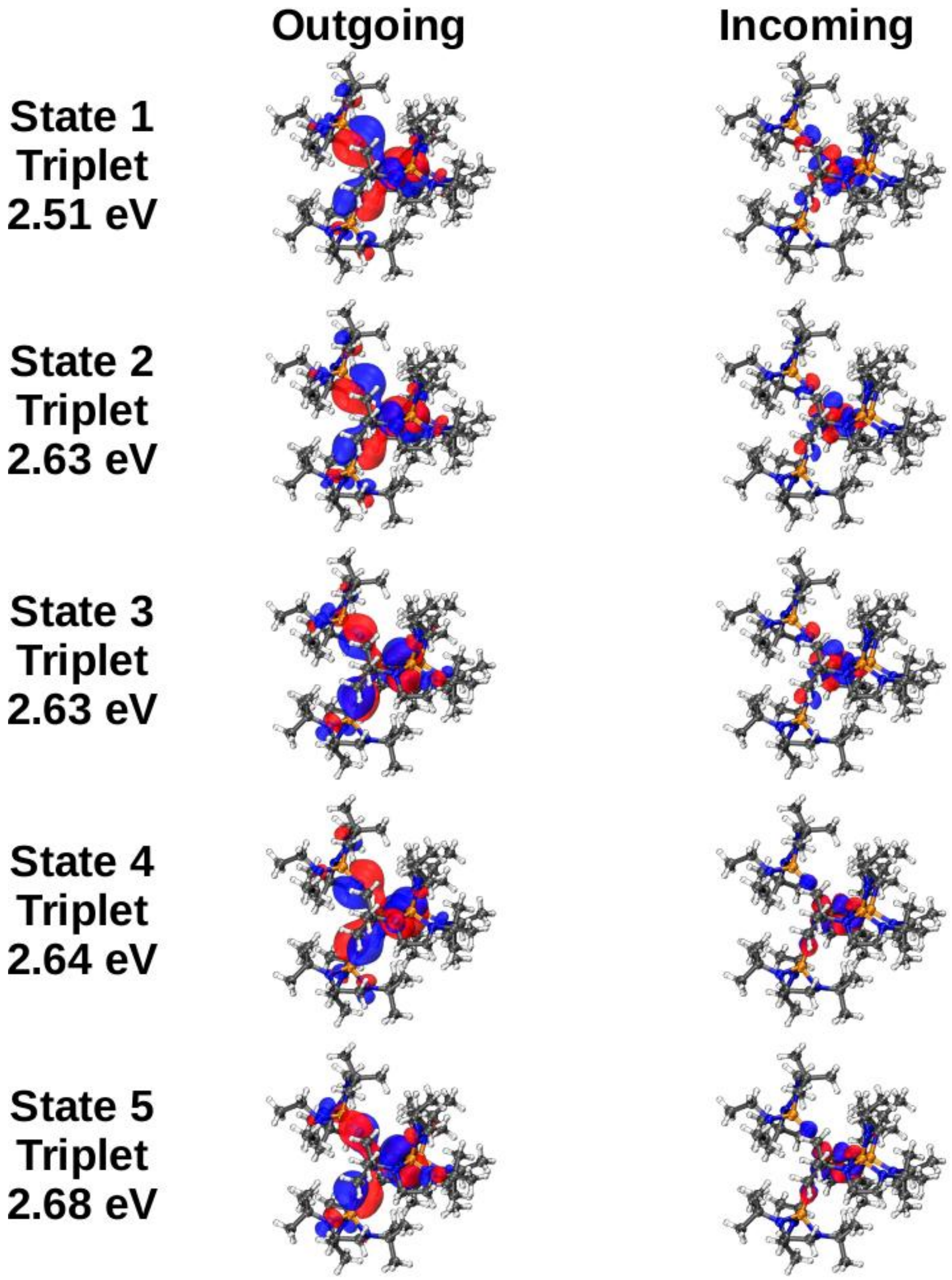

Figure S14. Natural transition orbitals of the first five excited states for **2.**

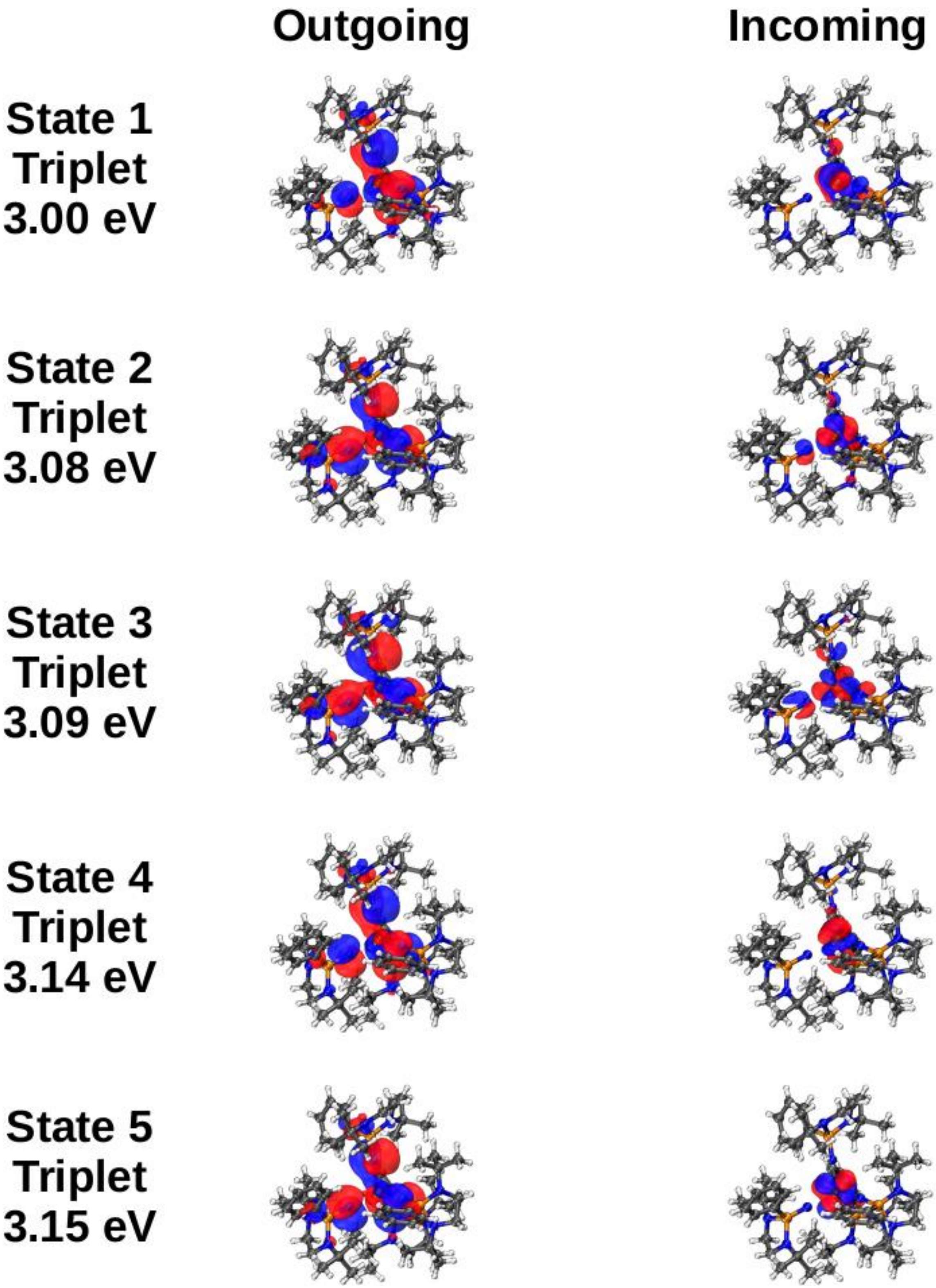

Figure S15. Natural transition orbitals of the first five excited states for **3.**

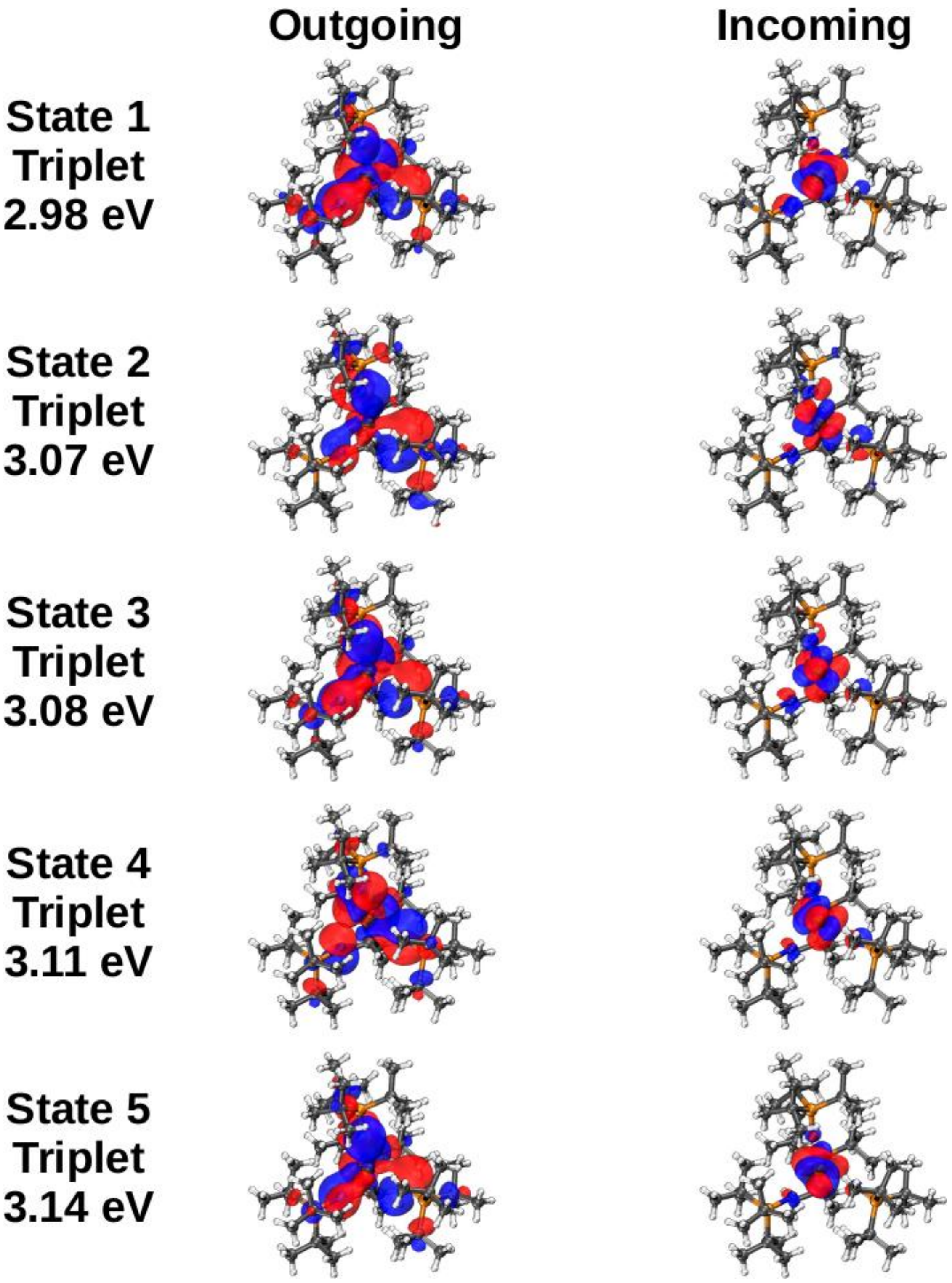

Figure S16. Natural transition orbitals of the first five excited states for **4.**

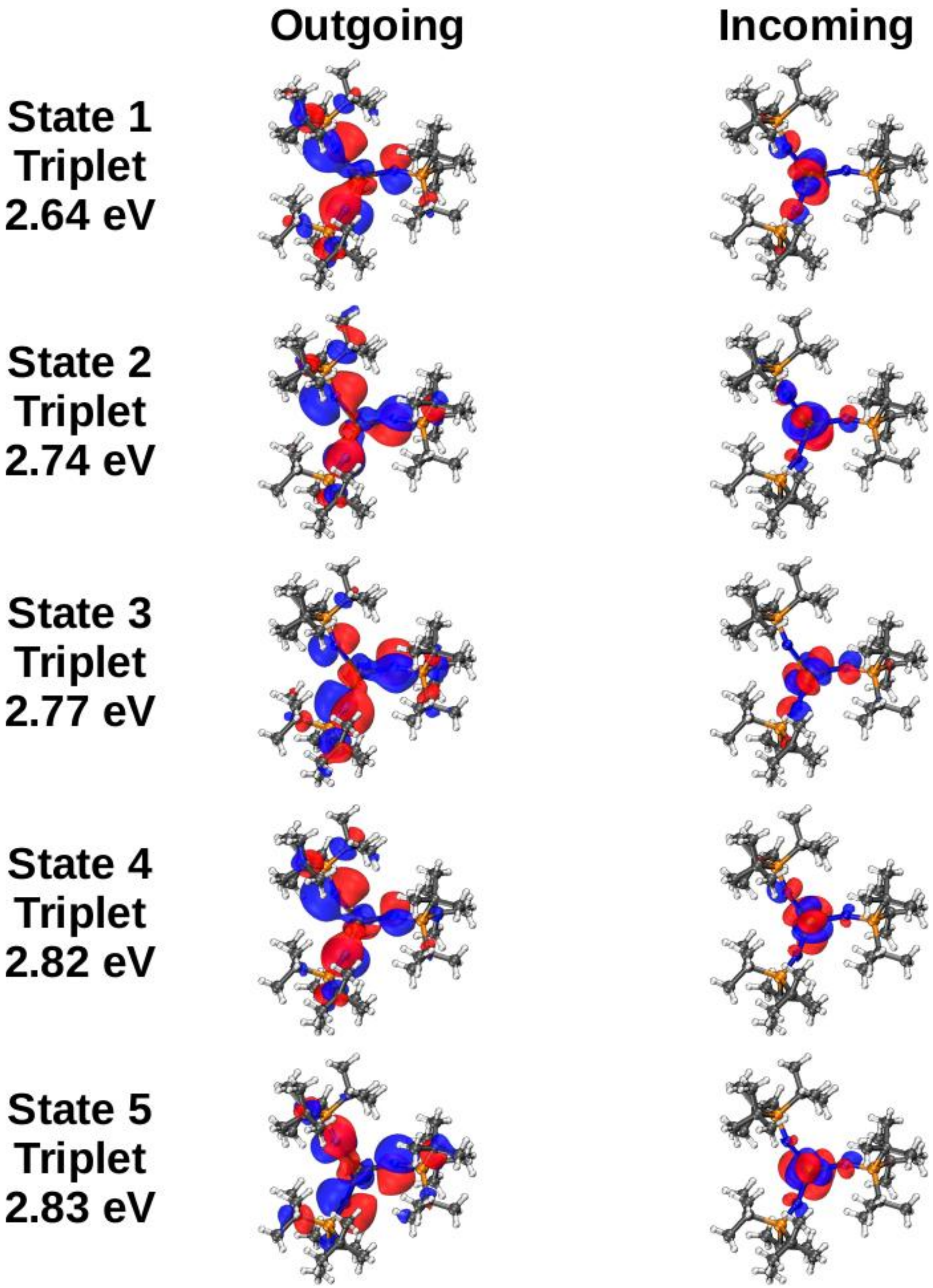

Figure S17. Natural transition orbitals of the first five excited states for **4-I.**

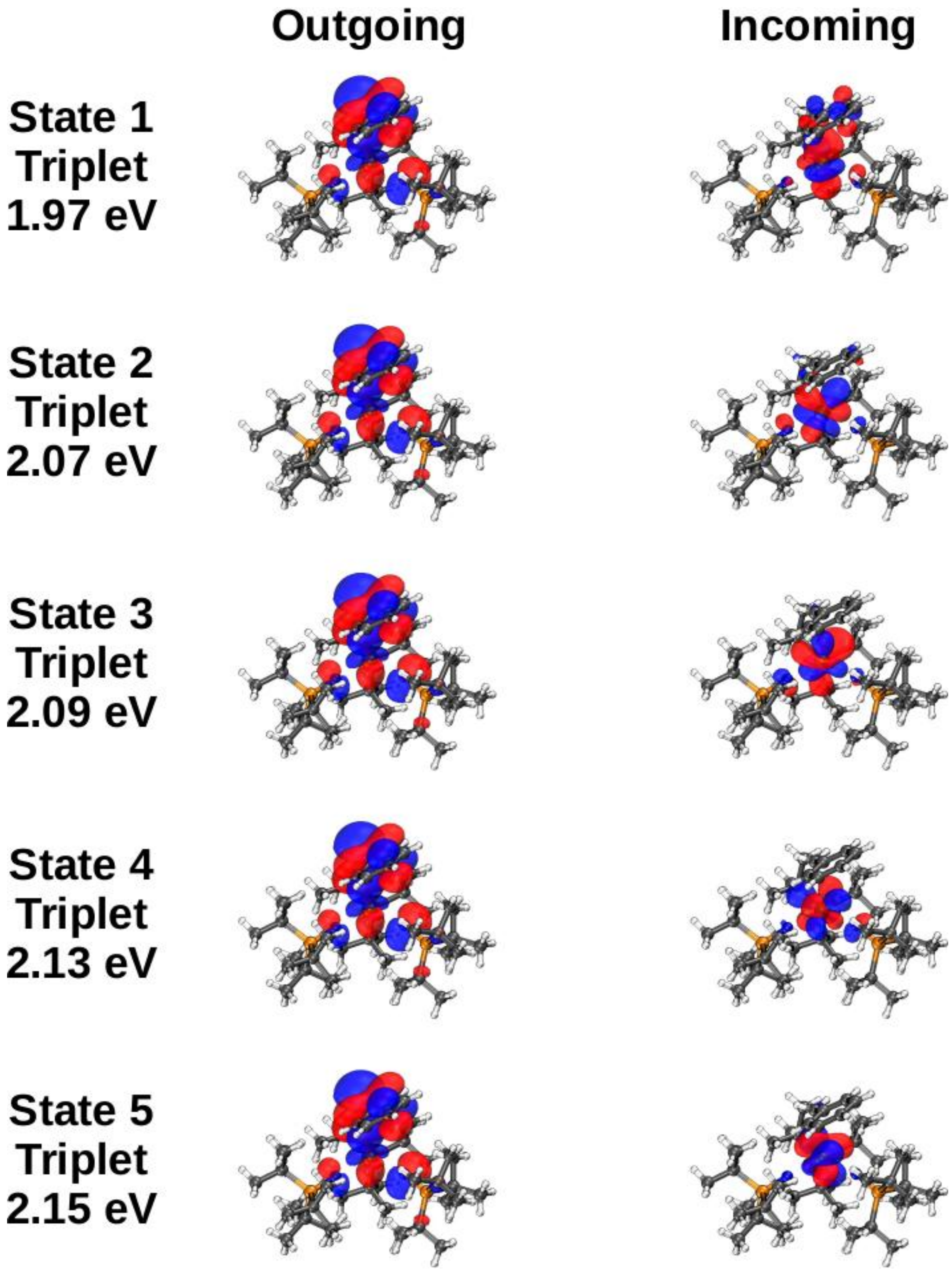

Figure S18. Natural transition orbitals of the first five excited states for **4-Bn.**

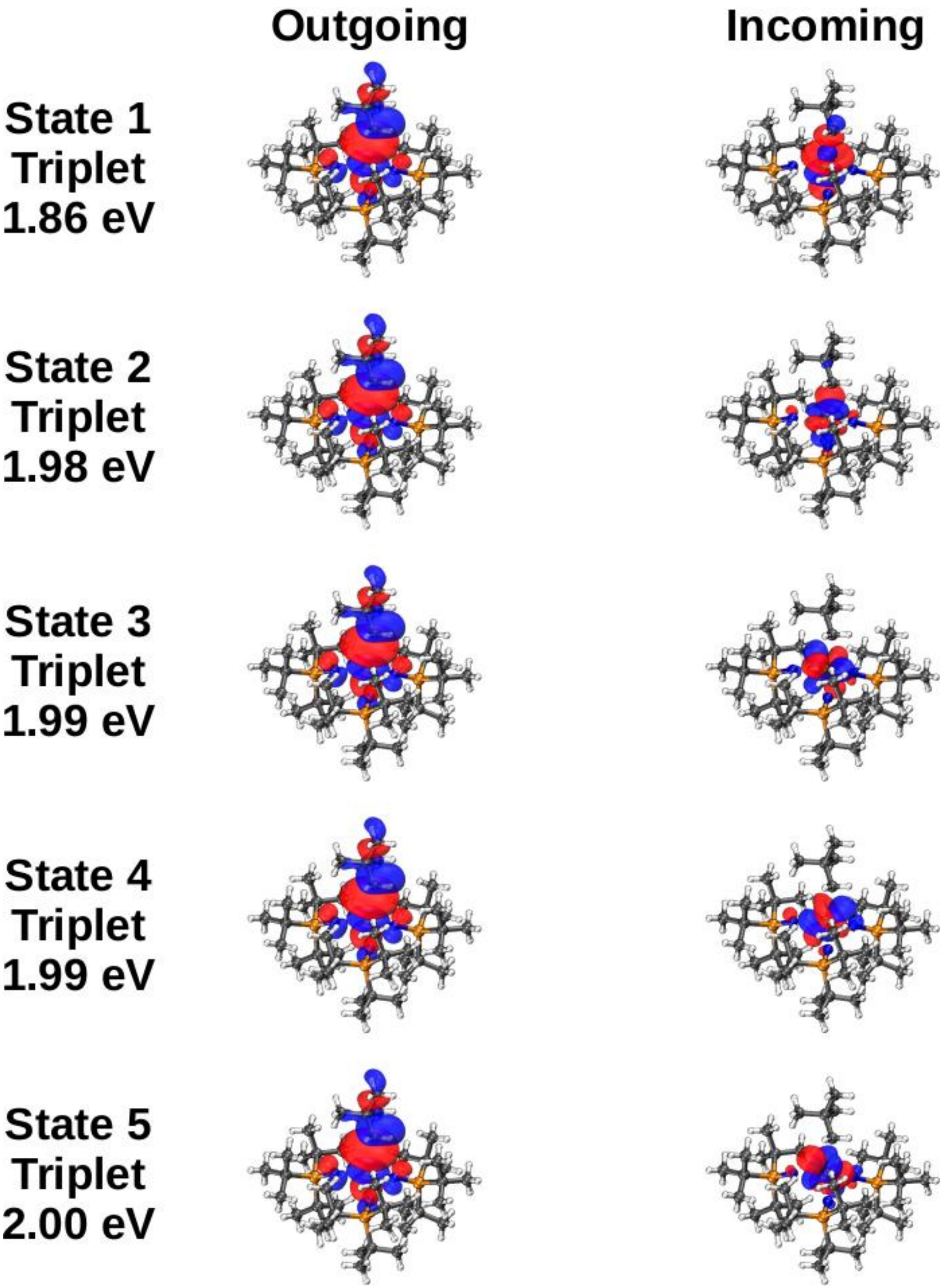

Figure S19. Natural transition orbitals of the first five excited states for **4-Npt.**

Table S7 to **Table S13** show the Mulliken populations of natural orbitals that have significant contributions from coordinating atoms and Ce f atomic orbitals (AOs). For each natural orbital, the population is resolved by atom type and by AO type. Labels with “(−Ce)” indicate that only contributions from atoms directly coordinating Ce are included for that atom type. Contributions from atom types not shown explicitly are grouped under “Others.” Occupation numbers are also reported.

Negative Mulliken populations can arise with large basis sets, particularly when diffuse functions are present. In this study, the most notable case is a 4f orbital of **3**. Its Mulliken population is 0.856 on Ce f AOs, 0.688 on Ce s AOs, and −0.502 on other ligand p AOs. We nevertheless classify this orbital as 4f because its spatial character (nodal pattern and angular shape; **Table S9**) is clearly f-like.

Table S7. Mulliken populations and occupation numbers for CASCISD natural orbitals of **1.**

Occupation Number	Mulliken Population							
	Ce				N (-Ce)		Others	
	S	P	D	F	S	P	S	P
0.999995	0	0.229	0.071	0	-0.044	0.573	0.063	0.105
0.999995	0	0.229	0.071	0	-0.044	0.573	0.063	0.105
0.999995	0	0.222	0.069	0	-0.043	0.579	0.064	0.106
0.999995	0	0.222	0.069	0	-0.043	0.579	0.064	0.106
0.999994	0.022	0.005	0.013	0	-0.001	0.623	0.127	0.208
0.999994	0.022	0.005	0.013	0	-0.001	0.623	0.127	0.208
0.999981	0	0.013	0.275	0.001	-0.056	0.634	0.025	0.107
0.999981	0	0.013	0.275	0.001	-0.056	0.634	0.025	0.107
0.999981	0	0.012	0.273	0.001	-0.056	0.636	0.026	0.108
0.999981	0	0.012	0.273	0.001	-0.056	0.636	0.026	0.108
0.999935	0.009	0.003	0.07	0.006	0.075	0.431	0.089	0.316
0.999935	0.009	0.003	0.07	0.006	0.075	0.431	0.089	0.316
0.99993	0	-0.158	0.054	0.005	0.237	0.556	0.038	0.268
0.99993	0	-0.158	0.054	0.005	0.237	0.556	0.038	0.268
0.99993	0	-0.157	0.054	0.005	0.236	0.556	0.038	0.268
0.99993	0	-0.157	0.054	0.005	0.236	0.556	0.038	0.268
0.999852	0.055	0.005	0.003	0.027	-0.069	0.562	0.05	0.365
0.999852	0.055	0.005	0.003	0.027	-0.069	0.562	0.05	0.365
0.999798	0	-0.008	-0.001	0.022	-0.001	0.616	-0.007	0.378
0.999798	0	-0.008	-0.001	0.022	-0.001	0.616	-0.007	0.378
0.999798	0	-0.008	-0.001	0.022	-0.001	0.616	-0.007	0.378
0.999798	0	-0.008	-0.001	0.022	-0.001	0.616	-0.007	0.378
0.999733	-0.028	-0.011	0.002	0.024	0.167	0.662	-0.048	0.231
0.999733	-0.028	-0.011	0.002	0.024	0.167	0.662	-0.048	0.231

0.000759	0.04	-0.002	-0.001	0.94	0.059	0	-0.043	0.008
0.000759	0.04	-0.002	-0.001	0.94	0.059	0	-0.043	0.008
0.000731	0	-0.002	0	0.966	0.002	0.004	-0.014	0.044
0.000731	0	-0.002	0	0.966	0.002	0.004	-0.014	0.044
0.000731	0	-0.002	0	0.966	0.002	0.004	-0.014	0.044
0.000731	0	-0.002	0	0.966	0.002	0.004	-0.014	0.044
0.000642	0.092	0	0.006	0.919	0.025	-0.023	-0.015	-0.004
0.000642	0.092	0	0.006	0.919	0.025	-0.023	-0.015	-0.004
0.000613	0	-0.003	0.027	0.971	0.005	-0.001	0	0.002
0.000613	0	-0.003	0.027	0.971	0.005	-0.001	0	0.002
0.000613	0	-0.003	0.027	0.971	0.005	-0.001	0	0.002
0.000613	0	-0.003	0.027	0.971	0.005	-0.001	0	0.002
0.000595	0.072	0	0.028	0.95	-0.001	-0.019	-0.02	-0.011
0.000595	0.072	0	0.028	0.95	-0.001	-0.019	-0.02	-0.011

Table S8. Mulliken populations and occupation numbers for CASCISD natural orbitals of **2.**

Occupation Number	Mulliken Population							
	Ce				N (-Ce)		Others	
	S	P	D	F	S	P	S	P
0.999995	0	0.01	0.046	0	-0.007	0.632	-0.075	0.391
0.999995	0	0.01	0.046	0	-0.007	0.632	-0.075	0.391
0.999995	0	0.01	0.046	0	-0.007	0.632	-0.075	0.391
0.999995	0	0.01	0.046	0	-0.007	0.632	-0.075	0.391
0.999995	0	0.153	0.064	0	0.133	0.538	-0.054	0.164
0.999995	0	0.153	0.064	0	0.133	0.538	-0.054	0.164
0.999984	0	0.019	0.296	0.002	-0.053	0.554	0.063	0.12
0.999984	0	0.019	0.296	0.002	-0.053	0.554	0.063	0.12
0.999982	0.009	0	0.353	0	-0.11	0.52	-0.02	0.247
0.999982	0.009	0	0.353	0	-0.11	0.52	-0.02	0.247
0.999937	0	-0.091	0.019	0.004	0.097	0.534	0.017	0.42
0.999937	0	-0.091	0.019	0.004	0.097	0.534	0.017	0.42
0.999918	0	0.013	0.042	0.008	0.106	0.533	-0.034	0.332
0.999918	0	0.013	0.042	0.008	0.106	0.533	-0.034	0.332
0.999918	0	0.013	0.042	0.008	0.106	0.533	-0.034	0.332
0.999918	0	0.013	0.042	0.008	0.106	0.533	-0.034	0.332
0.99989	-0.082	0	0.031	0.013	0.108	0.338	0.022	0.569
0.99989	-0.082	0	0.031	0.013	0.108	0.338	0.022	0.569
0.999706	-0.089	0	0.003	0.028	0.013	0.697	-0.089	0.435
0.999706	-0.089	0	0.003	0.028	0.013	0.697	-0.089	0.435
0.999696	0	-0.002	0.025	0.034	-0.012	0.682	-0.015	0.288
0.999696	0	-0.002	0.025	0.034	-0.012	0.682	-0.015	0.288

0.999696	0	-0.002	0.025	0.034	-0.012	0.682	-0.015	0.288
0.999696	0	-0.002	0.025	0.034	-0.012	0.682	-0.015	0.288
0.000899	0	0.001	0.001	0.963	-0.002	0.004	-0.015	0.047
0.000899	0	0.001	0.001	0.963	-0.002	0.004	-0.015	0.047
0.000899	0	0.001	0.001	0.963	-0.002	0.004	-0.015	0.047
0.000899	0	0.001	0.001	0.963	-0.002	0.004	-0.015	0.047
0.000887	-0.016	0	0	0.959	0.012	0.019	-0.016	0.042
0.000887	-0.016	0	0	0.959	0.012	0.019	-0.016	0.042
0.000687	0	-0.001	0.034	0.974	0	-0.001	-0.006	0.001
0.000687	0	-0.001	0.034	0.974	0	-0.001	-0.006	0.001
0.000687	0	-0.001	0.034	0.974	0	-0.001	-0.006	0.001
0.000687	0	-0.001	0.034	0.974	0	-0.001	-0.006	0.001
0.000677	0	-0.002	0.03	0.973	0.002	0.001	-0.006	0.002
0.000677	0	-0.002	0.03	0.973	0.002	0.001	-0.006	0.002
0.000663	0.216	0	-0.002	0.906	0.076	-0.052	-0.007	-0.136
0.000663	0.216	0	-0.002	0.906	0.076	-0.052	-0.007	-0.136

Table S9. Mulliken populations and occupation numbers for CASCISD natural orbitals of **3**.

Occupation Number	Mulliken Population							
	Ce				N (-Ce)		Others	
	S	P	D	F	S	P	S	P
0.999996	0.013	-0.034	0.083	0	0.127	0.689	0.028	0.091
0.999996	0.013	-0.034	0.083	0	0.127	0.689	0.028	0.091
0.999995	0.004	0.299	0.042	0	-0.133	0.501	0.059	0.225
0.999995	0.004	0.299	0.042	0	-0.133	0.501	0.059	0.225
0.999995	0	0.132	0.062	0	0.232	0.553	0.026	-0.007
0.999995	0	0.132	0.062	0	0.232	0.553	0.026	-0.007
0.999981	-0.002	0.025	0.41	0	-0.024	0.495	0.071	0.025
0.999981	-0.002	0.025	0.41	0	-0.024	0.495	0.071	0.025
0.999981	0.009	-0.001	0.486	0	-0.023	0.477	0.041	0.01
0.999981	0.009	-0.001	0.486	0	-0.023	0.477	0.041	0.01
0.999937	0.002	-0.09	-0.04	0.004	0.116	0.427	0.039	0.542
0.999937	0.002	-0.09	-0.04	0.004	0.116	0.427	0.039	0.542
0.999936	0.014	-0.082	-0.016	0.004	0.164	0.403	0.063	0.451
0.999936	0.014	-0.082	-0.016	0.004	0.164	0.403	0.063	0.451
0.999925	-0.002	-0.009	-0.023	0.005	0.239	0.412	0.02	0.358
0.999925	-0.002	-0.009	-0.023	0.005	0.239	0.412	0.02	0.358
0.999883	-0.265	0.038	0.004	0.006	0.319	0.319	0.016	0.563
0.999883	-0.265	0.038	0.004	0.006	0.319	0.319	0.016	0.563
0.999674	-0.004	-0.002	-0.001	0.033	0.011	0.64	-0.074	0.396

0.999674	-0.004	-0.002	-0.001	0.033	0.011	0.64	-0.074	0.396
0.999659	-0.003	-0.001	-0.005	0.034	0.023	0.619	-0.103	0.437
0.999659	-0.003	-0.001	-0.005	0.034	0.023	0.619	-0.103	0.437
0.999647	-0.003	0.002	0.004	0.035	0.003	0.616	-0.082	0.424
0.999647	-0.003	0.002	0.004	0.035	0.003	0.616	-0.082	0.424
0.000726	0.002	0.005	0.001	0.851	0.006	-0.006	-0.019	0.158
0.000726	0.002	0.005	0.001	0.851	0.006	-0.006	-0.019	0.158
0.000715	0	-0.006	0.001	0.865	0.008	-0.003	-0.026	0.161
0.000715	0	-0.006	0.001	0.865	0.008	-0.003	-0.026	0.161
0.000713	0	0.003	0.001	0.814	-0.002	-0.011	-0.036	0.231
0.000713	0	0.003	0.001	0.814	-0.002	-0.011	-0.036	0.231
0.000527	0.065	-0.003	0.042	0.95	0.019	-0.011	-0.014	-0.047
0.000527	0.065	-0.003	0.042	0.95	0.019	-0.011	-0.014	-0.047
0.000511	0.183	-0.002	0.036	0.923	0.028	-0.02	0.014	-0.163
0.000511	0.183	-0.002	0.036	0.923	0.028	-0.02	0.014	-0.163
0.000501	0.021	-0.003	0.042	0.95	0.007	-0.006	0.009	-0.019
0.000501	0.021	-0.003	0.042	0.95	0.007	-0.006	0.009	-0.019
0.00047	0.688	-0.005	0.017	0.856	0.101	-0.073	-0.082	-0.502
0.00047	0.688	-0.005	0.017	0.856	0.101	-0.073	-0.082	-0.502

Table S10. Mulliken populations and occupation numbers for CASCISD natural orbitals of **4.**

Occupation Number	Mulliken Population							
	Ce				N (-Ce)		Others	
	S	P	D	F	S	P	S	P
0.999993	0	0.152	0.063	0.001	0.075	0.634	0.062	0.011
0.999993	0	0.152	0.063	0.001	0.075	0.634	0.062	0.011
0.999992	0.001	0.146	0.05	0.001	0.048	0.705	0.155	-0.108
0.999992	0.001	0.146	0.05	0.001	0.048	0.705	0.155	-0.108
0.999992	0	0.102	0.047	0	0.08	0.693	0.118	-0.044
0.999992	0	0.102	0.047	0	0.08	0.693	0.118	-0.044
0.999976	0	0.025	0.24	0.001	-0.093	0.718	-0.014	0.122
0.999976	0	0.025	0.24	0.001	-0.093	0.718	-0.014	0.122
0.999974	0	-0.004	0.369	0	-0.003	0.581	0.081	-0.024
0.999974	0	-0.004	0.369	0	-0.003	0.581	0.081	-0.024
0.99991	0.001	-0.037	0.093	0.007	0.139	0.554	0.01	0.234
0.99991	0.001	-0.037	0.093	0.007	0.139	0.554	0.01	0.234
0.999908	0.001	-0.017	0.058	0.006	0.174	0.533	0.021	0.222
0.999908	0.001	-0.017	0.058	0.006	0.174	0.533	0.021	0.222

0.999901	0.001	-0.031	0.064	0.008	0.13	0.589	0.046	0.194
0.999901	0.001	-0.031	0.064	0.008	0.13	0.589	0.046	0.194
0.999793	0.341	0.003	0.001	0.034	0.058	0.399	0.01	0.154
0.999793	0.341	0.003	0.001	0.034	0.058	0.399	0.01	0.154
0.999628	-0.008	0.002	0.028	0.034	-0.016	0.728	0.028	0.205
0.999628	-0.008	0.002	0.028	0.034	-0.016	0.728	0.028	0.205
0.999624	0	-0.012	0.02	0.033	-0.015	0.733	0.035	0.205
0.999624	0	-0.012	0.02	0.033	-0.015	0.733	0.035	0.205
0.999596	-0.45	0.008	-0.003	0.029	0.186	1.034	0.059	0.137
0.999596	-0.45	0.008	-0.003	0.029	0.186	1.034	0.059	0.137
0.000989	-0.04	0.002	-0.006	0.924	0.023	0.038	-0.075	0.133
0.000989	-0.04	0.002	-0.006	0.924	0.023	0.038	-0.075	0.133
0.000985	-0.039	0.001	-0.003	0.92	0.018	0.032	-0.056	0.127
0.000985	-0.039	0.001	-0.003	0.92	0.018	0.032	-0.056	0.127
0.000983	-0.026	0.003	-0.003	0.922	0.012	0.023	-0.101	0.17
0.000983	-0.026	0.003	-0.003	0.922	0.012	0.023	-0.101	0.17
0.000795	0.198	0	-0.005	0.892	-0.018	-0.164	-0.051	0.149
0.000795	0.198	0	-0.005	0.892	-0.018	-0.164	-0.051	0.149
0.000753	0	0	0.071	0.961	-0.003	0	-0.023	-0.006
0.000753	0	0	0.071	0.961	-0.003	0	-0.023	-0.006
0.000744	0.003	0.003	0.05	0.964	0.007	-0.003	-0.008	-0.015
0.000744	0.003	0.003	0.05	0.964	0.007	-0.003	-0.008	-0.015
0.000734	0.006	-0.001	0.071	0.945	-0.001	-0.008	-0.106	0.094
0.000734	0.006	-0.001	0.071	0.945	-0.001	-0.008	-0.106	0.094

Table S11. Mulliken populations and occupation numbers for CASCISD natural orbitals of **4-I.**

Occupation Number	Mulliken Population								
	Ce				N (-Ce)		I		Others	
	S	P	D	F	S	P	S	P	S	P
0.999997	0	-0.139	0.087	0.001	0.033	0.215	0	0.742	0.062	-0.002
0.999997	0	-0.139	0.087	0.001	0.033	0.215	0	0.742	0.062	-0.002
0.999996	0	-0.149	0.099	0.001	0.031	0.204	0	0.765	0.051	-0.004
0.999996	0	-0.149	0.099	0.001	0.031	0.204	0	0.765	0.051	-0.004
0.999993	-0.206	0.219	0.025	0	0.007	0.255	0.005	0.634	0.181	-0.123
0.999993	-0.206	0.219	0.025	0	0.007	0.255	0.005	0.634	0.181	-0.123
0.999972	-0.001	0.103	0.165	0.001	-0.072	0.611	0	0.069	0.057	0.066
0.999972	-0.001	0.103	0.165	0.001	-0.072	0.611	0	0.069	0.057	0.066

0.999971	0	0.102	0.148	0.001	-0.052	0.62	0	0.069	0.053	0.057
0.999971	0	0.102	0.148	0.001	-0.052	0.62	0	0.069	0.053	0.057
0.999906	0.001	-0.069	0.091	0.008	0.162	0.53	0	0.022	-0.003	0.258
0.999906	0.001	-0.069	0.091	0.008	0.162	0.53	0	0.022	-0.003	0.258
0.999906	0.001	-0.069	0.091	0.008	0.159	0.532	0	0.026	0.008	0.243
0.999906	0.001	-0.069	0.091	0.008	0.159	0.532	0	0.026	0.008	0.243
0.999861	0.195	0.097	0.086	0.013	-0.093	0.379	0.003	0.213	0.012	0.091
0.999861	0.195	0.097	0.086	0.013	-0.093	0.379	0.003	0.213	0.012	0.091
0.999787	-0.227	-0.011	0.017	0.027	0.166	0.546	0.001	0.013	0.087	0.379
0.999787	-0.227	-0.011	0.017	0.027	0.166	0.546	0.001	0.013	0.087	0.379
0.999669	-0.001	-0.003	0.013	0.029	0.025	0.722	0	0.06	-0.022	0.175
0.999669	-0.001	-0.003	0.013	0.029	0.025	0.722	0	0.06	-0.022	0.175
0.999657	-0.006	-0.007	0.014	0.029	0.03	0.721	0	0.059	-0.012	0.17
0.999657	-0.006	-0.007	0.014	0.029	0.03	0.721	0	0.059	-0.012	0.17
0.999415	-0.12	-0.012	0.009	0.046	0.034	0.811	0	0.001	-0.01	0.24
0.999415	-0.12	-0.012	0.009	0.046	0.034	0.811	0	0.001	-0.01	0.24
0.001172	-0.073	-0.001	0.001	0.925	0.039	0.036	0	0	-0.034	0.106
0.001172	-0.073	-0.001	0.001	0.925	0.039	0.036	0	0	-0.034	0.106
0.001061	-0.002	0.032	0	0.947	0	-0.007	0	0.003	-0.009	0.033
0.001061	-0.002	0.032	0	0.947	0	-0.007	0	0.003	-0.009	0.033
0.001048	0	0.031	0	0.949	0.001	-0.007	0	0.003	-0.013	0.033
0.001048	0	0.031	0	0.949	0.001	-0.007	0	0.003	-0.013	0.033
0.000845	0.001	0.005	0.002	0.913	0.071	-0.059	0	0	-0.094	0.161
0.000845	0.001	0.005	0.002	0.913	0.071	-0.059	0	0	-0.094	0.161
0.000807	0	0	0.038	0.961	0.006	0.001	0	0	-0.004	-0.002
0.000807	0	0	0.038	0.961	0.006	0.001	0	0	-0.004	-0.002
0.000801	-0.002	0	0.036	0.961	0.006	0.002	0	0	-0.006	0.002
0.000801	-0.002	0	0.036	0.961	0.006	0.002	0	0	-0.006	0.002
0.00077	-0.004	0.002	0.019	0.957	0.022	0.024	0	0	-0.009	-0.012
0.00077	-0.004	0.002	0.019	0.957	0.022	0.024	0	0	-0.009	-0.012

Table S12. Mulliken populations and occupation numbers for CASCISD natural orbitals of **4-Bn.**

Occupation Number	Mulliken Population									
	Ce				N (-Ce)		C (-Ce)		Others	
	S	P	D	F	S	P	S	P	S	P
0.99999	-0.043	0.102	0.01	0	0.04	0.436	0.02	0.072	0.049	0.312
0.99999	-0.043	0.102	0.01	0	0.04	0.436	0.02	0.072	0.049	0.312

0.999988	-0.005	-0.087	0.055	0	0.044	0.316	0.001	-0.001	0.031	0.645
0.999988	-0.005	-0.087	0.055	0	0.044	0.316	0.001	-0.001	0.031	0.645
0.999976	0.002	0.048	0.22	0	0.027	0.602	-0.001	0.001	0.028	0.072
0.999976	0.002	0.048	0.22	0	0.027	0.602	-0.001	0.001	0.028	0.072
0.999974	-0.006	0.084	0.193	0.001	-0.026	0.566	0.001	0.006	0.046	0.134
0.999974	-0.006	0.084	0.193	0.001	-0.026	0.566	0.001	0.006	0.046	0.134
0.999934	-0.001	-0.05	0.086	0.007	0.144	0.465	0.006	0.013	0.057	0.273
0.999934	-0.001	-0.05	0.086	0.007	0.144	0.465	0.006	0.013	0.057	0.273
0.999919	-0.01	-0.035	0.098	0.008	0.168	0.493	0.002	0.007	0.018	0.25
0.999919	-0.01	-0.035	0.098	0.008	0.168	0.493	0.002	0.007	0.018	0.25
0.999853	0.015	0.085	0.06	0.013	0.03	0.473	0.004	0.085	0.029	0.205
0.999853	0.015	0.085	0.06	0.013	0.03	0.473	0.004	0.085	0.029	0.205
0.99975	-0.001	0.104	0.014	0.022	-0.052	0.662	-0.001	0	0.059	0.193
0.99975	-0.001	0.104	0.014	0.022	-0.052	0.662	-0.001	0	0.059	0.193
0.999736	-0.276	0.018	0.024	0.021	0.238	0.632	0.019	0.04	0.019	0.265
0.999736	-0.276	0.018	0.024	0.021	0.238	0.632	0.019	0.04	0.019	0.265
0.999491	0.036	0.002	0.003	0.042	-0.018	0.619	0.007	0.066	0.041	0.202
0.999491	0.036	0.002	0.003	0.042	-0.018	0.619	0.007	0.066	0.041	0.202
0.999444	0.078	0.002	0.024	0.043	-0.03	0.357	0.034	0.259	0.034	0.199
0.999444	0.078	0.002	0.024	0.043	-0.03	0.357	0.034	0.259	0.034	0.199
0.001088	0.043	0	0.008	0.906	-0.002	-0.016	0.002	0.004	-0.032	0.088
0.001088	0.043	0	0.008	0.906	-0.002	-0.016	0.002	0.004	-0.032	0.088
0.001	-0.001	0.002	0.003	0.928	0.001	0.003	0	0	-0.008	0.074
0.001	-0.001	0.002	0.003	0.928	0.001	0.003	0	0	-0.008	0.074
0.000804	0.031	0.024	0.016	0.942	-0.005	-0.026	-0.001	-0.006	-0.008	0.033
0.000804	0.031	0.024	0.016	0.942	-0.005	-0.026	-0.001	-0.006	-0.008	0.033
0.000774	0.105	0.022	0.009	0.909	0.024	-0.038	0.026	0.003	0	-0.061
0.000774	0.105	0.022	0.009	0.909	0.024	-0.038	0.026	0.003	0	-0.061
0.000676	-0.02	0.002	0.041	0.917	0.027	0.001	-0.001	0.008	-0.005	0.03
0.000676	-0.02	0.002	0.041	0.917	0.027	0.001	-0.001	0.008	-0.005	0.03
0.000641	0.005	0.006	0.048	0.963	0.004	-0.005	0	0.001	-0.023	0.002
0.000641	0.005	0.006	0.048	0.963	0.004	-0.005	0	0.001	-0.023	0.002
0.000604	0.09	-0.009	0.071	0.941	-0.013	-0.013	0.023	0.001	-0.126	0.035
0.000604	0.09	-0.009	0.071	0.941	-0.013	-0.013	0.023	0.001	-0.126	0.035

Table S13. Mulliken populations and occupation numbers for CASCISD natural orbitals of **4-Npt.**

Occupation Number	Mulliken Population								
	Ce				N (-Ce)		C (-Ce)		Others	
	S	P	D	F	S	P	S	P	S	P
0.99999	0.004	0.025	-0.006	0	0	0.018	0.001	0.15	0.249	0.559
0.99999	0.004	0.025	-0.006	0	0	0.018	0.001	0.15	0.249	0.559
0.999979	-0.007	0.02	0.018	0	0.001	0.023	-0.019	0.247	0.221	0.495
0.999979	-0.007	0.02	0.018	0	0.001	0.023	-0.019	0.247	0.221	0.495
0.999927	0	0.196	0.015	0	-0.01	0.571	0.014	0.169	0.043	0.001
0.999927	0	0.196	0.015	0	-0.01	0.571	0.014	0.169	0.043	0.001
0.999921	0	0.195	0.226	0.001	-0.083	0.571	-0.005	0.012	0.047	0.034
0.999921	0	0.195	0.226	0.001	-0.083	0.571	-0.005	0.012	0.047	0.034
0.99987	0.009	0.065	0.246	0.001	0.003	0.626	-0.001	-0.001	0.113	-0.062
0.99987	0.009	0.065	0.246	0.001	0.003	0.626	-0.001	-0.001	0.113	-0.062
0.999784	0.002	-0.083	0.101	0.007	0.195	0.532	0.002	0.007	0.022	0.215
0.999784	0.002	-0.083	0.101	0.007	0.195	0.532	0.002	0.007	0.022	0.215
0.999777	0	-0.124	0.103	0.006	0.205	0.596	0.001	-0.002	0.033	0.182
0.999777	0	-0.124	0.103	0.006	0.205	0.596	0.001	-0.002	0.033	0.182
0.999703	-0.017	0.011	0.1	0.015	0.089	0.439	0.013	0.074	0.021	0.254
0.999703	-0.017	0.011	0.1	0.015	0.089	0.439	0.013	0.074	0.021	0.254
0.999562	0.076	0.127	0.001	0.02	-0.081	0.56	-0.004	0.04	0.017	0.242
0.999562	0.076	0.127	0.001	0.02	-0.081	0.56	-0.004	0.04	0.017	0.242
0.998713	0.017	-0.015	0.076	0.021	0.005	0.642	0.011	0.013	0.011	0.219
0.998713	0.017	-0.015	0.076	0.021	0.005	0.642	0.011	0.013	0.011	0.219
0.998683	-0.031	0.002	0.037	0.03	-0.018	0.264	0.079	0.395	0.042	0.199
0.998683	-0.031	0.002	0.037	0.03	-0.018	0.264	0.079	0.395	0.042	0.199
0.998475	-0.291	0.009	0.039	0.036	0.11	0.839	0.021	0.067	0	0.17
0.998475	-0.291	0.009	0.039	0.036	0.11	0.839	0.021	0.067	0	0.17
0.000981	-0.063	0.003	0.005	0.919	0.049	0.024	0.026	0.024	-0.042	0.054
0.000981	-0.063	0.003	0.005	0.919	0.049	0.024	0.026	0.024	-0.042	0.054
0.000894	0.047	0.007	0.001	0.94	-0.022	-0.048	-0.013	0.022	-0.028	0.095
0.000894	0.047	0.007	0.001	0.94	-0.022	-0.048	-0.013	0.022	-0.028	0.095
0.000836	-0.005	0.011	0.017	0.953	0	0	0	0	-0.026	0.049
0.000836	-0.005	0.011	0.017	0.953	0	0	0	0	-0.026	0.049
0.000831	0.008	0.026	0.002	0.957	-0.003	-0.008	0.001	-0.001	-0.043	0.061
0.000831	0.008	0.026	0.002	0.957	-0.003	-0.008	0.001	-0.001	-0.043	0.061
0.000723	0.002	-0.002	0.069	0.963	0.004	-0.001	0	-0.005	-0.042	0.012
0.000723	0.002	-0.002	0.069	0.963	0.004	-0.001	0	-0.005	-0.042	0.012

0.000711	-0.001	0.008	0.055	0.964	0	-0.003	0	0.002	-0.023	-0.001
0.000711	-0.001	0.008	0.055	0.964	0	-0.003	0	0.002	-0.023	-0.001
0.000659	0.275	-0.008	0.055	0.909	-0.019	-0.061	0.005	-0.003	-0.176	0.023
0.000659	0.275	-0.008	0.055	0.909	-0.019	-0.061	0.005	-0.003	-0.176	0.023

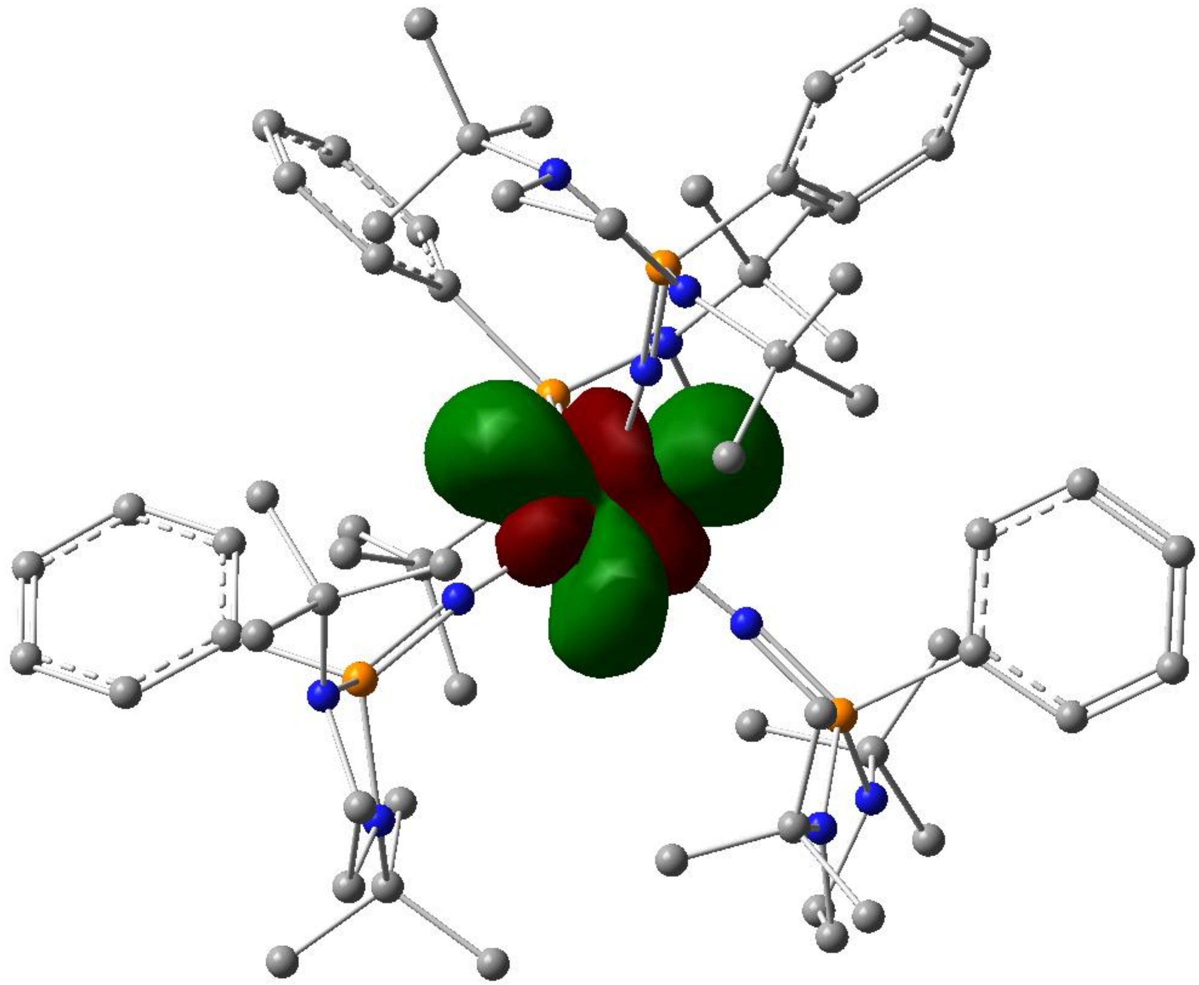

Figure S 20. Natural orbital of 3 with Mulliken populations of 0.856 on Ce f AOs, 0.688 on Ce s AOs, and −0.502 on other ligand p AOs. The isosurface was generated with an isovalue of 0.02.

Electrochemistry

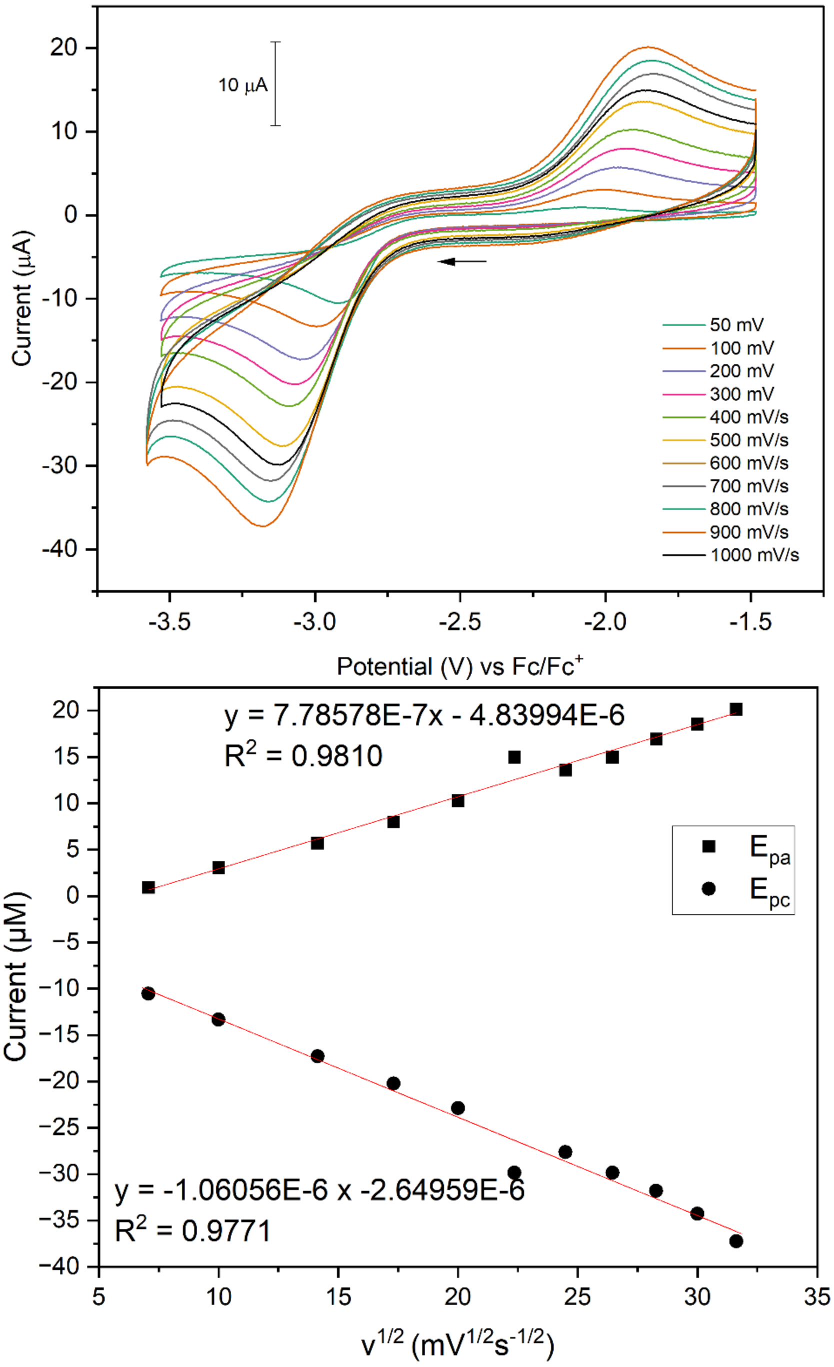

Figure S21. Scan-rate dependence of cyclic voltammogram of 3 mM **3** in 0.1M [nBu_4N][PF_6] in THF (top) and Randles-Sevcik plot (bottom).

UV-vis NIR Electronic Absorption Spectra

UV-Vis-NIR spectrum of **4-I** in toluene has been reported previously[12], but in order to allow for a direct comparison, a new UV-vis spectra was collected as shown in Figure S23. The newly acquired data in THF for **4-I** was used for UV-Vis-NIR comparison in this work. Complexes **4-Bn** and **4-Npt**.

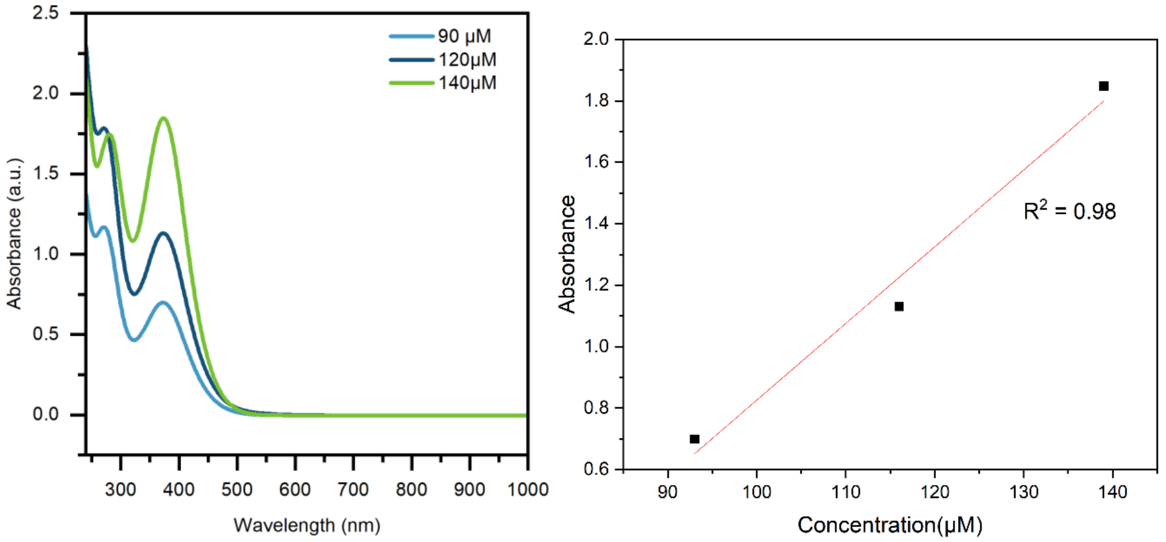

Figure S22. (Left) UV-vis-NIR spectra of 3 in THF. (Right) Linear regression on absorbance at λ_{max}= 373 nm.

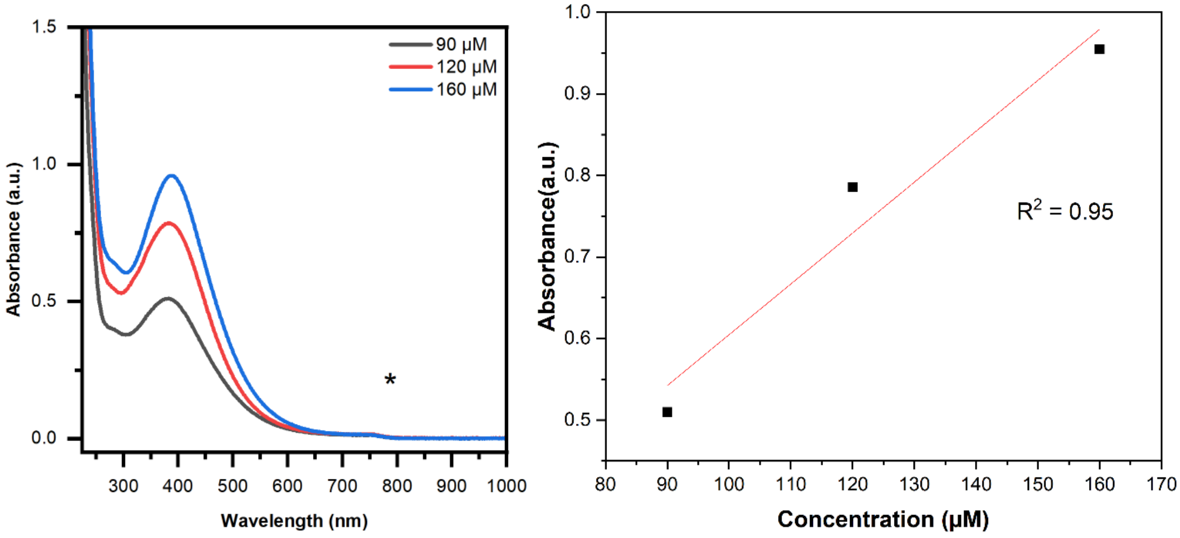

Figure S23. (Left) UV-vis-NIR spectra of 4-I in THF. (Right) Linear regression on absorbance at λ_{max}= 382 nm.

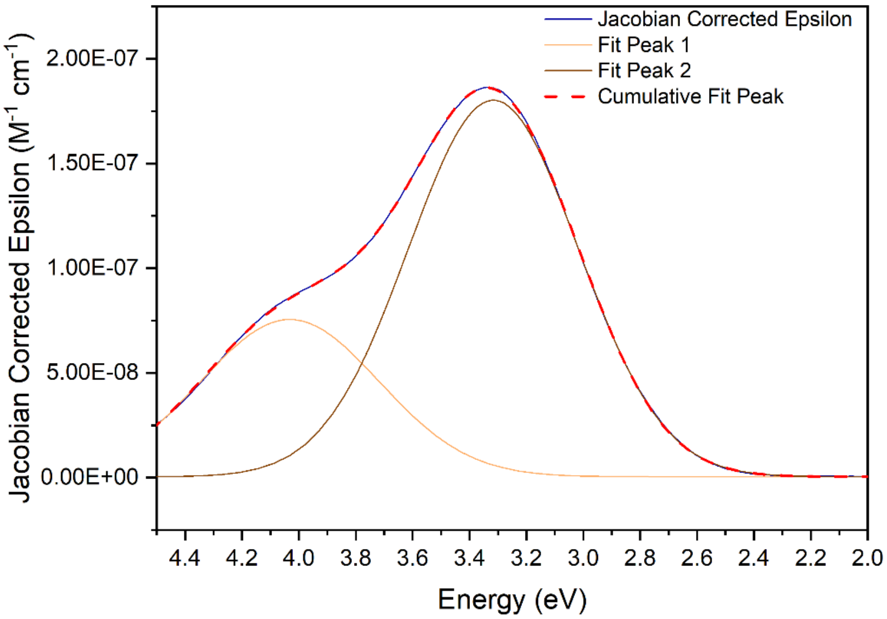

Figure S24. Fitting of previously reported[19] experimental UV-vis spectrum of **4** using two Gaussian functions. As the x-axis was converted from wavelength to energy, Jacobian transformation [20] was applied to ensure that the integration of the area under the curve remains consistent. Two Gaussian functions were fitted in Origin Lab using Peak Analyzer function.

Table S14. Fit results of previously reported[19] experimental UV-vis spectrum of **4** with two Gaussian functions, as shown in **Figure S24**. The centroid of LMCT peak, λ_{cent} was derived as a weighted (by area) average of the two peaks.

	Energy (eV)	Area	FWHM (eV))
Fit Peak 1	3.316(4)	1.3458E-7	0.70334
Fit Peak 2	4.031(1)	5.90073E-8	0.7384
	E_{cent} (eV)		
**4**	3.53(33)		

Ce L_3-edge X-ray Absorption Transmission Near Edge Spectroscopy (XANES)

Ce L_3-edge transmission XANES data for **1**, **2**, **4** has been previously reported, and reported normalized data was used for this analysis.

L_3-edge transmission XANES spectra were collected at Stanford Synchrotron radiation Lightsource(SSRL) in Menlo Park, CA at BL 7-3(**3**, **4-I**) and BL4-3(**4-Bn**, **4-Npt**).

Crystalline samples were shipped to the beamline in flame-sealed glass ampoules. Boron nitride(BN) was dried under vacuum at >250°C for 24 hours prior to use, and shipped in glass ampoules. Inside of an Ar glovebox, a ~1:1 mixture (by mass) of an analyte and BN were ground to a fine powder using a mortar and pestle. Solid state samples were loaded into an aluminum plate with 1/32" thickness equipped with a 3 x 15 mm^2 oval window and screw holes. One side of the plate was covered with 0.5 mil Kapton tape. The sample was loaded onto the plate with uniform thickness, and sealed with another piece of 0.5 mil Kapton tape. Then the aluminum plate was fastened into the sample holder, secured with screws on the corner. The sample holder was then taken outside of the glovebox, immediately submerged in LN_2 and transported to the beamline. At the beamline, the sample holder was attached to the sample rod while submerged in LN_2, and immediately inserted into the cryostat. The cryostat was cycled.

Data were calibrated to the energy of the first inflection point of the K-edge of chromium foil (5989 eV). Data was collected in triplicate, then aligned, calibrated and normalized and averaged using Athena. In order to maintain consistency in the fits and the derived n_f values, all spectra were refitted using lmfit[21], using a Pseudo-Voigt function:

$$f(x; A, \mu, \sigma, \alpha) = \frac{(1-\alpha)A}{\sigma_g\sqrt{2\pi}} e^{[-(x-\mu)^2/2\sigma_g{}^2]} + \frac{\alpha A}{\pi}\left[\frac{\sigma}{(x-\mu)^2+\sigma^2}\right] \quad (1)$$

Full-width-at-half-maximum(FWHM) can be described as 2σ, and considering that this is a symmetric function, σ is equal to half-width-at-half-maximum(HWHM). This value σ was constrained to obtain satisfactory fits. For the fitting, a three-peak model was employed. While from the L_3-edge transmission data alone it is hard to determine whether a 3 peak model or a 2 peak model is more appropriate, the further resolved HERFD-XANES data shows that a 3 peak model would be more reasonable, which is in agreement with our previous report on 1 and 2.[10] n_f values were calculated as:

$$n_f = \frac{A(p2+p3)}{A(p2+p3+p4)} \quad (2)$$

Summary of n_f values are shown in **Table S15**.

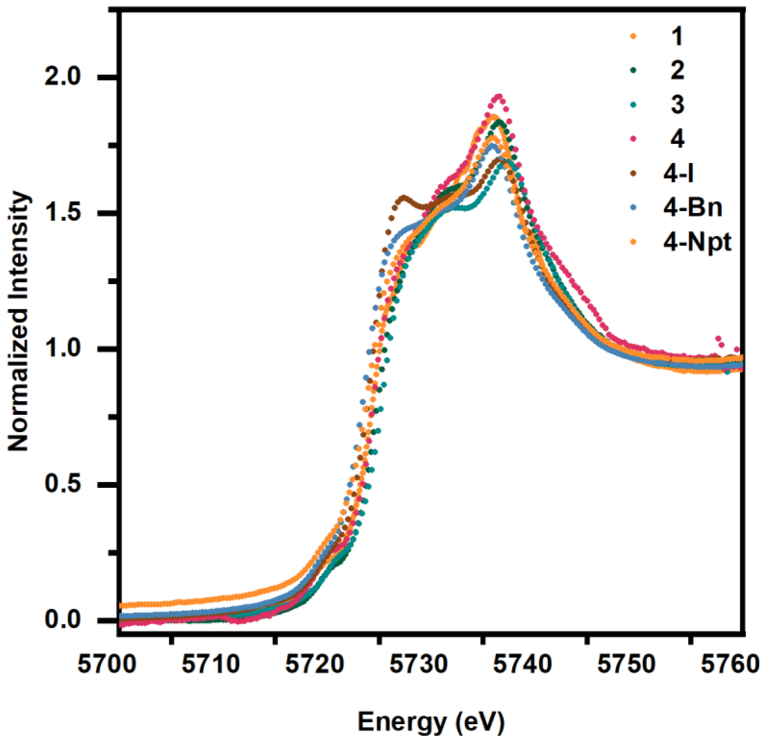

Figure S25. Overlayed L3-edge transmission XANES spectra of Ce^{4+} imidophosphorane complexes.

Table S15. Tabulated n_f values derived from Ce L_3-edge transmission XANES.

Complex	n_f
**1**	0.37(3)
**2**	0.40(1)
**3**	0.43(1)
**4**	0.36(1)
**4-I**	0.49(1)
**4-Bn**	0.37(1)
**4-Npt**	0.36(1)

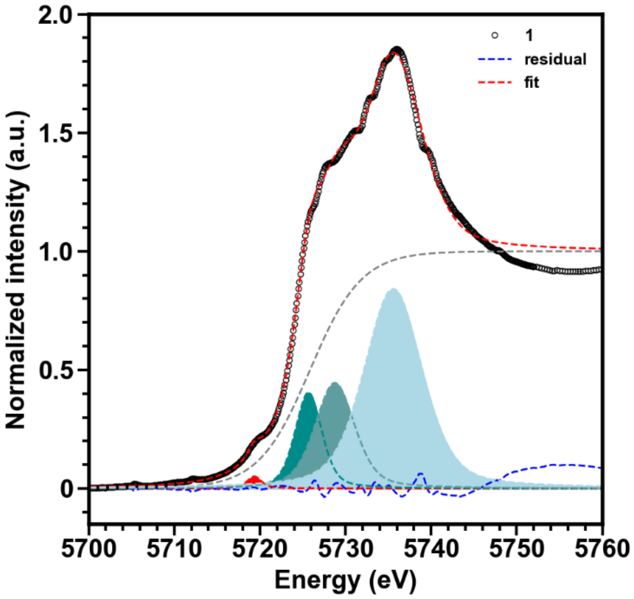

Figure S26. Ce L_3-edge transmission XANES fit for **1**. The features are shown in the following colors: pre-edge = red, p2 = dark cyan, p3 = turquoise, p4 =light blue. Steps are shown in grey, overall sum of fits are shown in red, and residuals are shown in blue.

Table S16. Fit parameters for L_3-edge transmission XANES of **1**.

	Energy (eV)	σ	Area
Step	5725.8(2)	3(fixed)	1(fixed)
pre-edge	5719.3(2)	1(fixed)	0.14 (fixed)
p2	5725.7(1)	1.8(1)	1.9(5)
p3	5728.7(3)	2.7(2)	3.1(6)
p4	5735.57(4)	4(fixed)	8.54(9)

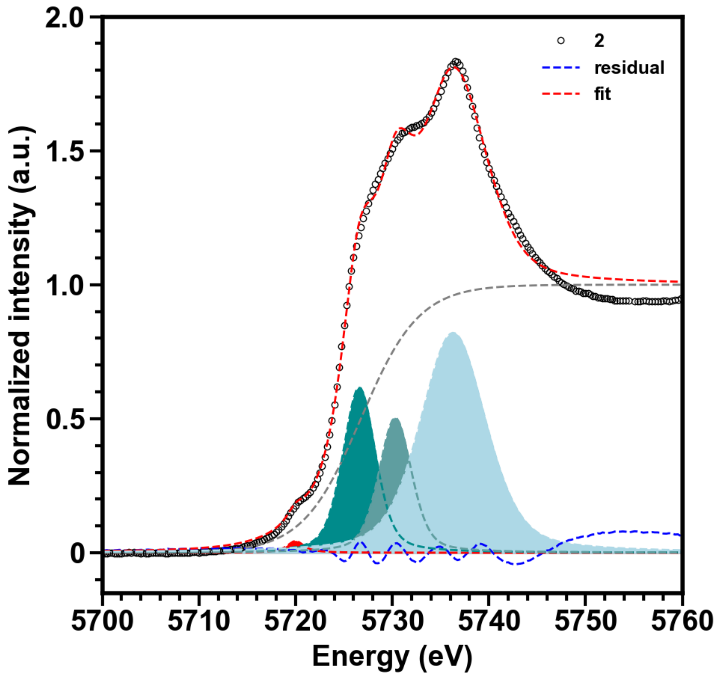

Figure S27. Ce L_3-edge transmission XANES fit for **2**. The features are shown in the following colors: pre-edge = red, p2 = dark cyan, p3 = turquoise, p4 =lightblue. Steps are shown in grey, overall sum of fits are shown in red, and residuals are shown in blue.

Table S17. Fit parameters for L_3-edge transmission XANES of **2**.

	Energy (eV)	σ	Area
Step	5726.7(2)	3(fixed)	1(fixed)
pre-edge	5720.0(4)	1.0(9)	0.14(fixed)
p2	5726.58(7)	2(fixed)	3.1(1)
p3	5730.31(8)	2(fixed)	2.5(1)
p4	5736.29(6)	4(fixed)	8.33(9)

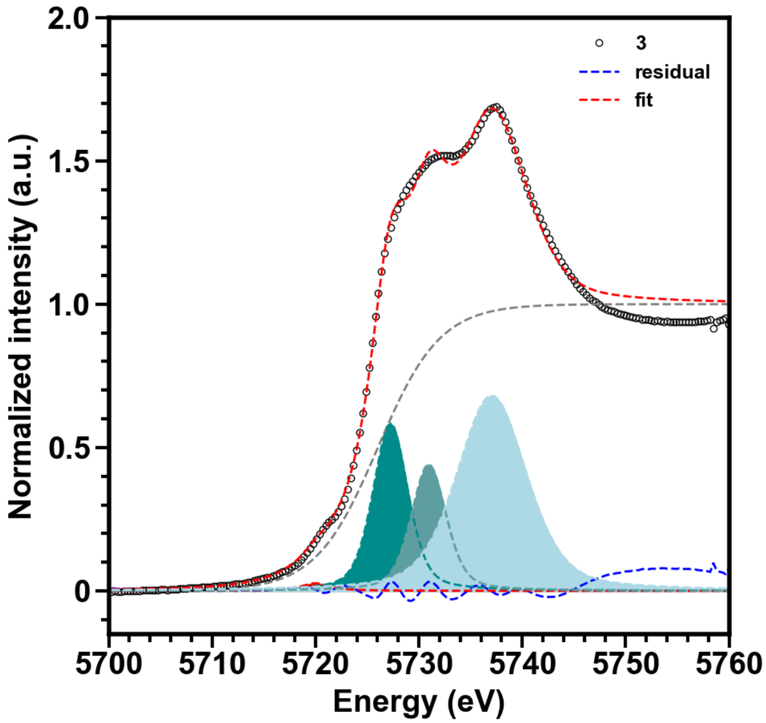

Figure S28. Ce L_3-edge transmission XANES fit for **3**. The features are shown in the following colors: pre-edge = red, p2 = dark cyan, p3 = turquoise, p4 =lightblue. Steps are shown in grey, overall sum of fits are shown in red, and residuals are shown in blue.

Table S18. Fit parameters for L_3-edge transmission XANES of **3**.

	Energy (eV)	σ	Area
Step	5726.0(2)	3(fixed)	1(fixed)
pre-edge	5720.0(9)	1(2)	0.14(fixed)
p2	5727.25(8)	2(fixed)	3.0(1)
p3	5730.9(1)	2(fixed)	2.2(1)
p4	5737.04(7)	4(fixed)	6.91(9)

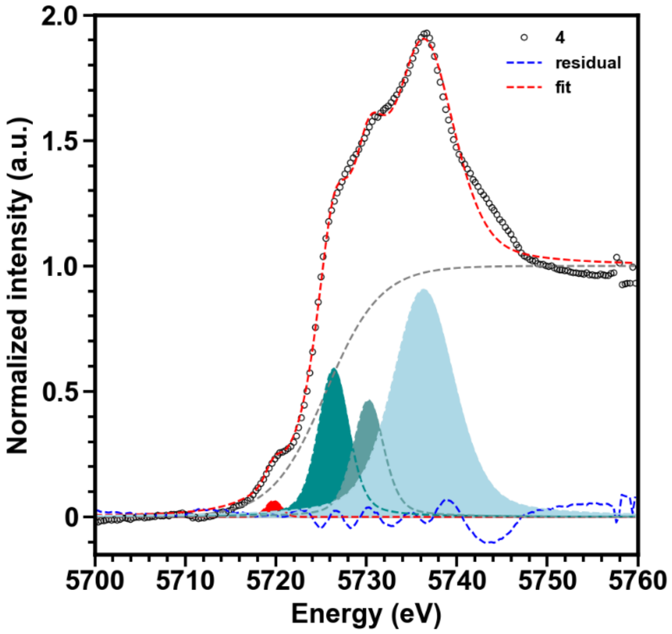

Figure S29. Ce L_3-edge transmission XANES fit for **4**. The features are shown in the following colors: pre-edge = red, p2 = dark cyan, p3 = turquoise, p4 =lightblue. Steps are shown in grey, overall sum of fits are shown in red, and residuals are shown in blue.

Table S19. Fit parameters for L_3-edge transmission XANES of **4**.

	Energy (eV)	σ	Area
Step	5725.5(2)	3(fixed)	1(fixed)
pre-edge	5719.7(4)	1.0(5)	0.14(fixed)
p2	5726.40(8)	2(fixed)	2.9(1)
p3	5730.2(1)	2(fixed)	2.3(1)
p4	5736.32(6)	4(fixed)	9.2(1)

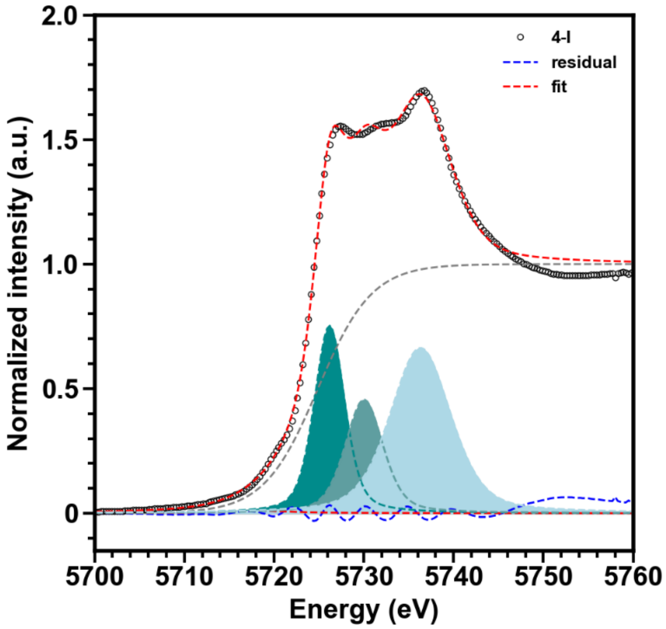

Figure S30. Ce L_3-edge transmission XANES fit for **4-I**. The features are shown in the following colors: pre-edge = red, p2 = dark cyan, p3 = turquoise, p4 =lightblue. Steps are shown in grey, overall sum of fits are shown in red, and residuals are shown in blue.

Table S20. Fit parameters for L_3-edge transmission XANES of **4-I**.

	Energy (eV)	σ	Area
Step	5724.8(3)	3(fixed)	1(fixed)
pre-edge	5718(6)	4.58	0.14
p2	5726.18(5)	2(fixed)	3.8(1)
p3	5730.0(1)	2.5(fixed)	2.9(1)
p4	5736.30(7)	4(fixed)	6.73(9)

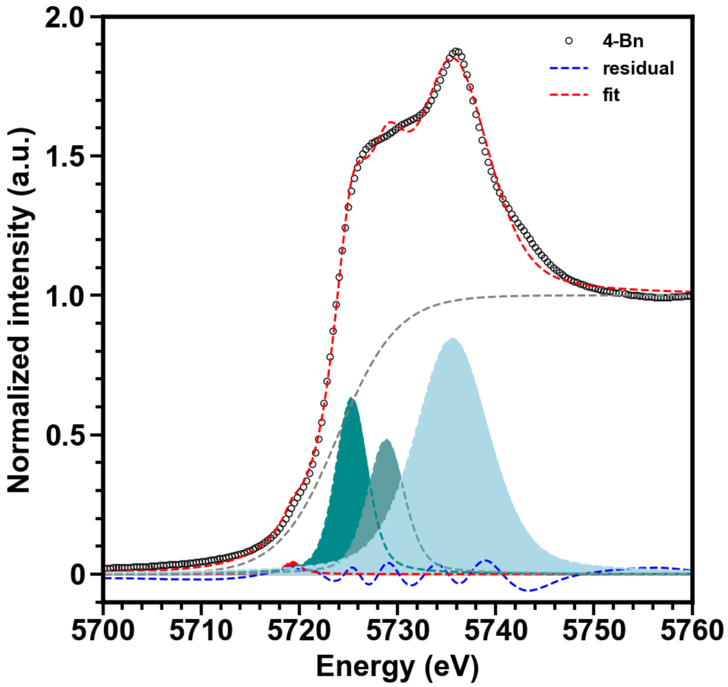

Figure S31. Ce L_3-edge transmission XANES fit for **4-Bn**. The features are shown in the following colors: pre-edge = red, p2 = dark cyan, p3 = turquoise, p4 =lightblue. Steps are shown in grey, overall sum of fits are shown in red, and residuals are shown in blue.

Table S21. Fit parameters for L_3-edge transmission XANES of **4-Bn.**

	Energy (eV)	σ	Area
Step	5724.0(1)	3(fixed)	1(fixed)
pre-edge	5719.2(3)	1(fixed)	0.14(fixed)
p2	5725.32(5)	1.9(fixed)	3.05(8)
p3	5728.83(8)	2.2(fixed)	2.69(8)
p4	5735.57(4)	4.4(fixed)	9.42(7)

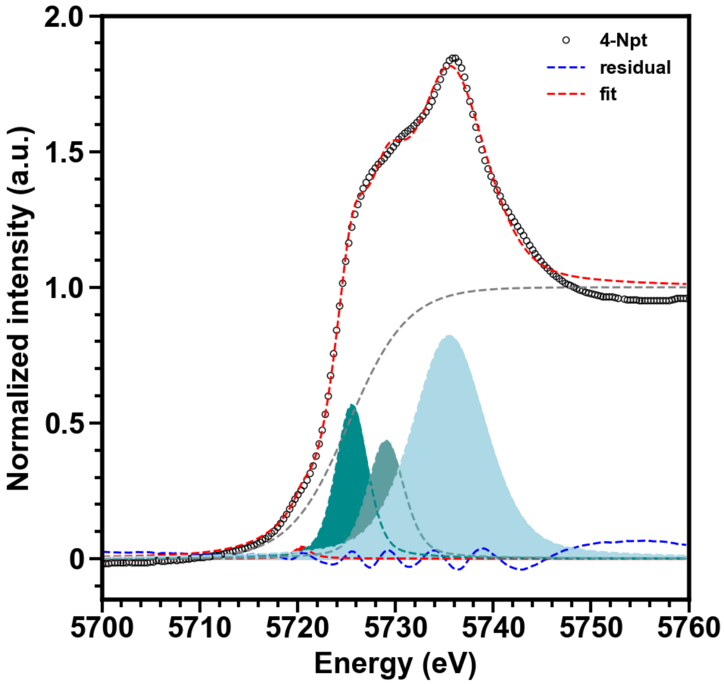

Figure S32. Ce L_3-edge transmission XANES fit for **4-Npt**. The features are shown in the following colors: pre-edge = red, p2 = dark cyan, p3 = turquoise, p4 =lightblue. Steps are shown in grey, overall sum of fits are shown in red, and residuals are shown in blue.

Table S 22. Fit parameters for L_3-edge transmission XANES of **4-Npt.**

	Energy (eV)	σ	Area
Step	5725.2(2)	3(fixed)	1(fixed)
pre-edge	5720.4(4)	1(fixed)	0.14(fixed)
p2	5725.55(8)	1.9(fixed)	2.7(1)
p3	5729.0(1)	2.2(fixed)	2.4(1)
p4	5735.50(6)	4.4(fixed)	9.1(1)

Ce L_3-edge High Energy Resolution Fluorescence Detection (HERFD)-XANES and Resonant Inelastic X-ray Scattering(RIXS)

HERFD-XANES spectra of **1**, **2** and **3** were collected at SSRL at BL 15-2. HERFD-XANES spectra of **4**, **4-I**, **4-Bn**, **4-Npt and 4^{Cs}** were collected at European Synchrotron Radiation Facility (ESRF) in Grenoble, France at BM 20. CeO_2 (≥ 99.9%, Alfa Aesar) was dried in the box furnace using alumina crucibles (MTI) at 500° C for 12 h in ambient atmosphere prior to use. For both measurements, CeO_2 was used as a reference compound to validate the experimental setup and acquired data.

Sample preparation methods for all measurements: Crystalline samples were shipped to the beamline in flame-sealed glass ampoules. Boron nitride (BN, Sigma) was dried under vacuum at >250 ^{o}C for 24 hr prior to use, and sealed and shipped in glass ampoules. Inside of Ar glovebox at SSRL/ESRF, a mixture of analyte and BN were ground to a fine powder using a mortar and pestle. Analyte was diluted with BN so that the edge jump of Ce was calculated to be approx. one absorption length. Solid state samples were loaded into an aluminum plate with 1/32" thickness equipped with a 3 x 15 mm^2 oval window and screw holes. One side of the plate was covered with 0.5 mil Kapton tape. The sample was loaded onto the plate with uniform thickness, and sealed with another piece of 0.5 mil Kapton tape.

1, 2 and **3**: (continued from **Sample preparation methods for all measurements**) The aluminum plate was fastened into the sample holder, secured with screws on the corner. The sample holder was then taken outside of the glovebox, immediately submerged in LN_2 and transported to the beamline. At the beamline, the sample holder was attached to the sample rod while submerged in LN_2, and immediately inserted into the cryostat.

HERFD-XANES spectra were collected at SSRL BL 15-2 under dedicated operating conditions (3 GeV, 5%, total of 500 mA using top-off injections). Measurement was conducted at 10K in a LHe cryostat (Oxford). Upon insertion of the sample rod at the beamline, the cryostat was cycled five times between inert He and vacuum. Incident energy was selected using LN_2-cooled Si (311) double-crystal monochrometer, oriented at $\phi = 0^{o}$. A 7-crystal Johann-type hard x-ray spectrometer[22] was used to select the emission energy at $L\alpha_1$ = 4839 eV with crystal analyzer Ge(331) aligned at Bragg angle of 80.7 degrees. The beam size under this setup was 90 (vertical) x 590 μm^2 (horizontal). For every scan, the beam was directed at different pixels of the sample in order to minimize beam damage. Data was calibrated to a known glitch on the crystal (5733.3 eV) , iterated through all scans and then the scans were averaged. Averaged scans were then normalized in PyMca v5.6.7[23].

4, 4-I, 4-Bn, 4-Npt and 4^{Cs}:

(continued from **Sample preparation methods for all measurements**) The aluminum plate was then taken out of the glovebox, immediately submerged in LN_2 and transported to the beamline. At the beamline, the sample holder was removed from the LN_2 bath and quickly clamped into the instrument cryostream.

HERFD-XANES spectra were collected at ESRF BM20[24] under dedicated operating conditions (6 GeV, 200 mA in 7/8+1 mode (192+8 mA) with 1 h refill frequency). Measurements were conducted at 100K in an LN_2 cryostream. Incident energy was selected using an LN_2-cooled

Si(111) double crystal monochromator, oriented at ϕ = 0º. A 5-crystal Johann-type spectrometer[25] with Ge (331) crystal analyzer aligned at Bragg angle of 80.7 degrees was used to select the maximum of the emission energy at 4840.3 eV. The beam size under this setup was 2000 x 75 μm^2. For every scan, the beam was directed at different pixels of the sample to minimize beam damage. The incident energy was calibrated by setting the maximum of the derivative of the HERFD-XANES of CeO_2 to 5723.2 eV. Data were normalized to the incoming flux and averaged across triplicate scans using PyMca v5.9.2[23].

RIXS: RIXS spectra of **4** and **4^{Cs}** were collected at ESRF BM20 with the same experimental setup as HERFD-XANES spectra, and individual slices were scaled to the relative sample concentration of the pixel as determined by a whole sample scan performed prior to RIXS collection.

Fitting Details: Calibrated, averaged and normalized data was fitted with a Psuedo-Voigt function using lmfit in python. Fitting details described in the section before, Ce L_3-edge Transmission XANES was also employed for HERFD-XANES fitting.

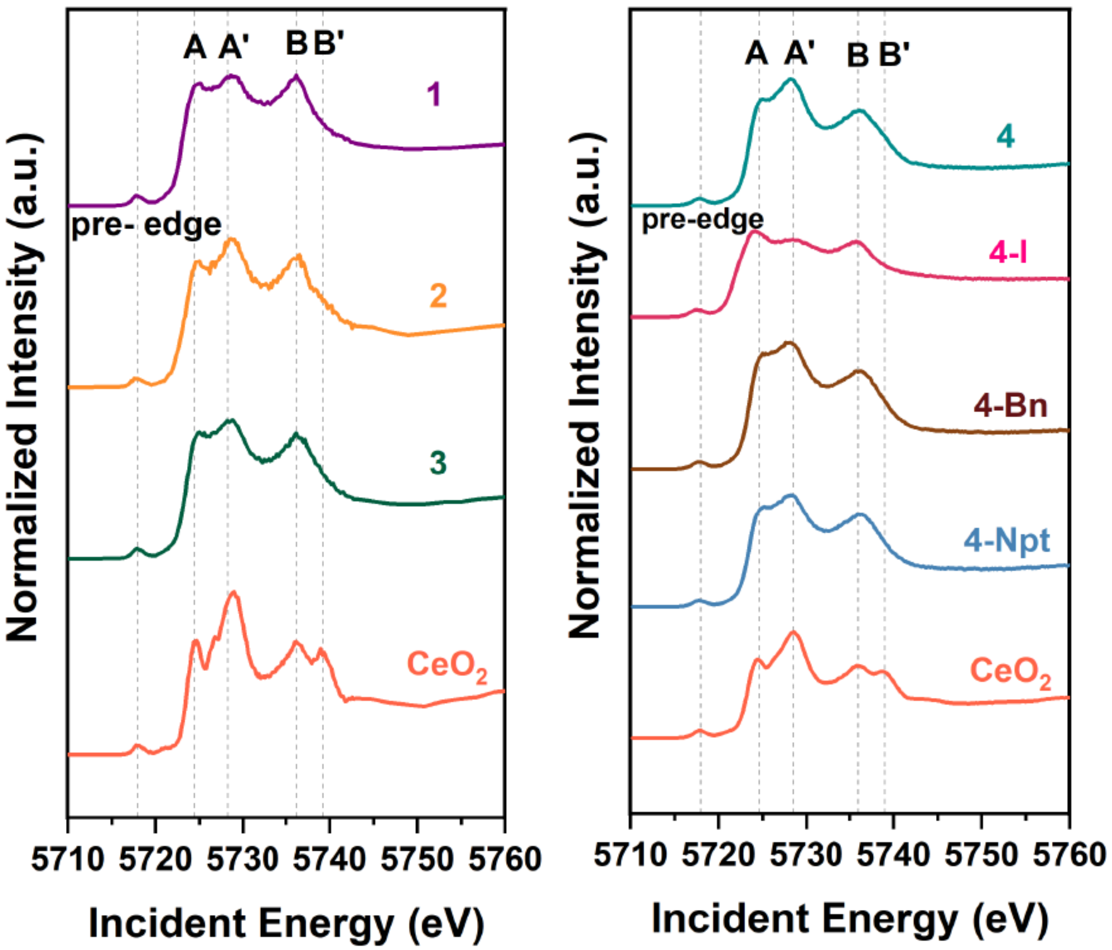

Figure S33. Overlayed HERFD-XANES spectra of (left) **1**,**2**,**3**,and CeO_2 collected at SSRL BL15-2 and (right) **4**, **4-I**, **4-Bn** and **4-Npt** with CeO_2 collected at ESRF BM20, under experimental conditions described.

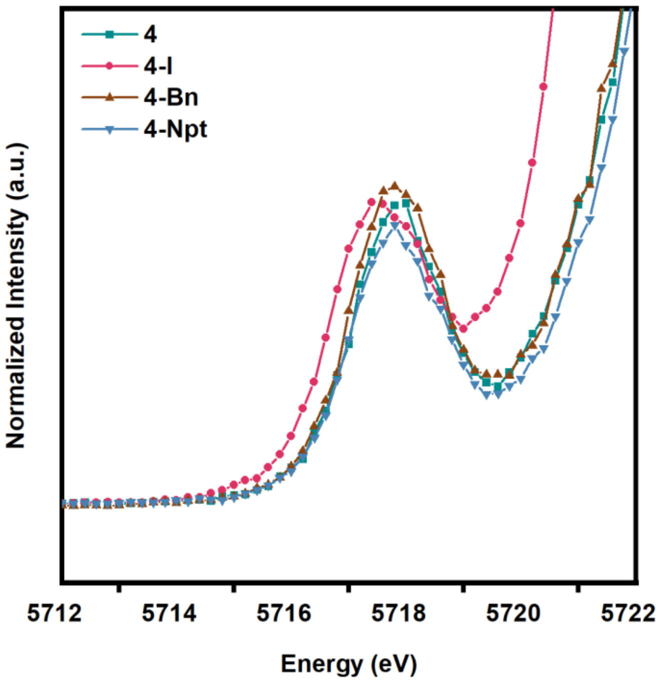

Figure S34. Overlayed pre-edge region of Ce L_3-edge HERFD-XANES of complexes **4**, **4-I**, **4-Bn** and **4-Npt**. Shift of the pre-edge peak of **4-I** shifted to lower energy, along with the rising edge which also shifted to lower energy, indicating reduction of **4-I** in the beam.

Discussion on the partial reduction observed in the HERFD-XANES spectrum of 4-I:

In **Figure S34**, the pre-edge peak of **4-I** shifted to lower energy, compared to other complexes (5717.50(6) eV). This on its own does not suggest a reduction, however, the emergence of a lower energy peak at 5715 eV as well as the lowering of the rising edge energy which is distinct from complexes **4-I**, **4-Bn** and **4-Npt** are suggestive of a partial reduction of the sample. Since the reduction is partial, there are still some Ce^{4+} samples left which is why the pre-edge has not completely shifted 2 eV lower. This observation is consistent with previously reported partial reduction of Ce^{4+} samples. [26]“

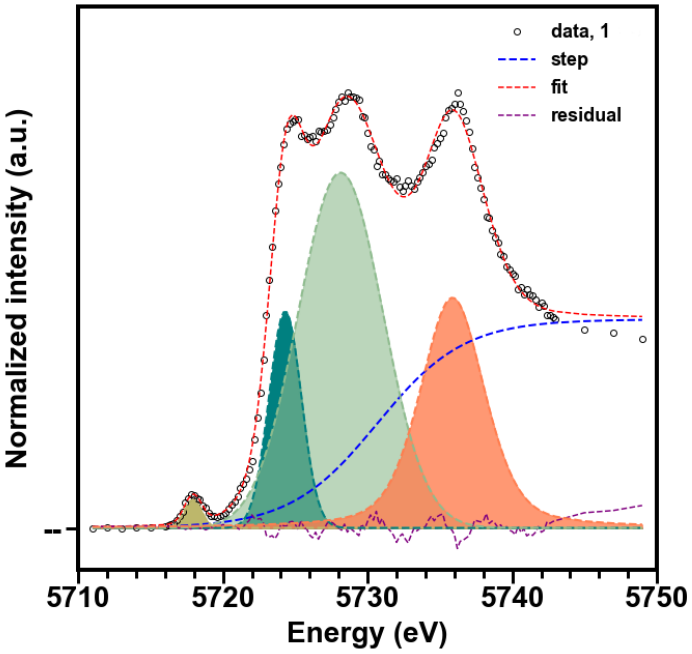

Figure S35. Ce L_3-edge HERFD-XANES fit for **1**. The features are showing in the following colors: pre-edge feature = tan green, A = teal, A' = pastel green, B = coral. Steps are shown in blue, overall sum of fits are shown in red, and residuals are shown in purple.

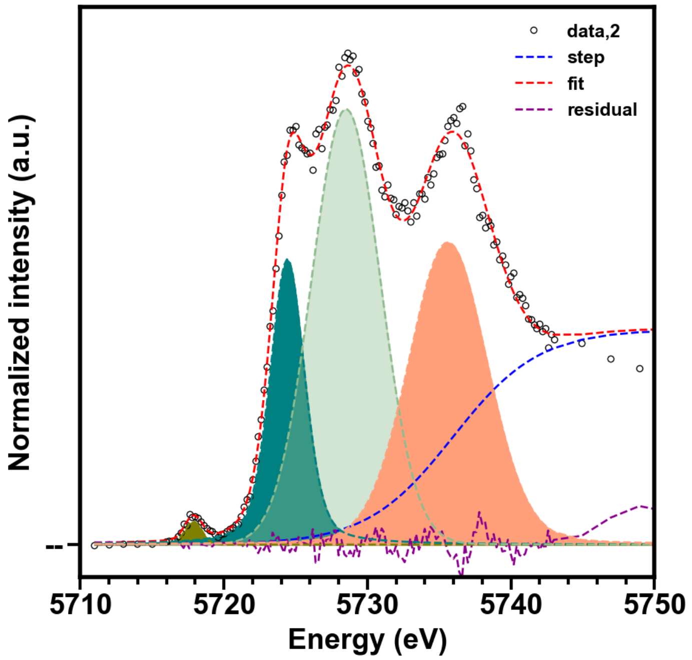

Figure S36. Ce L_3-edge HERFD-XANES fit for **2**. The features are showing in the following colors: pre-edge feature)= tan green, A = teal, A' = pastel green, B = coral. Steps are shown in blue, overall sum of fits are shown in red, and residuals are shown in purple.

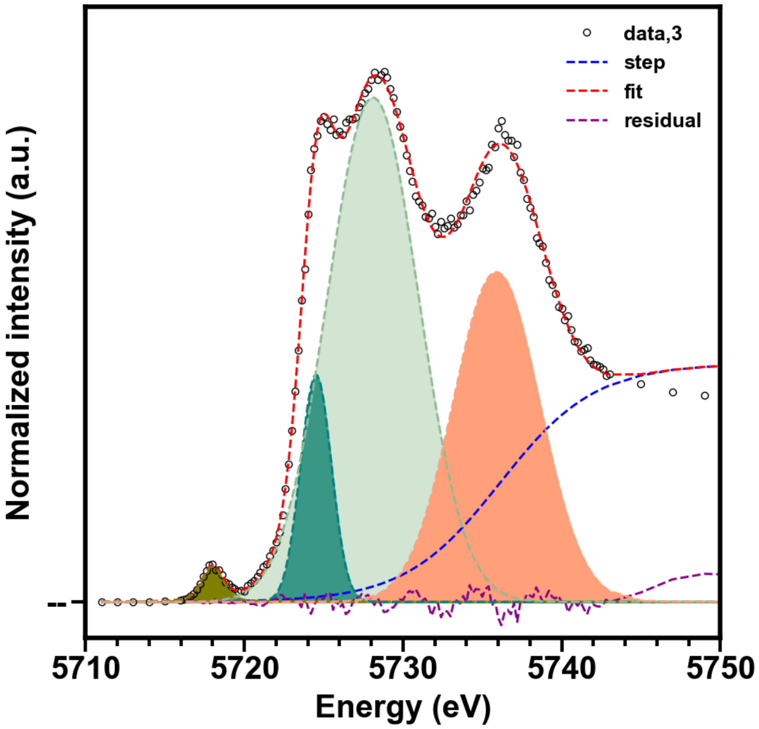

Figure S37. Ce L_3-edge HERFD-XANES fit for **3**. The features are showing in the following colors: pre-edge feature = tan green, A = teal, A' = pastel green, B = coral. Steps are shown in blue, overall sum of fits are shown in red, and residuals are shown in purple.

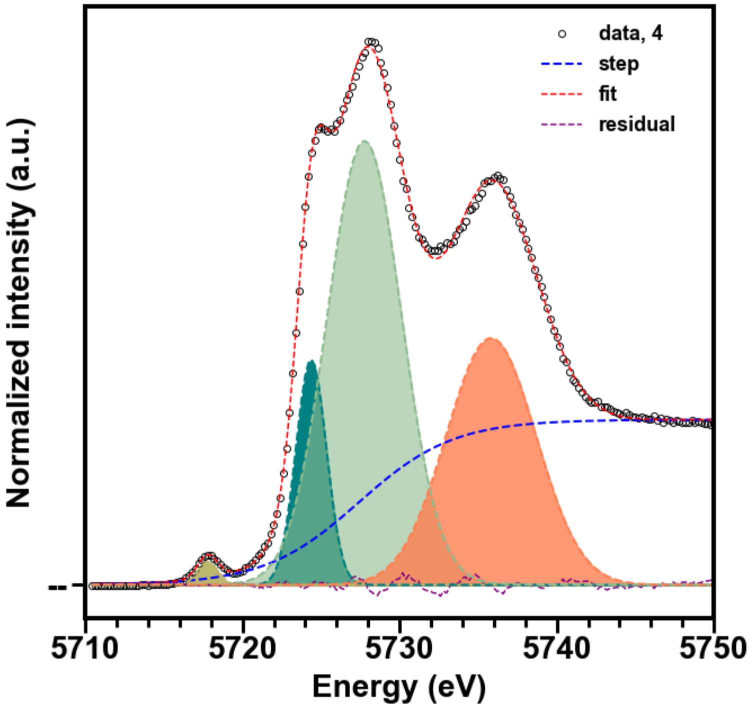

Figure S38. Ce L_3-edge HERFD-XANES fit for **4.** The features are showing in the following colors: pre-edge feature = tan green, A = teal, A' = pastel green, B = coral. Steps are shown in blue, overall sum of fits are shown in red, and residuals are shown in purple.

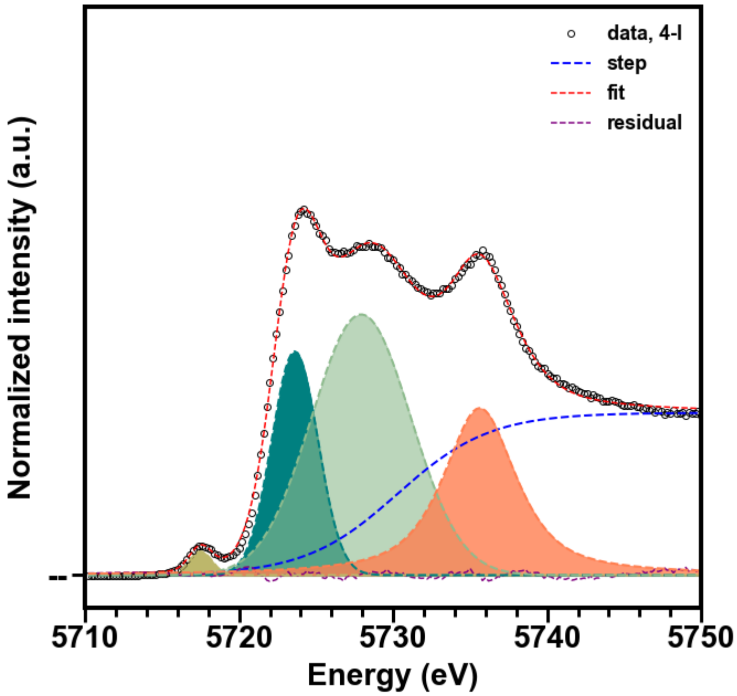

Figure S39. Ce L_3-edge HERFD-XANES fit for **4-I**. The features are showing in the following colors: pre-edge feature = tan green, A = teal, A' = pastel green, B = coral. Steps are shown in blue, overall sum of fits are shown in red, and residuals are shown in purple.

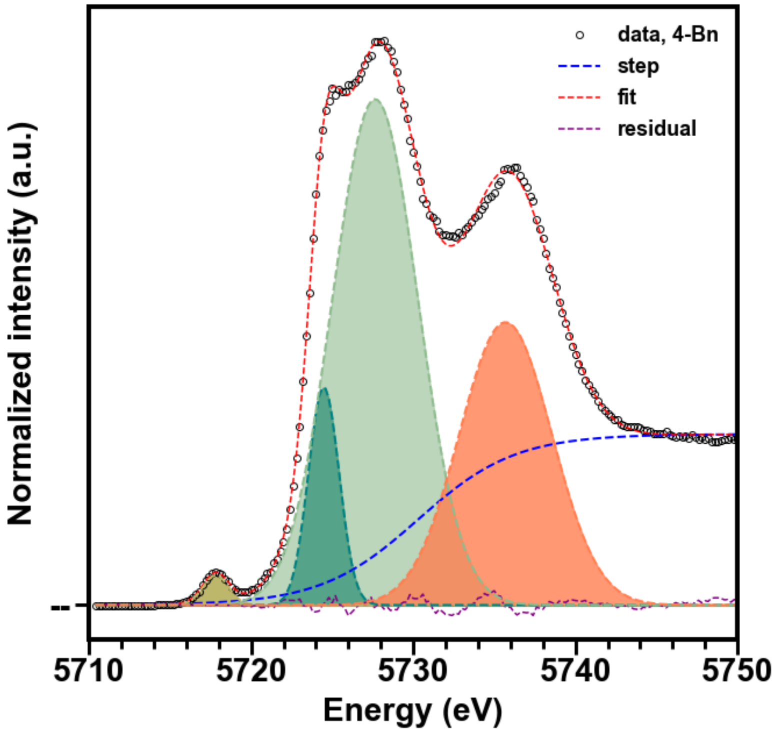

Figure S40. Ce L_3-edge HERFD-XANES fit for **4-Bn**. The features are showing in the following colors: pre-edge feature = tan green, A = teal, A' = pastel green, B = coral. Steps are shown in blue, overall sum of fits are shown in red, and residuals are shown in purple.

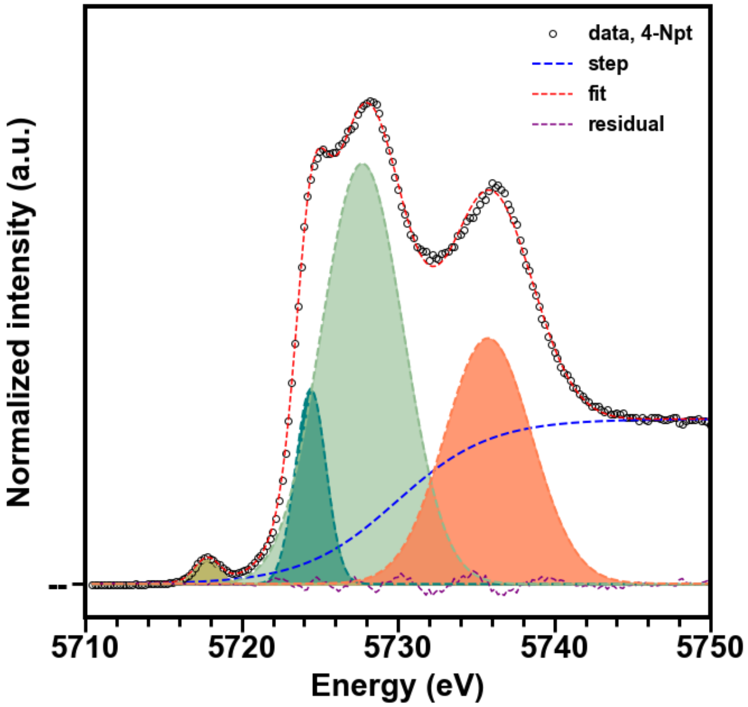

Figure S41. Ce L_3-edge HERFD-XANES fit for **4-Npt**. The features are showing in the following colors: pre-edge feature = tan green, A = teal, A' = pastel green, B = coral. Steps are shown in blue, overall sum of fits are shown in red, and residuals are shown in purple.

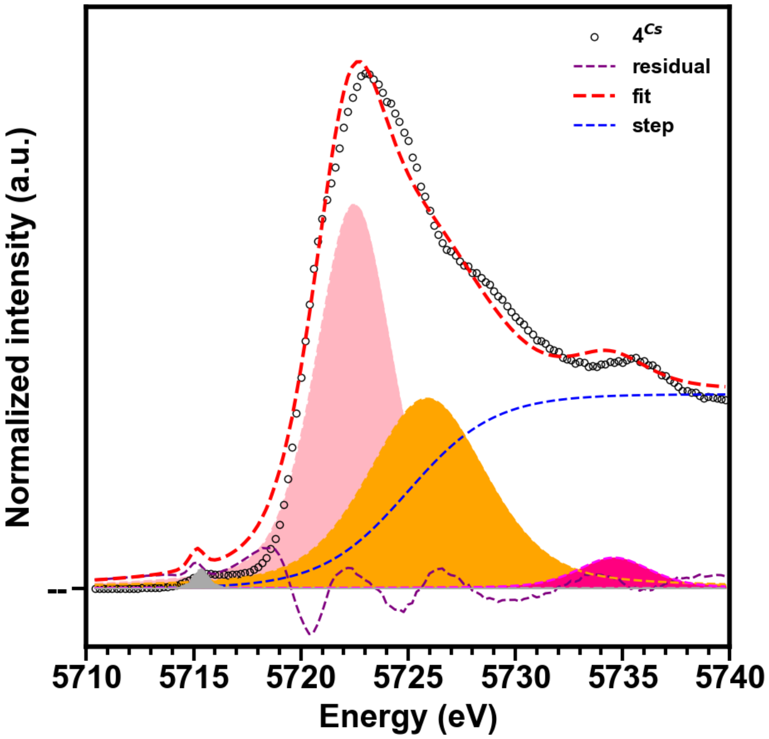

Figure S42. Ce L_3-edge HERFD-XANES fit for $\mathbf{4^{Cs}}$. The features are showing in the following colors: pre-edge feature (p1)= grey, p2 = pink, p3 = orange, p4= magenta, overall sum of fits are shown in red, and residuals are shown in purple. See below for further discussion on the features.

Discussion on HERFD-XANES spectrum of 4^{Cs}:

HERFD-XANES spectrum of $\mathbf{4}^{\mathbf{Cs}}$ shown in **Figure S42** is fitted with 4 peaks(p1,p2,p3,p4). p1 corresponds to the pre-edge feature, and the white-line feature is fitted using two peaks(p2 and p3). Fitting two peaks is necessary to obtain satisfactory fits given the asymmetric peak shape of the white-line feature in the spectrum of $\mathbf{4}^{\mathbf{Cs}}$. There is another feature observed at higher energy, indicated as p4. The asymmetric nature of the white-line feature as well as the presence of higher energy peak shown as p4 are consistent with what has been widely seen in HERFD-XANES spectra of trivalent lanthanides across the series.[27] The origin of higher energy peak (p4) has been attributed to the d-symmetry of photoelectrons that are excited to the continumm.[24]

Table S23. Fit parameters for Ce L_3-edge HERFD-XANES of 4^{Cs}.

Complex	Pre-edge Energy (eV)	Pre-edge Intensity	p2 Energy (eV)	p2 intensity	p3 Energy	p3 Intensity	p4 Energy	p4 intensity
$\mathbf{4}^{\mathbf{Cs}}$	5715.3(3)	0.1(fixed)	5722.5(1)	10(2)	5725.9(8)	8(2)	5734.6(4)	0.8(1)

Table S24. Fit parameters for Ce L_3-edge HERFD-XANES. Intensity of the step function was fixed to 1 and sigma was fixed to 3 throughout the series.

Complex	Step Center (eV)	Pre-edge Energy(eV)	pre-edge Intensity	σ	Peak A Energy(eV)	Peak A Intensity	σ	Peak A' Energy(eV)	Peak A' Intensity	σ	Peak B Energy(eV)	Peak B Intensity	σ
**1**	5730(10)	5717.93(9)	0.2(2)	0.7(1)	5724.30(3)	3(1)	1.31(9)	5728.1(4)	12(8)	3.4(7)	5735.8(1)	7(3)	2.6(2)
**2**	5736(4)	5718.8(2)	0.1(1)	0.7(2)	5724.42(4)	4(1)	1.42(8)	5728.50(7)	12(2)	2.8(1)	5735.6(1)	10(5)	3.2(4)
**3**	5736(2)	5718.0(1)	0.3(1)	0.8(1)	5724.52(2)	2.3(8)	1.14(7)	5728.1(1)	15(1)	3.4(1)	5735.88(7)	9(3)	3.2(2)
**4**	5727(1)	5717.79(7)	0.22(7)	0.76(9)	5724.37(1)	3.3(4)	1.14(2)	5727.76(3)	16(1)	2.79(5)	5735.82(3)	10.7(2)	3.36(4)
**4-I**	5730(4)	5717.50(6)	0.3(1)	0.86(8)	5723.61(2)	5.2(7)	1.76(4)	5727.9(2)	13(3)	3.7(2)	5735.62(6)	8(1)	2.8(1)
**4-Bn**	5730(4)	5717.81(7)	0.3(1)	0.9(1)	5724.47(1)	3.0(3)	1.11(3)	5727.64(7)	19(3)	3.1(1)	5735.6(1)	11(2)	3.3(1)
**4-Npt**	5730(3)	5717.80(8)	0.2(1)	0.9(1)	5724.42(1)	2.8(5)	1.14(4)	5727.71(8)	16(3)	3.0(1)	5735.7(1)	10(1)	3.27(8)

Table S25. Fit parameters for Ce L_3-edge HERFD-XANES of CeO_2, corresponding to the spectrum collected at SSRL BL15-2, shown in Figure S33, left.

Complex	Pre-edge Energy(eV)	pre-edge Intensity	σ	Energy(eV)	Peak A Intensity	σ	Energy(eV)	Peak A' Intensity	σ	Peak B Energy(eV)	Peak B Intensity	σ	Peak B' Energy(eV)	Peak B' Intensity	σ
CeO_2	5718.03(9)	0.16(9)	0.6(1)	5724.43(1)	3.6(4)	0.96(2)	5728.49(2)	12(1)	2.34(6)	5735.9(1)	10(1)	3.5(2)	5739.55(5)	0.6(1)	0.82(9)

References

(1) Gompa, T. P.; Rice, N. T.; Russo, D. R.; Aguirre Quintana, L. M.; Yik, B. J.; Bacsa, J.; La Pierre, H. S. Diethyl Ether Adducts of Trivalent Lanthanide Iodides. *Dalton Trans.* **2019**, *48* (23), 8030–8033.

(2) Johnson, S. A.; Kiernicki, J. J.; Fanwick, P. E.; Bart, S. C. New Benzylpotassium Reagents and Their Utility for the Synthesis of Homoleptic Uranium(IV) Benzyl Derivatives. *Organometallics* **2015**, *34* (12), 2889–2895.

(3) Sheldrick, G. M. SHELXT – Integrated Space-Group and Crystal-Structure Determination. *Acta Crystallogr. Sect. Found. Adv.* **2015**, *71* (1), 3–8.

(4) Dolomanov, O. V.; Bourhis, L. J.; Gildea, R. J.; Howard, J. a. K.; Puschmann, H. OLEX2: A Complete Structure Solution, Refinement and Analysis Program. *J. Appl. Crystallogr.* **2009**, *42* (2), 339–341.

(5) Sheldrick, G. M. Crystal Structure Refinement with SHELXL. *Acta Crystallogr. Sect. C Struct. Chem.* **2015**, *71* (1), 3–8.

(6) APEX2 Suite for Crystallographic Software, Bruker, 2012.

(7) SAINT, Bruker, 2016.

(8) SADABS-2016/2, Bruker, 2016.

(9) Rice, N. T.; Su, J.; Gompa, T. P.; Russo, D. R.; Telser, J.; Palatinus, L.; Bacsa, J.; Yang, P.; Batista, E. R.; La Pierre, H. S. Homoleptic Imidophosphorane Stabilization of Tetravalent Cerium. *Inorg. Chem.* **2019**, *58* (8), 5289–5304.

(10) Rice, N. T.; Popov, I. A.; Russo, D. R.; Gompa, T. P.; Ramanathan, A.; Bacsa, J.; Batista, E. R.; Yang, P.; La Pierre, H. S. Comparison of Tetravalent Cerium and Terbium Ions in a Conserved, Homoleptic Imidophosphorane Ligand Field. *Chem. Sci.* **2020**, *11* (24), 6149–6159.

(11) Boggiano, A. C.; Chowdhury, S. R.; Roy, M. D.; Bernbeck, M. G.; Greer, S. M.; Vlaisavljevich, B.; La Pierre, H. S. A Four-Coordinate Pr4+ Imidophosphorane Complex. *Angew. Chem. Int. Ed. n/a* (n/a), e202409789.

(12) Tateyama, H.; Boggiano, A. C.; Liao, C.; Otte, K. S.; Li, X.; La Pierre, H. S. Tetravalent Cerium Alkyl and Benzyl Complexes. *J. Am. Chem. Soc.* **2024**, *146* (15), 10268–10273.

(13) Pollak, P.; Weigend, F. Segmented Contracted Error-Consistent Basis Sets of Double- and Triple-ζ Valence Quality for One- and Two-Component Relativistic All-Electron Calculations. *J. Chem. Theory Comput.* **2017**, *13* (8), 3696–3705.

(14) Krishnan, R.; Binkley, J. S.; Seeger, R.; Pople, J. A. Self-consistent Molecular Orbital Methods. XX. A Basis Set for Correlated Wave Functions. *J. Chem. Phys.* **1980**, *72* (1), 650–654.

(15) McLean, A. D.; Chandler, G. S. Contracted Gaussian Basis Sets for Molecular Calculations. I. Second Row Atoms, Z=11–18. *J. Chem. Phys.* **1980**, *72* (10), 5639–5648.

(16) Clark, T.; Chandrasekhar, J.; Spitznagel, G. W.; Schleyer, P. V. R. Efficient Diffuse Function-Augmented Basis Sets for Anion Calculations. III. The 3-21+G Basis Set for First-Row Elements, Li–F. *J. Comput. Chem.* **1983**, *4* (3), 294–301.

(17) Spitznagel, G. W.; Clark, T.; von Ragué Schleyer, P.; Hehre, W. J. An Evaluation of the Performance of Diffuse Function-Augmented Basis Sets for Second Row Elements, Na-Cl. *J. Comput. Chem.* **1987**, *8* (8), 1109–1116.

(18) Hehre, W. J.; Stewart, R. F.; Pople, J. A. Self-Consistent Molecular-Orbital Methods. I. Use of Gaussian Expansions of Slater-Type Atomic Orbitals. *J. Chem. Phys.* **2003**, *51* (6), 2657–2664.

(19) Boggiano, A. C.; Studvick, C. M.; Steiner, A.; Bacsa, J.; Popov, I. A.; La Pierre, H. S. Structural Distortion by Alkali Metal Cations Modulates the Redox and Electronic Properties of Ce^{3+} Imidophosphorane Complexes. *Chem. Sci.* **2023**, *14* (42), 11708–11717.
(20) Mooney, J.; Kambhampati, P. Get the Basics Right: Jacobian Conversion of Wavelength and Energy Scales for Quantitative Analysis of Emission Spectra. *J. Phys. Chem. Lett.* **2013**, *4* (19), 3316–3318.
(21) Newville, M.; Otten, R.; Nelson, A.; Stensitzki, T.; Ingargiola, A.; Allan, D.; Fox, A.; Carter, F.; Michał; Osborn, R.; Pustakhod, D.; Lneuhaus; Weigand, S.; Aristov, A.; Glenn; Deil, C.; Mgunyho; Mark; Hansen, A. L. R.; Pasquevich, G.; Foks, L.; Zobrist, N.; Frost, O.; Stuermer; Azelcer; Polloreno, A.; Persaud, A.; Nielsen, J. H.; Pompili, M.; Eendebak, P. Lmfit/Lmfit-Py: 1.2.2, **2023**. https://doi.org/10.5281/ZENODO.8145703.
(22) Sokaras, D.; Weng, T.-C.; Nordlund, D.; Alonso-Mori, R.; Velikov, P.; Wenger, D.; Garachtchenko, A.; George, M.; Borzenets, V.; Johnson, B.; Rabedeau, T.; Bergmann, U. A Seven-Crystal Johann-Type Hard x-Ray Spectrometer at the Stanford Synchrotron Radiation Lightsource. *Rev. Sci. Instrum.* **2013**, *84* (5), 053102.
(23) Solé, V. A.; Papillon, E.; Cotte, M.; Walter, Ph.; Susini, J. A Multiplatform Code for the Analysis of Energy-Dispersive X-Ray Fluorescence Spectra. *Spectrochim. Acta Part B At. Spectrosc.* **2007**, *62* (1), 63–68.
(24) Scheinost, A. C.; Claussner, J.; Exner, J.; Feig, M.; Findeisen, S.; Hennig, C.; Kvashnina, K. O.; Naudet, D.; Prieur, D.; Rossberg, A.; Schmidt, M.; Qiu, C.; Colomp, P.; Cohen, C.; Dettona, E.; Dyadkin, V.; Stumpf, T. ROBL-II at ESRF: A Synchrotron Toolbox for Actinide Research. *J. Synchrotron Radiat.* **2021**, *28* (1), 333–349.
(25) Kvashnina, K. O.; Scheinost, A. C. A Johann-Type X-Ray Emission Spectrometer at the Rossendorf Beamline. *J. Synchrotron Radiat.* **2016**, *23* (3), 836–841.
(26) Kvashnina, K. O. Electronic-Structure Interpretation: How Much Do We Understand Ce L_3 XANES? *Chem. – Eur. J.* **2024**, 30 (46), e202400755.
(27) Zasimov, P.; Amidani, L.; Retegan, M.; Walter, O.; Caciuffo, R.; Kvashnina, K. O. HERFD-XANES and RIXS Study on the Electronic Structure of Trivalent Lanthanides across a Series of Isostructural Compounds. *Inorg. Chem.* **2022**, 61, 4, 1817–1830.
